# On the Information Obtainable from Comparative Judgments

**DOI:** 10.1007/s11336-022-09843-z

**Published:** 2022-02-08

**Authors:** Paul-Christian Bürkner

**Affiliations:** Cluster of Excellence SimTech, University of Stuttgart, Stuttgart, Germany

**Keywords:** Comparative judgments, forced-choice format, faking resistance, Thurstonian IRT models, ordinal models, optimal experimental design

## Abstract

Personality tests employing comparative judgments have been proposed as an alternative to Likert-type rating scales. One of the main advantages of a comparative format is that it can reduce faking of responses in high-stakes situations. However, previous research has shown that it is highly difficult to obtain trait score estimates that are both faking resistant and sufficiently accurate for individual-level diagnostic decisions. With the goal of contributing to a solution, I study the information obtainable from comparative judgments analyzed by means of Thurstonian IRT models. First, I extend the mathematical theory of ordinal comparative judgments and corresponding models. Second, I provide optimal test designs for Thurstonian IRT models that maximize the accuracy of people’s trait score estimates from both frequentist and Bayesian statistical perspectives. Third, I derive analytic upper bounds for the accuracy of these trait estimates achievable through ordinal Thurstonian IRT models. Fourth, I perform numerical experiments that complement results obtained in earlier simulation studies. The combined analytical and numerical results suggest that it is indeed possible to design personality tests using comparative judgments that yield trait scores estimates sufficiently accurate for individual-level diagnostic decisions, while reducing faking in high-stakes situations. Recommendations for the practical application of comparative judgments for the measurement of personality, specifically in high-stakes situations, are given.

**Supplementary Information:**

The online version contains supplementary material available at 10.1007/s11336-022-09843-z.

Likert-type rating scales are still by far the most popular choice to measure personality traits. However, they are susceptible to several response biases that threaten the validity and accuracy of the obtained traits scores, including acquiescence, extremity/centrality biases, or leniency tendencies (Paulhus & Vazire, 2007; Wetzel et al., 2016). As a potential remedy, questionnaires that employ *comparative judgments* between two or more alternative items have gained a lot of attention (Brown & Maydeu-Olivares, 2013; Cheung & Chan, 2002; Paulhus, e.g., 1991; Saville & Willson, 1991). This is true specifically for *forced choice* (FC) formats where the comparative judgments can be expressed as a set of binary decisions on item pairs, forcing respondents to either endorse one or the other item (e.g., Brown & Maydeu-Olivares, 2011; Hontangas et al., 2015). Perhaps most importantly, FC formats and comparative judgments more generally have the potential to reduce faking of responses, as they prevent all items from being endorsed maximally at the same time (Cao & Drasgow, 2019; Wetzel et al., 2016). Faking tendencies tend to occur particularly in high-stakes situations, such as personal selection, where an individual’s responses and the subsequently estimated trait scores are used for inter-individual decision making (Cao & Drasgow, 2019). If the faking tendency varies across individuals in a given population, this will strongly bias the obtained trait scores obtained from any fakeable questionnaire, thus invalidating their use for individual-level diagnostic decisions. Accordingly, developing faking-resistant personality questionnaires that yield highly accurate trait estimates even in high-stakes situations would be a major breakthrough for the fields of psychological diagnostics, differential psychology, and their areas of application.

In the most simple case, scoring such comparative tests proceeds by counting how often each of the items is endorsed, a procedure often referred to as *classical scoring*. For a long time, the lack of alternatives to the classical scoring procedure has been a major barrier to the application of comparative judgments in the context of personality measurement (Baron, 1996; Hicks, 1970). This is because the classical scoring implies the within-person score mean across traits to be fixed by design of the scoring rule (Baron, 1996). Hence, we obtain only *ipsative* trait estimates that enable comparisons within but not across individuals. For example, we can compare extraversion to emotional stability of Person A but not extraversion of Person A to extraversion of Person B. Of course, for individual-level diagnostic decisions, we need *normative* trait scores that enable comparisons both within and across individuals.

## Obtaining Normative Traits Scores from Comparative Judgments

The *Thurstonian Item Response Theory* (TIRT) model has been proposed as a way to obtain normative trait scores from FC questionnaires (Brown & Maydeu-Olivares, 2011; 2012). It is perhaps the most widely applied IRT model for FC data and belongs to a wider class of models that all aim to obtain normative trait scores from such data (see Brown, 2016a for an overview and unifying framework). Although these models differ from each other in the details of how the latent person and item parameters relate to the comparative responses, they all share the same mechanism by which they can achieve normative scoring: *differential weighting of responses*. When responses are differentially weighted, the within-person score means may vary across individuals and so between-person comparisons become possible, provided that the weighting itself is valid. From the perspective of (latent) linear factor analysis models, including TIRT models (Brown, 2016a), there are two ways to achieve differential weighting: First, ensure that factor loadings differ between the compared items. Second, invert some of the items so that positively keyed items are also compared to negatively keyed items, so-called unequally keyed item pairs (Bürkner et al., 2019a). The second mechanism can be understood as an extreme case of the former because inverted items have, by definition, negative factor loadings, which implies particularly strong factor loading differences when compared to items with positive factor loadings.

Various simulation studies have demonstrated that normative scores can indeed be obtained by means of TIRT (or comparable) modeling approaches (Brown & Maydeu-Olivares, 2011; Bürkner et al., 2019a; Lee & Smith, 2020; Schulte et al., 2020), although satisfactory estimation accuracy cannot easily be achieved under all practically relevant conditions (see Sect. 1.1 for details). Several real-world studies have compared TIRT-scored FC questionnaires to rating scales and have found that FC estimates correlate substantially with corresponding rating scale estimates (Guenole et al., 2018; Lee et al., 2018; Watrin et al., 2019). Where investigated, validities were also mostly similar between the two formats (Anguiano-Carrasco et al., 2015; Brown & Bartram, 2013; Lee et al., 2018; Watrin et al., 2019). At least, no consistent pattern favoring one over the other format could be found. With regard to fakability, meta-analytic evidence indicates that score inflation between honest and faking conditions can be lower for FC than for rating scale estimates if the FC comparisons are set up with faking resistance in mind (Cao & Drasgow, 2019). However, it remains unclear if score inflation alone captures all relevant aspects of fakability (Schulte et al., 2021). More detailed literature reviews on applications of FC questionnaires can be found in Bartram (2007) and Cao and Drasgow (2019).

As the responses to FC item pairs or blocks can be represented by a set of binary indicators, they contain comparable little information about the model parameters. To increase the information per pairwise comparison, generalizations of the original (binary) TIRT model for the analysis of other comparative judgment formats have been proposed as well. This includes models for proportion-of-total (compositional) formats (Brown, 2016b) and for (ordinal) graded paired comparisons (Brown & Maydeu-Olivares, 2018, see also Sect. 2). Regardless of the specific response format, the mechanism by which normative scores can be obtained from comparative judgments (i.e., differential weighting of responses) remains the same.

### The Paradox of Comparative Judgments in High-Stakes Situations

In order for comparative judgments to have the potential to be faking resistant in high-stakes situations, the items being compared need to be equally socially desirable (Bürkner et al., 2019a; Wetzel et al., 2020). Otherwise, we can expect almost all individuals to choose the more socially desirable option independent of their true personally traits (Bürkner et al., 2019a), which is indeed what happens in practice (e.g., Schulte et al., 2021). This response behavior is of course understandable, but de-facto renders responses on pairs of items with differing social desirability completely uninformative. Designing pairs of equally keyed items that are equally socially desirable is by no means trivial and requires careful item design and pretesting under realistic conditions, but it can be done (e.g., Wetzel et al., 2020). Trying to achieve the same for unequally keyed pairs is much more complicated though: Items keyed in the objectively less desirable direction would have to appear as equally desirable as items keyed the objectively more desirable direction (Bürkner et al., 2019a).

On the other hand, existing research suggests that, with few exception discussed below, including unequally keyed item pairs is required to obtain normative trait scores from FC questionnaires (Brown & Maydeu-Olivares, 2011; Bürkner et al., 2019a; Lee & Smith, 2020; Schulte et al., 2020). Otherwise, estimated trait scores remain partially ipsative and have insufficient accuracy for use in individual-level diagnostic decisions. Accordingly, we are stuck between two bad options leading to the same outcome in the end: Either include unequally keyed item pairs and risk them being completely uninformative in practice, or directly include only equally keyed item pairs and still end up with highly inaccurate trait scores.

How can we solve this paradox? Two potential paths toward a solution have been identified. First, we can develop unequally keyed item pairs where both items have roughly the same social desirability and can thus be reasonably applied in high-stakes situations (see Wetzel et al., 2020 for some initial evidence in this direction). Second, we can carefully design tests consisting only of equally keyed item pairs so that they alone are sufficient to ensure satisfactory estimation accuracy. It is this second path that I will focus on below, approaching it mainly from a statistical perspective. For example, how shall we choose factor loadings in purely equally keyed designs to maximize information on the trait scores? This is a question of optimal design (see Sect. 1.2). Also, how can we reduce the information lost through the binary decision (or ranking) process that is used in FC formats? This leads to the idea of using rating scales to indicate the *degree* of preference for one or the other item, instead of giving respondents only a binary choice to express their preferences. Modeling the degree of preferences in turn implies the application of ordinal models of comparative judgments (Brown & Maydeu-Olivares, 2018, see Sect. 2 for details). Intuitively, using an ordinal comparative rating with more than two possible response categories, and a corresponding ordinal model, should yield more information about trait scores than a binary decision. This is indeed the case, as shown in Online Supplement A.

Another direction leading along the second path is the observation initially made by Baron (1996) that measuring a higher number of traits can lead to a noticeable increase in estimation accuracy of trait scores. This can go up to a point where sufficient to excellent accuracy can be achieved using only equally keyed item pairs (see Bürkner et al., 2019a; Schulte et al., 2020 for extensive simulations with up to 30 traits). While Baron (1996) provided some explanation for this behavior (see Online Supplement E), the understanding of why higher number of traits can improve estimation accuracy is still incomplete and required further investigation (see Sect. 4.3).

In summary, there remain a lot of open research questions related to the applicability of comparative judgments to measure personality in high-stakes situations. Although the present research was motivated primarily by these questions, the obtained results apply to comparative judgments more generally independent of the specific application context.

### Optimal Design in IRT

In TIRT models and IRT more generally, we aim for an efficient and accurate estimation of person and/or item parameters. Toward this goal, applying principles of *optimal (experimental) design* can be highly beneficial (Atkinson et al., 2007). In the context, one usually distinguishes between two types of optimal design problems: *optimal test designs* and *optimal sampling designs*. In the former, we select items with specific properties for the efficient estimation of person parameters. In the latter, we select people with specific trait scores for the efficient estimation of item parameters. The designs studied in this paper are all optimal test designs, that is, item parameters are treated as known and, at least for the purpose of mathematical argumentation, as freely selectable in order to optimize the efficiency of person parameter estimation. In the literature, optimal designs are investigated from both frequentist and Bayesian perspectives, and I use both perspectives in the present paper as well (see Sect. 3.1 and Online Supplement B, respectively).

In order to quantify the amount of information contained in data $y$ about model parameters $\eta$, optimal design utilizes the *Fisher information matrix* (or simply *Fisher information*) that is generally defined as

1 $$\begin{aligned} \mathbb {I}(\eta ) := \mathbb {E}_y \left[ \frac{d \, l(\eta )}{d \, \eta } \frac{d \, l(\eta )}{d \, \eta ^{\mathop {\mathrm {T}}}} \right] , \end{aligned}$$

where $l(\eta )$ denotes the log-likelihood of the model evaluated at parameter values $\eta$. In words, the Fisher information is the square of the log-likelihood’s gradient with respect to the parameters in expectation over possible data (Lehmann & Casella, 2006). The Fisher information plays a crucial role in both frequentist and Bayesian statistics and constitutes an important tool to study theoretical properties of models. For example, in frequentist statistics, the Fisher information is the inverse of the covariance matrix of an (asymptotically) efficient estimator (Lehmann & Casella, 2006). Understanding the Fisher information of a model provides important insights about how accurately parameters can be estimated from a given study design. Thus, the Fisher information plays a major role also in this paper.

### Summary of Contributions

The primary goal of this paper is to enable accurate and efficient estimation of people’s latent traits using models of comparative judgments, while keeping an eye specifically on the applicability under high-stakes situations. Toward achieving this goal, the paper contributes to the psychological and statistical literature in several ways: First, I extend the mathematical theory of ordinal comparative judgment models with a specific focus on TIRT models (Sect. 2.1 and Online Supplement A). Second, I provide optimal test designs for comparative judgments that maximize estimation accuracy of people’s traits from both frequentist and Bayesian statistical perspectives (Sect. 3.1 and Online Supplement B, respectively). Third, I derive analytic upper bounds for the accuracy of these trait estimates achievable through ordinal comparative judgments and corresponding TIRT models (Sect. 3.3). Fourth, I perform numerical experiments that complement results obtained in earlier simulation studies (Sect. 4) and specifically explain why measuring a higher number of traits can be beneficial for estimation accuracy (Sect. 4.3). Fifth and lastly, I extend recommendations for the practical application of paired comparisons for the measurement of personality, specifically in high-stakes situations (Sect. 5). All mathematical proofs are provided in Appendix A and materials required to replicate the numerical results can be found on OSF (https://osf.io/2g76w/). The online supplement containing additional analytical results and numerical experiments can also be found on OSF.

## Ordinal Thurstonian IRT Models

Building on Thurstone’s law of comparative judgment (Thurstone, 1927), TIRT models are used to describe individuals’ responses on item pairs (or item blocks represented by a set of item pairs) using a latent variable approach (Brown & Maydeu-Olivares, 2011; 2012). Under a Thurstonian model, we assume that each item $i$ has a latent utility $u_{pi}$ that describes the item’s psychological value or desirableness for person $p$. Assuming a one-dimensional linear factor structure for each item (Brown & Maydeu-Olivares, 2011; Bürkner et al., 2019a), such that item $i$ loads on trait $t$, we define

2 $$\begin{aligned} u_{pi} := \lambda _{i} \eta _{pt} + \varepsilon _{pi} \end{aligned}$$

where $\eta _{pt}$ is the trait score of person $p$ on trait $t$, $\lambda _i$ is the factor loading of the item $i$, and $\varepsilon _{pi}$ is the person and item-specific unique factor considered an error term. In a pairwise comparative format, the utilities of two items $i_1$ and $i_2$ are subtracted to yield the latent response $\tilde{y}_{p n}$ of person $p$ on item pair $n$:

3 $$\begin{aligned} \tilde{y}_{p n} := u_{p i_1[n]} - u_{p i_2[n]} = (\lambda _{i_1[n]} \eta _{p,t_1[n]} + \varepsilon _{p,i_1[n]}) - (\lambda _{i_2[n]} \eta _{p,t_2[n]} + \varepsilon _{p,i_2[n]}), \end{aligned}$$

where $i_1[n]$ and $i_2[n]$ denote the $1\mathrm{st}$ and $2\mathrm{nd}$ item belonging to the $n\mathrm{th}$ pair, which load on the trait $t_1[n]$ and $t_2[n]$, respectively. Overall, a total number of $T$ traits is measured. The unique factors $\varepsilon _{pi}$ are assumed to be normally distributed with mean zero and standard deviation $\psi _{i}$. The corresponding variance $\psi ^2_{i}$ is called the uniqueness of the $i\mathrm{th}$ item. Item parameters can be standardized, without loss of information in person parameters, by setting $\psi ^2_{i} = 1 - \lambda ^2_{i}$. I will use standardized item parameters throughout in this paper. Person parameters $\eta _p$ are assumed to be normally distributed with mean 0 and covariance matrix $\Sigma$. The covariance matrix is denoted as $\Phi$ in Brown and Maydeu-Olivares (2011), but I use $\Sigma$ here instead, because the cumulative distribution function of the standard normal distribution is also denoted as $\Phi$. For identification, the marginal variances of $\eta _p$ are fixed to 1 so that $\Sigma$ is also the correlation matrix of $\eta _p$. As a result of these assumptions, $\tilde{y}_{p n}$ is normal distributed with mean zero and standard deviation $\varphi _n := \sqrt{\psi ^2_{i_1[n]} + \psi ^2_{i_2[n]}}$ (Brown & Maydeu-Olivares, 2011; 2018).

In practice, we can never observe $\tilde{y}_{p n}$ directly but only its categorized version $y_{p n}$ that is the response of person $p$ to item pair $n$ on a binary or ordinal (Likert) scale (Brown & Maydeu-Olivares, 2018). Formally, we assume that the observed response $y_{p n}$ arises from the categorization of $\tilde{y}_{pn}$ based on a vector $\tau _n = (\tau _{n1}, \ldots , \tau _{nK})$ of ordered inner thresholds that partition the values of $\tilde{y}_{p n}$ into the $K+1$ observable categories:

4 $$\begin{aligned} y_{p n} = k \Leftrightarrow \tau _{n(k-1)} < \tilde{y}_{p n} \le \tau _{k} \quad \mathrm{for} \quad 1 \le k \le K+1. \end{aligned}$$

For notational convenience, the outer thresholds are set to $\tau _{n0} = -\infty$ and $\tau _{n(K+1)} = \infty$. Taken all of these assumptions together (Brown & Maydeu-Olivares, 2018), the probability that person $p$ selects response category $y_{p n} = k$ on item pair $n$ is given by

5 $$\begin{aligned} p(y_{p n} = k \mathop {\mathrm {\,|\,}}\eta _p) = \Phi \left( \frac{\tau _{nk} - \Lambda _n \eta _p}{\varphi _n} \right) - \Phi \left( \frac{\tau _{n(k-1)} - \Lambda _n \eta _p}{\varphi _n} \right) , \end{aligned}$$

where $\Lambda _n \eta _p := \lambda _{i_1[n]} \eta _{p,t_1[n]} - \lambda _{i_2[n]} \eta _{p,t_2[n]}$ is the systematic part of $\tilde{y}_{pn}$ and $\Phi$ denotes the cumulative distribution function of the standard normal distribution with corresponding density function $\phi$. The binary TIRT model (Brown & Maydeu-Olivares, 2011; 2012) arises as a special case of the ordinal TIRT model when the comparative judgments have only two response categories (i.e., $K = 1$). Conversely, in the theoretical case of infinite response categories (i.e., $K = \infty$), the ordinal TIRT model becomes linear factor model (3) on the latent continuous response $\tilde{y}$ (Schmidt & Schwabe, 2015). Thus, the ordinal TIRT model bridges the gap between the binary TIRT model as a lower bound and a latent linear factor model as an upper bound (see Online Supplement A for technical details).

Ordinal comparative judgments are typically employed directly in the form of item pairs instead of in blocks of more than two items (Brown & Maydeu-Olivares, 2018; Schulte et al., 2021). Accordingly, in the following, I will assume to have measured item pairs directly. This comes without loss of generality as blocks of more than two items can be expressed equivalently by a set of item pairs (subject to certain constraints on the item parameters, Brown & Maydeu-Olivares, 2011; Bürkner et al., 2019a). As a result, the above formulation of Thurstonian IRT models naturally extends to blocks of items in that such blocks simply increases the total number of item pairs implied by a questionnaire.

### Fisher Information of Thurstonian IRT Models

Below, I will study the Fisher information of ordinal TIRT Models with respect to the person parameters $\eta$ assuming the item parameters $\lambda$, $\psi$, and $\tau$ to be known; an approach used commonly in the literature (Atkinson et al., 2007; van der Linden & Hambleton, 2013). For this purpose, I will rewrite Eq. (5) in terms of an equivalent ordinal regression model (Bürkner & Vuorre, 2019). This simplifies the notation and makes important model properties more visible. Define the design matrix $X \in \mathrm{Matrix}^{N \times T}$ of the regression model as

6 $$\begin{aligned} X_{nt} = {\left\{ \begin{array}{ll} \frac{\lambda _{i_1[n]}}{\varphi _n} &{}\quad \text {if } i_1[n] \ne i_2[n] \text { and item } i_1[n] \text { loads on factor } t \\ -\frac{\lambda _{i_2[n]}}{\varphi _n} &{}\quad \text {if } i_1[n] \ne i_2[n] \text { and item } i_2[n] \text { loads on factor } t \\ \frac{\lambda _{i_1[n]} - \lambda _{i_2[n]}}{\varphi _n} &{}\quad \text {if both item } i_1[n] \text { and } i_2[n] \text { load on factor } t \\ 0 &{}\quad \text {otherwise}, \end{array}\right. } \end{aligned}$$

and define the standardized thresholds as $\alpha _{nk} := \tau _{nk} / \varphi _n$. For notational convenience, I will drop the person index $p$ and simply write $\eta$ in the following unless where specifically required to avoid ambiguity. Then, the ordinal TIRT model can be written equivalently as an ordinal regression model with

7 $$\begin{aligned} p(y_n = k \mathop {\mathrm {\,|\,}}\eta ) = \Phi \left( \alpha _{nk} - X_n \eta \right) - \Phi \left( \alpha _{n(k-1)} - X_n \eta \right) \end{aligned}$$

where $X_n$ is the row vector denoting the $n\mathrm{th}$ row of design matrix $X$. Note that this is simply a rewritten version of Eq. (5). Now define

8 $$\begin{aligned} {\mathop {\mathrm {I}}}_{nK}(\eta ) := \sum _{k = 1}^{K+1} \frac{\left( \phi \left( \alpha _{nk} - X_n \eta \right) - \phi \left( \alpha _{n(k-1)} - X_n \eta \right) \right) ^2}{\Phi \left( \alpha _{nk} - X_n \eta \right) - \Phi \left( \alpha _{n(k-1)} - X_n \eta \right) }, \end{aligned}$$

which I will call the *information factor* (of the $n\mathrm{th}$ item pair based on $K$ thresholds) for reasons that become apparent soon. Using basic calculus, it can be shown that the Fisher information matrix of the person parameters $\eta$ equals

9 $$\begin{aligned} \mathbb {I}_\mathrm{TIRT}(\eta ) = \sum _{n=1}^N {\mathop {\mathrm {I}}}_{nK}(\eta ) X_n^{\mathop {\mathrm {T}}} X_n \end{aligned}$$

(see Brown & Maydeu-Olivares, 2018; Samejima, 1969, or Online Supplement A for derivations). In the limiting case of infinite response categories, $\mathbb {I}_\mathrm{TIRT}(\eta )$ will converge to the information matrix of a normal linear regression model (Schmidt & Schwabe, 2015, see also Online Supplement A). It is well known (e.g., Atkinson et al., 2007) that the Fisher information matrix of the regression coefficients of a normal linear regression model is given by

10 $$\begin{aligned} \mathbb {I}(\eta ) = \sum _{n=1}^N X_n^{\mathop {\mathrm {T}}} X_n \end{aligned}$$

Thus, we obtain the following natural limits of the information factor:

#### Corollary 2.1

Let $\Phi$ be the cumulative distribution function of the standard normal distribution with corresponding density function $\phi$, and let $(s_k)_{0 \le k \le K+1}$ be a series of ordered real values such that $-\infty = s_0 \le s_1 \le \ldots \le s_K \le s_{K+1} = \infty$. Then, for finite *K*, the following inequalities hold:

11 $$\begin{aligned} 0 \le \sum _{k=1}^{K+1} \frac{(\phi (s_k) - \phi (s_{k-1}))^2}{\Phi (s_k) - \Phi (s_{k-1})} < 1. \end{aligned}$$

Moreover, if $\lim _{K \rightarrow \infty } \Phi (s_k) - \Phi (s_{k-1}) = 0$ for all $k \in \{1, \ldots , K\}$, then

12 $$\begin{aligned} \sum _{k=1}^{\infty } \frac{(\phi (s_k) - \phi (s_{k-1}))^2}{\Phi (s_k) - \Phi (s_{k-1})} = 1. \end{aligned}$$

**Fig. 1:**
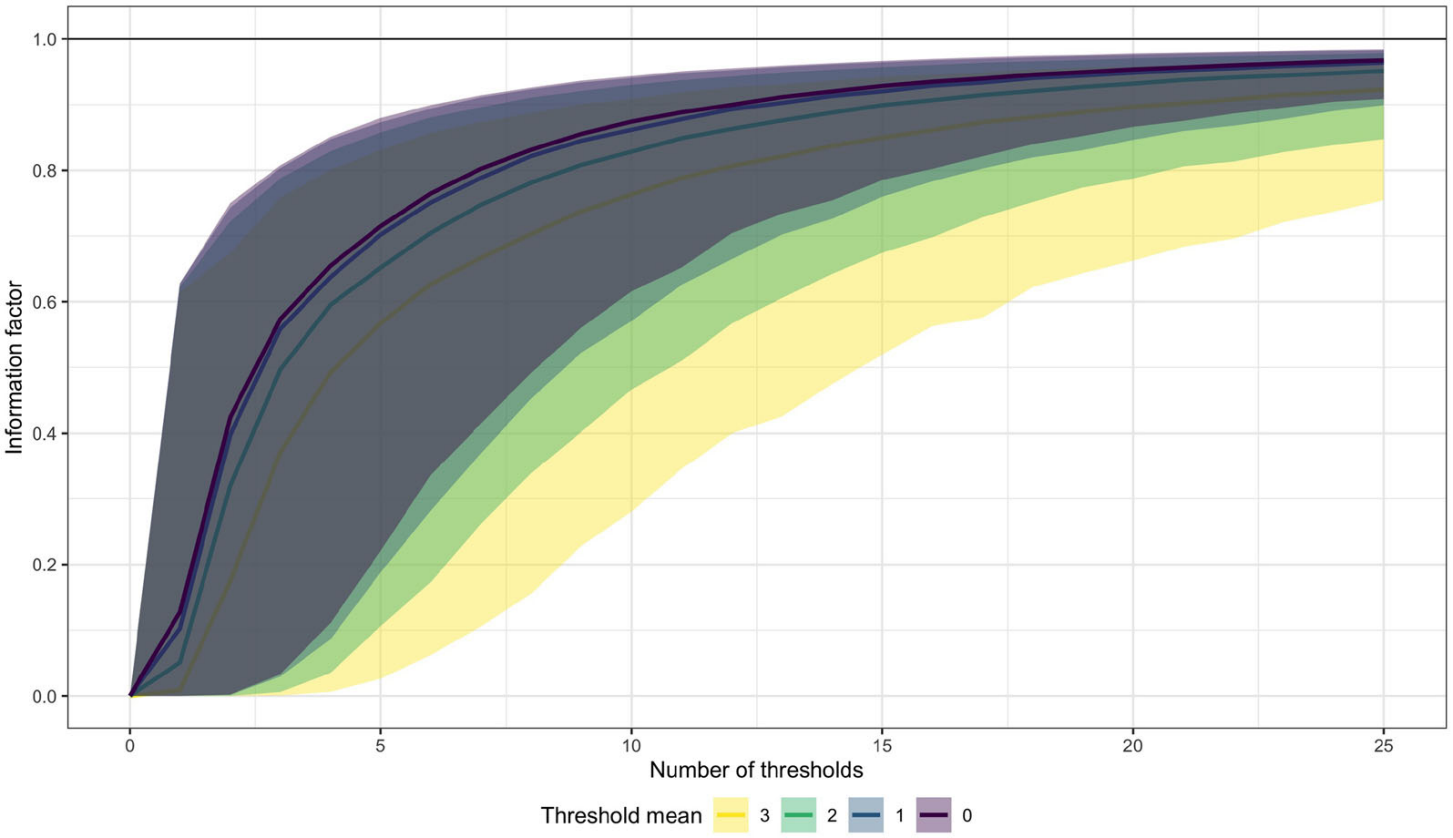
Information factor (Eq. (8)) as a function of the number of thresholds $K$ and threshold mean $\overline{\tau }$. For each condition, 10,000 samples of independent random draws of length $K$ were drawn from a normal distribution with varying mean $\overline{\tau }$ (colored) and standard deviation $3$. Central colored lines indicate medians over all samples (per $K$ and $\overline{\tau }$), while the shaded areas indicate 90% confidence intervals. The black horizontal line denotes the information factor of the latent normal model without categorization (Color figure online).

Corollary 2.1 implies that $1 - {\mathop {\mathrm {I}}}_{nK}(\eta )$ can be interpreted as the *percentage of information lost* through the response categorization process during measurement of item pair $n$. If we could directly observe the latent variable $\tilde{y}_n$ underlying the observed response $y_n$, we would have ${\mathop {\mathrm {I}}}_{nK}(\eta ) = 1$ and no information would be lost through response categorization. Of course, measuring $\tilde{y}_n$ is impossible in reality, but ${\mathop {\mathrm {I}}}_{nK}(\eta )$ still approaches $1$ rather quickly as $K$ increases, provided that the threshold vector $\tau _n$ roughly has mean $0$ (see Figure 1 darker lines). For example, for 10 response categories ($K = 9$), the median information factor across item pairs already exceeds 85% under reasonable assumptions. However, as threshold means differ more from zero, the convergence of ${\mathop {\mathrm {I}}}_{nK}(\eta )$ becomes much slower (see Fig. 1 brighter lines). This is highly relevant for the application of TIRT models in high-stakes situations where social desirability is an issue, as I will elaborate in the Discussion.

**Fig. 2:**
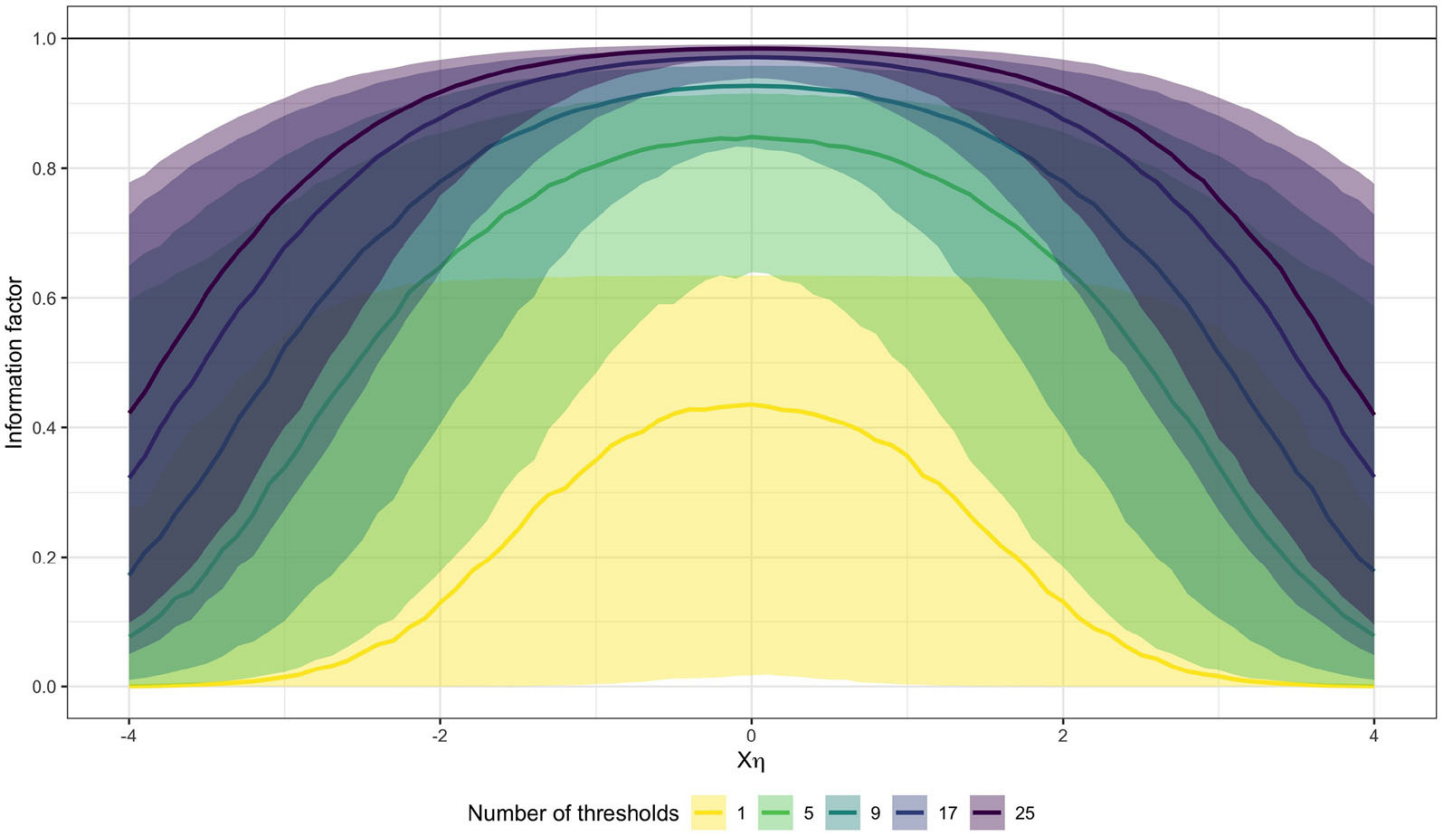
Information factor parameterized as $s_k = \tau _k - X \eta$ as a function of the number of thresholds $K$. For each condition, 10,000 samples of independent random draws for the threshold vector $\tau$ of length $K$ were drawn from a normal distribution with mean 0 and standard deviation $1.5$. Central colored lines indicate medians over all samples per $K$, while the shaded areas indicate 90% confidence intervals. The black horizontal line denotes the information factor of the latent normal model without categorization (Color figure online).

Convergence of the information factor toward $1$ is not uniform across different values of $X_n \eta$ (see Figure 2 for an illustration). Rather, convergence for very small or large $X_n \eta$ values, corresponding to bigger differences between compared trait scores, is slower than for values close to the threshold mean (compare median lines in Fig. 2). Additionally, the variation of the information factor across different threshold vectors, corresponding to different items, is larger for more extreme $X_n \eta$ values (compare shaded areas Fig. 2). Still, using an increasing number thresholds greatly increases the obtainable Fisher information from comparative judgments across the board, which is clearly visible in particular when contrasted with the binary approach (yellow line in Fig. 2).

## Upper Information Bounds

In Corollary 2.1, we have seen that the information from the linear normal model on the latent variable $\tilde{y}$ provides a natural and sharp upper bound for the Fisher information obtainable from ordinal comparative judgments. This is because the latent linear model does not suffer from any loss of information through response categorization. Thus, we can use this model to study the maximal Fisher information obtainable by a given test design. Although the maximal information cannot be fully achieved in practice, we have seen above that a close approximation is very well realistic. What is more, a lot of the central properties of this latent linear model, which I will study analytically below, apply to its ordinal counterparts as well. Accordingly, there is a lot to be learned from studying such an ideal model even if we are not able to fully achieve it in practice.

For the purpose of the upcoming mathematical analysis, I will assume factor loadings $\lambda _i$ and error variances $\psi _i^2$ to be known or at least estimable with sufficient precision so that their uncertainty does not affect person parameter estimates to a relevant degree. Such a precision seems to be achievable already when including data of as few as 300 (or more) individuals in the analysis as suggested by the simulation study of Schulte et al. (2020). When applying IRT models in practice, it is common to measure several hundreds or perhaps even thousands of individuals. Accordingly, in practice, the assumption of known item parameters does hardly affect estimation accuracy obtained for individuals’ trait scores. And even if the information difference was noticeable, assuming item parameters to be known implies more information on the person parameters than when item parameters are estimated. As such, the general goal to provide an upper information bound remains unaffected. Of course, we rarely know the exact values of item parameters in practice; and whether or not item parameters are estimated makes a big difference with regard to the required estimation algorithms and their stability (Bürkner et al., 2019a; e.g., van der Linden & Hambleton, 2013), but these considerations are not in focus of the present paper.

Under the above assumptions, we can formally write down the likelihood of the latent normal model on $\tilde{y}$ as a linear regression

13 $$\begin{aligned} \tilde{y}_n \sim \mathrm{normal}(X_n \eta , 1), \end{aligned}$$

where $n = 1, \ldots , N$ indexes item pairs and $X_n$ denotes the $n\mathrm{th}$ row of the test design matrix $X$ defined in Eq. (6). In Sect. 2, it was mentioned the person parameters $\eta$ are assumed to come from a multivariate normal distribution, an assumption made for two reasons: (a) Fixing the marginal variance of this distribution ensures joint identification of item and person parameters and (b) modeling the correlation matrix of this distribution enables sharing of information across parameters of the same person as well as across people. Reason (a) is obsolete when assuming item parameters to be known. However, reason (b) remains highly relevant and will be discussed in detail in Sect. 3.2. For now, I will not consider such a distribution or equivalently, assume it to have infinite marginal variances.

### Maximizing the Test Design Information

As already mentioned earlier, the Fisher information matrix of the regression coefficients of a linear model is given by

14 $$\begin{aligned} M := \mathbb {I}(\eta ) = \sum _{n=1}^N X_n^{\mathop {\mathrm {T}}} X_n = X^{\mathop {\mathrm {T}}} X. \end{aligned}$$

It is well known (e.g., Atkinson et al., 2007) that $\Sigma _{\hat{\eta }} := M^{-1}$ is the covariance matrix of the maximum likelihood estimator

15 $$\begin{aligned} \hat{\eta }_\mathrm{ML} = M^{-1} X^{\mathop {\mathrm {T}}} \tilde{y}. \end{aligned}$$

Thus, the larger the information, the smaller the uncertainty in the parameter estimates, as is the case more generally for (asymptotically) efficient estimators (Atkinson et al., 2007). The Fisher information $M$ obtainable on the latent space of $\tilde{y}$ depends on the factor loadings $\lambda _i$ and the uniqueness $\psi _i^2$. As per Eq. (2), both parameters are related to each other via $\psi _i^2 = v_i - \lambda _i^2$ where $v_i$ denotes the variance of the utilities $u_{pi}$ across people. For ordinal paired comparisons, $v_i$ is not identified so we can set $v_i = 1$ without loss of generality, which leads to standardized item parameters and $\psi _i^2 = 1 - \lambda _i^2$. Independently of how we fix the utility variances, $M = M(\lambda )$ can be written to only depend on the factor loadings, which will thus be the primary target of investigation.^1^

We can now ask how to *choose* factor loadings in order to maximize information or equivalently minimize uncertainty. Such questions can be investigated by means of optimal design and several optimality criteria can be applied (see Atkinson et al., 2007; Berger & Wong, 2009 for an overview). Probably the most common criterion is *D-optimality* aiming to maximize the determinant of the Fisher information, $\mathrm{det}(M)$, or equivalently minimize the determinant of the inverse information $\mathrm{det}(M^{-1}) = \mathrm{det}(M)^{-1}$. The popularity of D-optimality can be explained by a combination of mathematical convenience and intuitiveness of interpretation as $\det (M^{-1})$ is proportional to the volume of the $T$-dimensional confidence ellipsoid of an (asymptotically) efficient estimator (Atkinson et al., 2007). As above, $T$ is used to denote the total number of estimated parameters per person, that is, the total number of measured traits. Minimizing $\det (M^{-1})$ can be interpreted as minimizing the joint uncertainty of the estimated trait scores per person. For comparability across different number of traits, it is sensible to define the D-optimality criterion as

16 $$\begin{aligned} C_D(\lambda ) := C_D(M(\lambda )) := \det (M^{-1})^{1/T} = \det (M)^{-1/T}, \end{aligned}$$

which is a strictly monotonic transformation of $\det (M^{-1})$ that accounts for the volume change induced by increased dimensionality. If we assume some symmetry in the design, we obtain an insightful analytical result about the D-optimal test design for comparative judgments.

#### Theorem 3.1

Let $\lambda _\mathrm{max} \in (0, 1)$ be the maximally achievable standardized factor loading and let $\lambda _{n1}$ and $\lambda _{n2}$ be the two standardized factor loadings for item pair $n \in \{1, \ldots , N\}$, with $\lambda _{n1} \ge 0$ without loss of generality. Assume that each trait *i* is compared to every other trait $j \ne i$ an even number of $R_{ij} \ge 0$ times. Further, without loss of generality, assume that every two consecutive pairs *m* and $m+1$, such that *m* is odd, belong to the same trait combination. Then, for any number of traits $T \ge 2$ and any even number of comparisons $R_{ij} \ge 0$ per trait combination the D-optimal design is given if $\lambda _{n1} = \lambda _\mathrm{max}$, $\lambda _{n2} = (-1)^{n}\lambda _\mathrm{max}$, and each trait appears in the same number of item pairs in total.

Among others, this result implies that half of the item pairs should be equally keyed and the other half should be unequally keyed. This is particularly relevant, as it not only states that mixed keyed designs are preferable but also specifies exactly how the ratio between the number of equally and unequally keyed comparisons should be for the test to be optimal. To build an intuition, it is helpful to look at the Fisher information of a single item with trait scores parameterized by their mean $\bar{\eta }$ and difference $\eta _d$ such that $\eta _1 = \bar{\eta } + \eta _d / 2$ and $\eta _2 = \bar{\eta } - \eta _d / 2$. Then, the information in the direction of $\bar{\eta }$ equals

17 $$\begin{aligned} \mathbb {I}(\bar{\eta }) = \frac{(\lambda _1 - \lambda _2)^2}{2 - \lambda _{1}^2 - \lambda _{2}^2}, \end{aligned}$$

while the information in the direction of $\eta _d$ equals

18 $$\begin{aligned} \mathbb {I}(\eta _d) = \frac{(\lambda _1 + \lambda _2)^2}{4 (2 - \lambda _{1}^2 - \lambda _{2}^2)}. \end{aligned}$$

In words, the trait score mean requires high factor loading *differences*, as provided by unequally keyed item pairs, while the trait score difference requires high factor loading *sums*, as provided by equally keyed item pairs.

It might be argued that D-optimality is not an ideal measure for evaluation of comparative judgments designs as we are primarily interested in minimizing the marginal variances of each trait, rather than minimizing the determinant of the whole covariance matrix. In other words, we might be more interested in achieving *A-optimality* (Atkinson et al., 2007). I define

19 $$\begin{aligned} C_A(\lambda ) := C_A(M(\lambda )) := \sqrt{\frac{1}{T} \sum _{i=1}^T (M^{-1})_{ii}} \end{aligned}$$

as the A-optimality criterion, which is a strictly monotonic transformation of the sum of marginal variances. The scaling via $1/T$ ensures that the score is comparable across varying number of traits $T$, and the square-root transform enables interpretation in terms of standard deviations instead of variances. Thus, $C_A$ can be interpreted as an average marginal standard deviation across trait estimates. It turns out that for the comparative judgment designs considered above, the D-optimal design is also A-optimal:

#### Theorem 3.2

Under the assumptions of Theorem 3.1, the D-optimal design is also A-optimal.

Through the proofs of Theorems 3.1 and 3.2, it not only becomes clear that an equal number of equally and unequally keyed pairs is optimal but also *why* this is the case, namely that the diagonal elements of the Fisher information are maximized, while the off-diagonal elements become zero. Conversely, when applying a design with only equally keyed item pairs, we can achieve the same diagonal elements in theory, but obtain highly negative off-diagonal elements, which drastically increases (worsens) both the inverse Fisher information’s determinant (D-optimality) and its trace (A-optimality). In the most extreme case of an equally keyed design, where $\lambda _{n1} = \lambda _{n2}$ for each item pair $n = 1, \ldots , N$, the design no longer even identifies the person parameters (see also Brown, 2016a).

The above-derived optimal design is in fact ‘very optimal’ compared to potentially alternatives as we can see using a simple example. Suppose we have $T = 5$ traits and a symmetric design with $R = 2$ comparisons for each trait combination such that the design consists of a total of $N = 20$ paired comparisons. Suppose further that $\lambda _{n1} = \bar{\lambda } + \lambda _{\Delta } / 2 \in (0, 0.8]$ and $\lambda _{n2} = \bar{\lambda } - \lambda _{\Delta } / 2 \in (0, 0.8]$ for each trait combination, so that $\bar{\lambda }$ is the factor loading mean and $\lambda _{\Delta }$ is the factor loading difference per item pair. In this example, every trait has the same amount of higher and lower factor loadings such that the information for each trait is the same due to symmetry. The corresponding mixed keyed design is obtained by switching the sign of half of the $\lambda _{n2}$ to be negative.

**Fig. 3:**
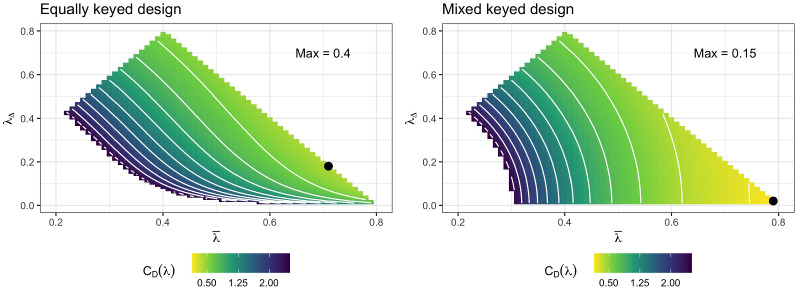
D-optimality criterion for a simple example with $T = 5$ traits and $N = 20$ item pairs as a function of the mean factor loading $\bar{\lambda }$ and factor loading difference $\lambda _{\Delta }$. Equally keyed designs are depicted on the left, while mixed keyed designs are depicted on the right. Brighter colors indicate better values (lower confidence ellipsoid volume). Black dots indicate the location of the optimal design. The left-most part of the grids are not shown to avoid obfuscating the color scale (Color figure online).

**Fig. 4:**
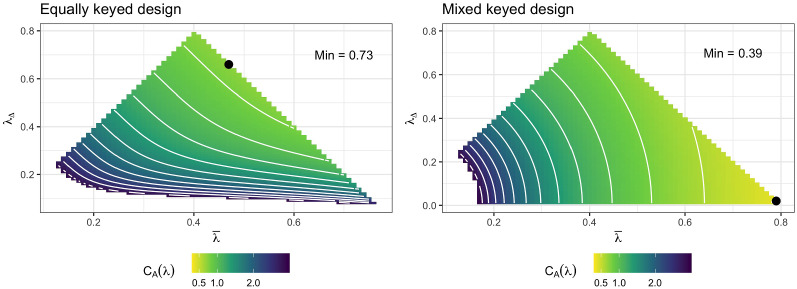
A-optimality criterion for $T = 5$ traits and $N = 20$ item pairs as a function of the mean factor loading $\bar{\lambda }$ and factor loading difference $\lambda _{\Delta }$. Equally keyed designs are depicted on the left, while mixed keyed designs are depicted on the right. Due to symmetry of the design, standard deviations of all traits are identical. Brighter colors indicate better values (lower marginal standard deviations). Black dots indicate the location of the optimal design. The left-most part of the grids are not shown to avoid obfuscating the color scale (Color figure online).

For varying $\bar{\lambda }$ and $\lambda _{\Delta }$, I illustrate the implied D-optimality criterion in Fig. 3. On the right-hand side of Fig. 3, an illustration for the mixed keyed design is shown, which clearly has the highest determinant when the factor loadings are maximal within the considered range ($\bar{\lambda } = 0.8$ and $\lambda _{\Delta } = 0$). In contrast, when considering an equally keyed design (left-hand side of Fig. 3), we see the importance of balancing high mean factor loadings (i.e., high $\bar{\lambda }$) with high differences between factor loadings within the same item pair (i.e., high $\lambda _{\Delta }$). What is more, the optimal equally keyed design offers only a fraction of the information from the (mixed keyed) optimal design ($C_D(M) \approx 0.40$ vs. $C_D(M) \approx 0.15$) under the given conditions. The practical implications of the information difference between mixed and equally keyed designs can be better grasped when investigating the marginal standard deviations of the ML estimator, that is, the A-optimality criterion. When comparing the left-hand and right-hand side of Fig. 4, we not only see very similar optimality patterns as for the D-optimality criterion, but also that the optimal mixed keyed design implies marginal SDs about as half as big as the corresponding marginal SDs implied by the optimal equally keyed design ($C_A(M) \approx 0.37$ vs. $C_A(M) \approx 0.73$). The absolute values are not super small in either case, but this is simply the result of using only $N = 20$ item pairs for the purpose of this illustration. More detailed numerical experiments are provided in Sect. 4.

It is of high practical relevance to understand how the optimal designs on the latent linear TIRT model generalize to ordinal TIRT models. Fortunately, the optimal designs generalize quite nicely:

#### Theorem 3.3

Let $C: \mathrm{Matrix}^{T \times T} \rightarrow \mathbb {R}$ be an optimal design criterion based on the Fisher information such that, without loss of generality, lower values are considered more optimal. Let $M^\star := \sum _{n=1}^N M^\star _n := \sum _{n=1}^N X^{\star \mathop {\mathrm {T}}}_n X^\star _n$ be the optimal Fisher information of a normal linear model according to criterion *C* with corresponding optimal design matrix $X^\star$. Assume that $C(M^\star ) \le C(M)$ implies $C(c \, M^\star ) \le C(c \, M)$, for any Fisher information matrix $M = \sum _{n=1}^N X^{\mathop {\mathrm {T}}}_n X_n$ based on a permissible design matrix *X* and any constant $c \in \mathbb {R}^+$. Then, the optimal design matrix $X^\star$ for the normal linear model on $\tilde{y}$ is also optimal for all corresponding ordinal models on *y* independently of the number of thresholds *K*.

Theorem 3.3 applies very generally to any ordinal model with a normal distribution and a linear predictor on the latent scale. Most relevant for the purposes of this paper, Theorem 3.3 immediately implies the following result:

#### Corollary 3.4

Under the assumptions of Theorem 3.1, the D- and A-optimal factor loadings for the latent linear model on $\tilde{y}$ are also D- and A-optimal for the corresponding ordinal models on *y* independently of the number of thresholds *K*.

However, neither Theorem 3.3 nor Corollary 3.4 make a statement about the optimal ordinal thresholds, just about the optimal factor loadings. Deriving optimal thresholds for ordinal TIRT models is an interesting research direction but out of scope of the present paper. In the following, I will continue with studying the properties of latent linear TIRT models.

### Adding Prior Information

Investigating the test design information alone tells only half of the story even in the linear case. This is because the person parameters in IRT models constitute latent variables that are assumed to come from an underlying distribution describing the variation of the parameters across people (van der Linden & Hambleton, 2013). In particular this is true for the TIRT model that assumes a multivariate normal distribution for the person parameters $\eta$ (see Sect. 2 for details). From a Bayesian perspective, this distribution can be understood as a prior and we can formally extend linear regression model (13) as

20 $$\begin{aligned} \tilde{y}_n&\sim \mathrm{normal}(X_n \eta , 1) \end{aligned}$$

21 $$\begin{aligned} \eta&\sim \mathrm{multinormal}(0, \Sigma _{\mathop {\mathrm {prior}}}) \end{aligned}$$

In practical applications of TIRT models, we would usually treat $\Sigma _{\mathop {\mathrm {prior}}}$ as a hyperparameter that is estimated from the data. Here, again for the purpose of the mathematical analysis, I assume $\Sigma _{\mathop {\mathrm {prior}}}$ to be known rather than estimated, an assumption I will come back to in the Discussion. If $\Sigma _{\mathop {\mathrm {prior}}}$ is known, the above model becomes a special case of Bayesian linear regression. It is well known that a normal prior is conjugate to a normal likelihood (Gelman et al., 2013), and so we can derive the posterior distribution of $\eta$ analytically as

22 $$\begin{aligned} \eta \mathop {\mathrm {\,|\,}}\tilde{y} \sim \mathrm{multinormal}(\mu _{\mathop {\mathrm {post}}}, \Sigma _{\mathop {\mathrm {post}}}) \end{aligned}$$

with posterior covariance matrix

23 $$\begin{aligned} \Sigma _{\mathop {\mathrm {post}}} = \left( M + \Sigma _{\mathop {\mathrm {prior}}}^{-1} \right) ^{-1} \end{aligned}$$

and posterior mean

24 $$\begin{aligned} \mu _{\mathop {\mathrm {post}}} = \Sigma _{\mathop {\mathrm {post}}} M \hat{\eta }_\mathrm{ML}, \end{aligned}$$

where $M = X^{\mathop {\mathrm {T}}} X$ and $\hat{\eta }_\mathrm{ML}$ is the maximum likelihood estimate of $\eta$:

25 $$\begin{aligned} \hat{\eta }_\mathrm{ML} = M^{-1} X^{\mathop {\mathrm {T}}} \tilde{y}. \end{aligned}$$

The posterior mean $\mu _{\mathop {\mathrm {post}}}$ is commonly known as the expected a-posteriori (EAP) estimator and applied extensively both in full and empirical Bayes approaches (Gelman et al., 2013). The expected value and covariance matrix of $\mu _{\mathop {\mathrm {post}}}$ over data $\tilde{y}$ are given, respectively, by

26 $$\begin{aligned} \bar{\mu }_{\mathop {\mathrm {post}}} := \mathbb {E}_{\tilde{y}}(\mu _{\mathop {\mathrm {post}}}) = \Sigma _{\mathop {\mathrm {post}}} M \eta \end{aligned}$$

and

27 $$\begin{aligned} \mathrm{Var}_{\tilde{y}}(\mu _{\mathop {\mathrm {post}}}) = \Sigma _{\mathop {\mathrm {post}}} M \Sigma _{\mathop {\mathrm {post}}}. \end{aligned}$$

The subscript of $\mathbb {E}$ and $\mathrm{Var}$ indicates over which variable integration is performed. In order to measure to accuracy of the EAP estimator, investigating its covariance matrix is insufficient on its own as the estimator may be biased. For this reason, I additionally consider the mean squared error (MSE) matrix

28 $$\begin{aligned} \mathrm{MSE}_{\tilde{y}}(\mu _{\mathop {\mathrm {post}}}, \eta )&:= \mathbb {E}_{\tilde{y}} \left( (\mu _{\mathop {\mathrm {post}}} - \eta ) (\mu _{\mathop {\mathrm {post}}} - \eta )^{\mathop {\mathrm {T}}} \right) \nonumber \\&= \mathrm{Var}_{\tilde{y}}(\mu _{\mathop {\mathrm {post}}}) + \left( \bar{\mu }_{\mathop {\mathrm {post}}} - \eta \right) \left( \bar{\mu }_{\mathop {\mathrm {post}}} - \eta \right) ^{\mathop {\mathrm {T}}} \nonumber \\&= \Sigma _{\mathop {\mathrm {post}}} M \Sigma _{\mathop {\mathrm {post}}} + (\Sigma _{\mathop {\mathrm {post}}} M - I) \eta \eta ^{\mathop {\mathrm {T}}} (\Sigma _{\mathop {\mathrm {post}}} M - I)^{\mathop {\mathrm {T}}}, \end{aligned}$$

which can be expressed as the variance plus the bias squared. The MSE matrix can be used to obtain root-mean-square error (RMSE) estimates as an important measure for predictive accuracy:

29 $$\begin{aligned} \mathrm{RMSE}(\mu _{{\mathop {\mathrm {post}}},i}, \eta _i) = \sqrt{\mathrm{MSE}_{\tilde{y}}(\mu _{\mathop {\mathrm {post}}}, \eta )_{ii}}. \end{aligned}$$

### Marginalizing over Person Parameters

In the above equations, I condition on a fixed vector $\eta$ of true person parameters. In other words, I investigate the estimates of only a single person at the same time. From the perspective of model estimation, this is totally valid as all the item parameters (i.e., $\lambda _i$ and $\psi _i$ per item $i$) and person hyperparameters (i.e., $\Sigma _{\mathop {\mathrm {prior}}}$) were considered to be known for the purpose of the mathematical analysis performed here. If they were not known, data of all people had to be modeled jointly in order to estimate item parameters and person hyperparameters, as is done in the full version of the TIRT model. However, even if the simplified model can be estimated separately per person, we still aim to compare different individuals and hence we need to consider multiple $\eta$ parameters and compare their estimates. This can be done by interpreting the multinormal prior over $\eta$ in Eq. (21) as a sampling distribution from which true person parameter values can be drawn (Gelman et al., 2013). More accurately, one assumes that $\eta$ is sampled according to

30 $$\begin{aligned} \eta \sim \mathrm{multinormal}(0, \Sigma _{\eta }). \end{aligned}$$

This resembles the approach in simulation studies, only that the results below are derived analytically instead of empirically via repeated sampling from the distribution. Because the latent scale of $\eta$ is arbitrary, I set the marginal variances ${\Sigma _{\eta }}_{ii} = 1$ as is standard in TIRT models and many other latent variable models for reasons of identification (Brown & Maydeu-Olivares, 2011). If $\Sigma _{\eta } = \Sigma _{\mathop {\mathrm {prior}}}$, then the model’s prior assumes the correct data generating process. If $\Sigma _{\eta } \ne \Sigma _{\mathop {\mathrm {prior}}}$, then the prior provides some kind of model misspecification, whose influence on the obtained posterior estimates can be investigated. This is sensible in the context of TIRT models, as simulation studies have shown potentially strong biases in the correlation hyperparameters that constitute $\Sigma _{\mathop {\mathrm {prior}}}$ (Brown & Maydeu-Olivares, 2011; Bürkner et al., 2019a); biases we can mimic by letting $\Sigma _{\mathop {\mathrm {prior}}}$ deviate from $\Sigma _{\eta }$.

It is now possible to study $\eta$-depending quantities not only conditional on $\eta$ but rather marginalized over its distribution (30). In particular, we can study the first two moments of $\bar{\mu }_{\mathop {\mathrm {post}}}$ that evaluate to

31 $$\begin{aligned} \mathbb {E}_{\eta } \left( \bar{\mu }_{\mathop {\mathrm {post}}} \right) = \Sigma _{\mathop {\mathrm {post}}} M \mathbb {E}_{\eta } (\eta ) = 0 \end{aligned}$$

and

32 $$\begin{aligned} \mathrm{Var}_{\eta } \left( \bar{\mu }_{\mathop {\mathrm {post}}} \right) = \Sigma _{\mathop {\mathrm {post}}} M \Sigma _{\eta } M \Sigma _{\mathop {\mathrm {post}}}. \end{aligned}$$

Due to the positive semi-definiteness of $\Sigma _{\mathop {\mathrm {prior}}}$, $\Sigma _{\eta }$, and $M$, and because of $\mathrm{det} (\Sigma _{\mathop {\mathrm {post}}}) \le \mathrm{det} (M)$ as implied by Eq. (23), we see that the marginal variances

33 $$\begin{aligned} \mathrm{Var}_{\eta } \left( \bar{\mu }_{\mathop {\mathrm {post}}} \right) _{ii} \le {\Sigma _{\eta }}_{ii} = 1. \end{aligned}$$

Moreover, if the a-priori covariance matrix $\Sigma _{\mathop {\mathrm {prior}}}$ is finite, inequality (33) holds strictly so that the variance of $\bar{\mu }_{\mathop {\mathrm {post}}}$ is smaller than the variance of $\eta$. In other words, the prior leads to a shrinkage of estimates in expectation and thus contributes to the bias and MSE of $\mu _{\mathop {\mathrm {post}}}$, a well-known result of adding prior information (Gelman et al., 2013). This is not to say that shrinkage is undesirable as it also decreases the variance, thus leading to a bias-variance trade-off (Gelman & Hill, 2006). However, in diagnostic practice, we are not directly interested in estimating the true scale of $\eta$ correctly, but to compare (the estimates of) different people’s traits. Accordingly, the difference in the scales of $\bar{\mu }_{\mathop {\mathrm {post}}}$ and $\eta$ is irrelevant and any linear transformation of the true scale will do equivalently well. Also, when fitting TIRT models in practice, we estimate both person parameters and factor loadings simultaneously. This implies that the prior scale is required to identify the scale of $\eta$ and thus no shrinkage will occur in this case.

To remove the shrinkage-induced scale difference, I standardize $\mu _{\mathop {\mathrm {post}}}$ so that its expectation $\bar{\mu }_{\mathop {\mathrm {post}}}$ has the same variance as $\eta$. Formally, this is done as follows: Let $S$ be a diagonal matrix with diagonal elements equal to the inverse of the marginal standard deviations of $\bar{\mu }_{\mathop {\mathrm {post}}}$ over $\eta$, that is,

34 $$\begin{aligned} S_{ii} := 1 / \sqrt{ \mathrm{Var}_{\eta } \left( \bar{\mu }_{\mathop {\mathrm {post}}} \right) _{ii}}. \end{aligned}$$

and define the scaled EAP estimator $\delta _{\mathop {\mathrm {post}}}$ of $\eta$ as

35 $$\begin{aligned} \delta _{\mathop {\mathrm {post}}} := S \mu _{\mathop {\mathrm {post}}}. \end{aligned}$$

By definition, $\delta _{\mathop {\mathrm {post}}}$ then satisfies $\mathrm{Var}_{\eta } \left( \bar{\delta }_{\mathop {\mathrm {post}}} \right) _{ii} = 1$.

The MSE matrix of $\delta _{\mathop {\mathrm {post}}}$ is given by

36 $$\begin{aligned} \mathrm{MSE}_{\tilde{y}}(\delta _{\mathop {\mathrm {post}}}, \eta ) = S \Sigma _{\mathop {\mathrm {post}}} M \Sigma _{\mathop {\mathrm {post}}} S + (S \Sigma _{\mathop {\mathrm {post}}} M - I) \eta \eta ^{\mathop {\mathrm {T}}} (S \Sigma _{\mathop {\mathrm {post}}} M - I)^{\mathop {\mathrm {T}}}, \end{aligned}$$

which is now free of any bias caused solely by scale differences. Since $\eta$ is multivariate normally distributed with covariance matrix $\sigma _\eta$, the matrix $\eta \eta ^{\mathop {\mathrm {T}}}$ is Wishart distributed with one degrees of freedom (Srivastava, 2003):

37 $$\begin{aligned} \eta \eta ^{\mathop {\mathrm {T}}} \sim \mathrm{Wishart}(\Sigma _\eta , 1) \end{aligned}$$

Because of $1 = \nu \le T - 1$, this Wishart distribution is singular (Srivastava, 2003), but its mean still exists and is equal to $\Sigma _\eta$ as in the non-singular case. It follows that

38 $$\begin{aligned} \mathbb {E}_{\eta } \left( \mathrm{MSE}_{\tilde{y}}(\delta _{\mathop {\mathrm {post}}}, \eta ) \right) = S \Sigma _{\mathop {\mathrm {post}}} M \Sigma _{\mathop {\mathrm {post}}} S + (S \Sigma _{\mathop {\mathrm {post}}} M - I) \Sigma _\eta (S \Sigma _{\mathop {\mathrm {post}}} M - I)^{\mathop {\mathrm {T}}}. \end{aligned}$$

For the $i\mathrm{th}$ trait, we can obtain an expected RMSE marginalized over $\eta$ via

39 $$\begin{aligned} \overline{\mathrm{RMSE}}(\delta _{\mathrm{post,i}}, \eta _i) := \sqrt{\mathbb {E}_{\eta } \left( \mathrm{MSE}_{\tilde{y}}(\delta _{\mathop {\mathrm {post}}}, \eta ) \right) _{ii}}. \end{aligned}$$

I have deliberately taken the expectation before the square-root so that the expression is analytical, in line with common approaches to express average square roots of variance-like terms. The formulas for $\mathbb {E}_{\eta } \left( \mathrm{MSE}_{\tilde{y}}(\mu _{\mathop {\mathrm {post}}}, \eta ) \right)$ and $\overline{\mathrm{RMSE}}(\mu _{\mathrm{post,i}}, \eta _i)$ of the unscaled posterior means $\mu _{\mathop {\mathrm {post}}}$ can simply be obtained from Eqs. (38) and (39), by dropping the scaling matrix $S$.

As the test information increases, the RMSE decreases. Further, as the test information approaches infinity (i.e., $\det (M) \Rightarrow \infty$), we have $\Sigma _{\mathop {\mathrm {post}}} \Rightarrow M^{-1}$ and $S \Rightarrow I$ provided that $M$ is invertible (i.e., the model is identified). Hence, we get

40 $$\begin{aligned} \mathbb {E}_{\eta } \left( \mathrm{MSE}_{\tilde{y}}(\delta _{\mathop {\mathrm {post}}}, \eta ) \right) \Rightarrow I M^{-1} M M^{-1} I + (I M^{-1} M - I) \Sigma _\eta (I M^{-1} M - I)^{\mathop {\mathrm {T}}} = M^{-1} \Rightarrow 0\qquad \end{aligned}$$

such that also $\overline{\mathrm{RMSE}}(\delta _{\mathrm{post,i}}, \eta _i) \Rightarrow 0$, as it should be.

As a second important metric of predictive accuracy, I consider the *reliability*, that is, the proportion of variance in $\mu _{\mathop {\mathrm {post}}}$ explained by the true values $\eta$, or equivalently, the squared correlation between $\mu _{\mathop {\mathrm {post}}}$ and $\eta$ (Brown & Maydeu-Olivares, 2011). In contrast to the RMSE, the reliability requires variation in $\eta$ by definition and so there are no reliability coefficients of individual $\eta$ realizations. The estimates $\mu _{\mathop {\mathrm {post}}}$ and $\delta _{\mathop {\mathrm {post}}}$ of $\eta$ only vary in their overall scale, which implies their reliabilities to be the same. Accordingly, it is sufficient to derive the reliability for $\mu _{\mathop {\mathrm {post}}}$. For this purpose, we first compute the cross-covariance matrix of $\mu _{\mathop {\mathrm {post}}}$ and $\eta$ over the joint distribution $p(\tilde{y}, \eta )$ implied by Eqs. (20) and (30) as

41 $$\begin{aligned} \mathrm{Cov}_{\tilde{y},\eta }(\mu _{\mathop {\mathrm {post}}}, \eta )&= \mathrm{Cov}_{\tilde{y},\eta }(\bar{\mu }_{\mathop {\mathrm {post}}} + \varepsilon _{\mathop {\mathrm {post}}}, \eta ) \nonumber \\&= \mathrm{Cov}_{\eta }(\bar{\mu }_{\mathop {\mathrm {post}}}, \eta ) = \mathbb {E}_{\eta }(\Sigma _{\mathop {\mathrm {post}}} M \eta \eta ^{\mathop {\mathrm {T}}}) = \Sigma _{\mathop {\mathrm {post}}} M \Sigma _{\eta }, \end{aligned}$$

and the covariance matrix of $\mu _{\mathop {\mathrm {post}}}$ as

42 $$\begin{aligned} \mathrm{Var}_{\tilde{y},\eta }(\mu _{\mathop {\mathrm {post}}})&= \mathrm{Var}_{\tilde{y},\eta }(\bar{\mu }_{\mathop {\mathrm {post}}} + \varepsilon _{\mathop {\mathrm {post}}}) = \mathrm{Var}_{\eta }(\bar{\mu }_{\mathop {\mathrm {post}}}) + \mathrm{Var}_{\tilde{y}} (\varepsilon _{\mathop {\mathrm {post}}}) \nonumber \\&=\Sigma _{\mathop {\mathrm {post}}} M \Sigma _{\eta } M \Sigma _{\mathop {\mathrm {post}}} + \Sigma _{\mathop {\mathrm {post}}} M \Sigma _{\mathop {\mathrm {post}}} = \Sigma _{\mathop {\mathrm {post}}} M (\Sigma _{\eta } M + I) \Sigma _{\mathop {\mathrm {post}}}, \end{aligned}$$

where $\varepsilon _{\mathop {\mathrm {post}}} \sim \mathrm{multinormal}(0, \Sigma _{\mathop {\mathrm {post}}})$ is an error term uncorrelated with both $\bar{\mu }_{\mathop {\mathrm {post}}}$ and $\eta$ that describes the difference between $\mu _{\mathop {\mathrm {post}}}$ and its data expectation $\bar{\mu }_{\mathop {\mathrm {post}}}$. Since ${\Sigma _{\eta }}_{ii} = 1$ by definition, the reliability for the $i\mathrm{th}$ trait integrated over parameters $\eta$ and data $\tilde{y}$ is then given by

43 $$\begin{aligned} \mathrm{Rel}(\mu _{{\mathop {\mathrm {post}}},i}) = \mathrm{Cor}_{\tilde{y},\eta }(\mu _{{\mathop {\mathrm {post}}}, i}, \eta _i)^2 = \frac{\mathrm{Cov}_{\tilde{y},\eta }(\mu _{\mathop {\mathrm {post}}}, \eta )_{ii}^2}{\mathrm{Var}_{\tilde{y},\eta }(\mu _{\mathop {\mathrm {post}}})_{ii} {\Sigma _{\eta }}_{ii}} = \frac{\mathrm{Cov}_{\tilde{y},\eta }(\mu _{\mathop {\mathrm {post}}}, \eta )_{ii}^2}{\mathrm{Var}_{\tilde{y},\eta }(\mu _{\mathop {\mathrm {post}}})_{ii}}. \end{aligned}$$

In the special case of the prior resembling the sampling distributions, that is $\Sigma _{\mathop {\mathrm {prior}}} = \Sigma _\eta$, the covariance matrix of $\mu _{\mathop {\mathrm {post}}}$ simplifies to

44 $$\begin{aligned} \mathrm{Var}_{\tilde{y},\eta }(\mu _{\mathop {\mathrm {post}}})= & {} \Sigma _{\mathop {\mathrm {post}}} M \Sigma _{\eta } (M + \Sigma _{\eta }^{-1}) \Sigma _{\mathop {\mathrm {post}}} = \Sigma _{\mathop {\mathrm {post}}} M \Sigma _{\eta } \Sigma _{\mathop {\mathrm {post}}}^{-1} \Sigma _{\mathop {\mathrm {post}}} = \Sigma _{\mathop {\mathrm {post}}} M \Sigma _{\eta }\nonumber \\= & {} \mathrm{Cov}_{\tilde{y},\eta }(\mu _{\mathop {\mathrm {post}}}, \eta ) \end{aligned}$$

so that the reliability simplifies to

45 $$\begin{aligned} \mathrm{Rel}(\mu _{{\mathop {\mathrm {post}}},i}) = \frac{\mathrm{Cov}_{\tilde{y},\eta }(\mu _{\mathop {\mathrm {post}}}, \eta )_{ii}^2}{\mathrm{Cov}_{\tilde{y},\eta }(\mu _{\mathop {\mathrm {post}}}, \eta )_{ii}} = \mathrm{Cov}_{\tilde{y},\eta }(\mu _{\mathop {\mathrm {post}}}, \eta )_{ii}. \end{aligned}$$

As the test information increases, the reliability increases. Further, as the test information approaches infinity, we get

46 $$\begin{aligned} \mathrm{Cov}_{\tilde{y},\eta }(\mu _{\mathop {\mathrm {post}}}, \eta )&\Rightarrow M^{-1} M \Sigma _{\eta } = \Sigma _{\eta }, \end{aligned}$$

47 $$\begin{aligned} \mathrm{Var}_{\tilde{y},\eta }(\mu _{\mathop {\mathrm {post}}})&\Rightarrow M^{-1} M (\Sigma _{\eta } M + I) M^{-1} = \Sigma _{\eta } + M^{-1} \Rightarrow \Sigma _{\eta } , \end{aligned}$$

such that $\mathrm{Rel}(\mu _{{\mathop {\mathrm {post}}},i}) \Rightarrow 1$, independently of whether or not $\Sigma _{\mathop {\mathrm {prior}}} = \Sigma _\eta$.

We now can also ask how we should design our tests such that they maximize reliability or minimize (expected) RMSE of the person parameter estimates. These are questions that can, in the present context, be answered by means of Bayesian optimal design (Bürkner et al., 2019b; Chaloner & Verdinelli, 1995), and I provide a thorough discussion and results on this topic in Online Supplement B.

## Numerical Experiments

In Sect. 3, I have derived analytic upper information bounds for TIRT models. Based on these analytic results, I will perform numerical experiments to gain a better understanding of the relative influence of several factors related to test design on the obtainable estimation accuracy. These experiments build on and extend existing simulation studies performed for binary TIRT models (Brown & Maydeu-Olivares, 2011; Bürkner et al., 2019b; Schulte et al., 2020).

### Simulation Design

To study the behavior of person parameter accuracy more thoroughly, I vary the following factors in a fully crossed manner:

- The number of traits $T = 2, 3, 5, 10, 20, 30$ representing the full range of how many traits one might want to measure in practice.
- The total number of item pairs $B = 30, 90, 270$ ranging from very short to very long tests. The number of item pairs per trait equals $B_T = 2 B / T$ such that, for constant $B$, tests with higher number of traits have fewer item pairs per trait. This stands in contrast to previous simulation studies where, if at all, $B_T$ rather than $B$ was varied (see Online Supplement D for additional experiments with varying $B_T$).
- The mean standardized factor loading $\bar{\lambda } = 0.5, 0.65, 0.8$ ranging from medium to high values.
- The difference between the two-factor loadings of an item pair $\lambda _{\Delta } = 0.1, 0.2, 0.3$ ranging from small to high factor loading differences.
- The design type: Either a mixed keyed design (half equally and half unequally keyed pairs) denoted as (+/-) or a fully equally keyed design denoted as (+).
- The sampling correlation matrix $\Sigma _\eta$: Either diagonal or one of two conditions inspired by the NEO-PI-R (Costa & McCrae, 1992; Ostendorf & Angleitner, 2004) described in the following. For $T \le 5$ traits, take a random subset of length $T$ from the correlation matrix of the Big Five scores measured by the NEO-PI-R. For $T \ge 10$, take a random subset of length $T$ from the correlation matrix of the 30 Big Five sub-scores using the approach of Schulte et al. (2020). These correlation matrices, denoted as NEO(+/-), contain a mix of negatively, positively, and uncorrelated traits. Alternatively, create another NEO-PI-R correlation matrix, denoted as NEO(+), by inverting neuroticism into emotional stability which results in all ($T \le 5$) or most ($T \ge 10$) correlations to be non-negative (and select a subset of traits as before).
- The prior correlation matrix $\Sigma _{\mathop {\mathrm {prior}}}$: Either diagonal or equal to $\Sigma _\eta$.

Experimental conditions are defined by fully crossing the above factors. For each of the conditions, $S = 10$ simulation trials were run. In each trial, a test design meeting the criteria of the condition was obtained using the Thurstonian IRT package (Bürkner, 2019). Analytical (expected) RMSE and reliability scores were computed on that basis (see Sect. 3.3). Since the RMSE can also be computed on a per-person basis and hence may vary across people, $\eta$ parameter values were drawn from sampling distribution (30) for $J = 50$ people and individual analytic RMSE scores were obtained. In practice, multiple hundred people would be required to accurately estimate item parameters of Thurstonian IRT models (e.g., Bürkner et al., 2019b). However, as we assume item parameters to be known, the number of people has no influence on the accuracy of person parameter estimates. Given that for each person we estimate multiple traits, so that the total number of estimated person parameters is in fact equal to $J T$ per trial, $J = 50$ per trial is sufficient to show relevant RMSE variations across conditions and $\eta$ parameter values.

### Results

**Fig. 5:**
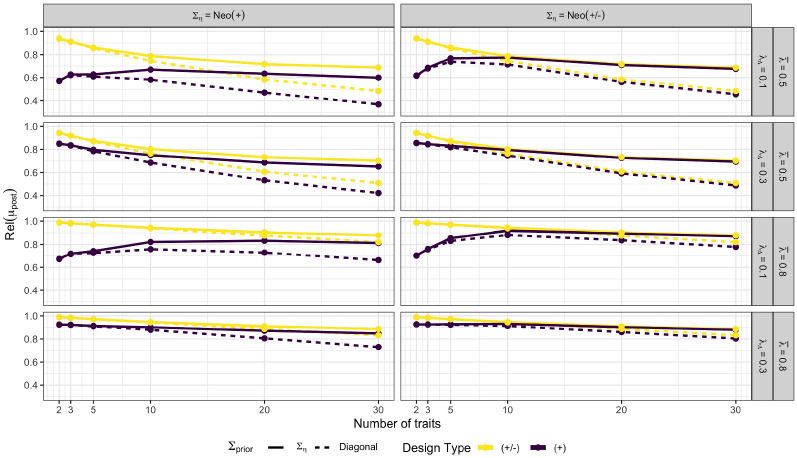
Expected reliability for $B = 90$ number of item pairs as a function of the number of traits $T$. Abbreviations: $\bar{\lambda }$ = mean factor loading; $\lambda _{\Delta }$ = factor loading difference; $\Sigma _{\eta }$ = true sampling correlation matrix; $\Sigma _{\mathop {\mathrm {prior}}}$ = prior correlation matrix. See Sect. 4.1 for more details on the simulation design and abbreviations.

Below, I present selected results of a subset of conditions from which all major conclusions regarding the influence of the above factors can be drawn. Additional results are provided in Online Supplement C and a complete overview is available on OSF (https://osf.io/2g76w/). Obtained reliability scores for $B = 90$ item pairs are illustrated in Figure 5. As expected based on the analytical findings above as well as existing simulative evidence for binary TIRT models (Brown & Maydeu-Olivares, 2011; Bürkner et al., 2019b; Schulte et al., 2020), mixed keyed designs imply a different reliability pattern and uniformly higher reliability values than equally keyed designs especially so for smaller number of traits ($T \le 10$).

For mixed keyed designs and high mean factor loadings ($\bar{\lambda } = 0.8$), reliabilities are always in a satisfactory ($\mathrm{Rel} > 0.8$) to excellent ($\mathrm{Rel} > 0.9$) range independent of all other factors varied in the numerical experiments. In contrast, for low mean factor loadings ($\bar{\lambda } = 0.5$), reliabilities are satisfactory or better only for smaller number of traits ($T \le 10$). In general, reliabilities are declining in all mixed keyed conditions as the number of traits $T$ increases, although this decline is much more noticeable for lower mean factor loadings. The factor loading difference $\lambda _{\Delta }$ plays no role for mixed keyed designs, as unequally keyed pairs already inform the within-person trait mean well enough (see also Sect. 3.1).

For equally keyed designs, reliabilities are most influenced by the number of traits $T$ and the factor loading difference $\lambda _{\Delta }$. For high factor loading differences $(\lambda _{\Delta } = 0.3)$, equally keyed designs provide only slightly worse reliabilities than the corresponding mixed keyed designs, with reliabilities decreasing slightly with increasing number of traits $T$. Especially when coupled with high mean factor loadings $(\bar{\lambda } = 0.8)$, satisfactory to excellent reliabilities can be achieved across the whole range of $T$. In contrast, for small factor loading differences $(\lambda _{\Delta } = 0.1)$, reliabilities tend to be unsatisfactory especially for smaller number of traits $(T \le 5)$. They only reach a satisfactory level for high mean factor loadings $(\bar{\lambda } = 0.8)$ as the number of traits increases further. In general, the reliabilities of equally keyed designs tend to converge to the corresponding reliabilities of mixed keyed designs as $T$ increases, but convergence is not fully achieved in all conditions for the investigated $T \le 30$ traits.

The influence of the sampling correlation matrix $\Sigma _\eta$ is noticeable only for equally keyed designs, where $\Sigma _\eta = \mathrm{Neo(+/-)}$ provides uniformly higher reliabilities than $\Sigma _\eta = \mathrm{Neo(+)}$, especially for lower number of traits $(T \le 5)$ and small factor loadings differences $(\lambda _{\Delta } = 0.1)$. We will explain this finding in Sect. 4.3. Using a misspecified prior (diagonal $\Sigma _{\mathop {\mathrm {prior}}}$, while $\Sigma _\eta$ is one of the NEO correlation matrices), is only problematic for a higher number of traits $(T \ge 10)$, but may there be quite detrimental to the achievable reliabilities especially for small mean factor loadings $(\bar{\lambda } = 0.5)$. Finally, using a higher total number of item pairs $B$ implies uniformly higher reliabilities (compare Fig. 5 to the results provided in Online Supplement C).

**Fig. 6:**
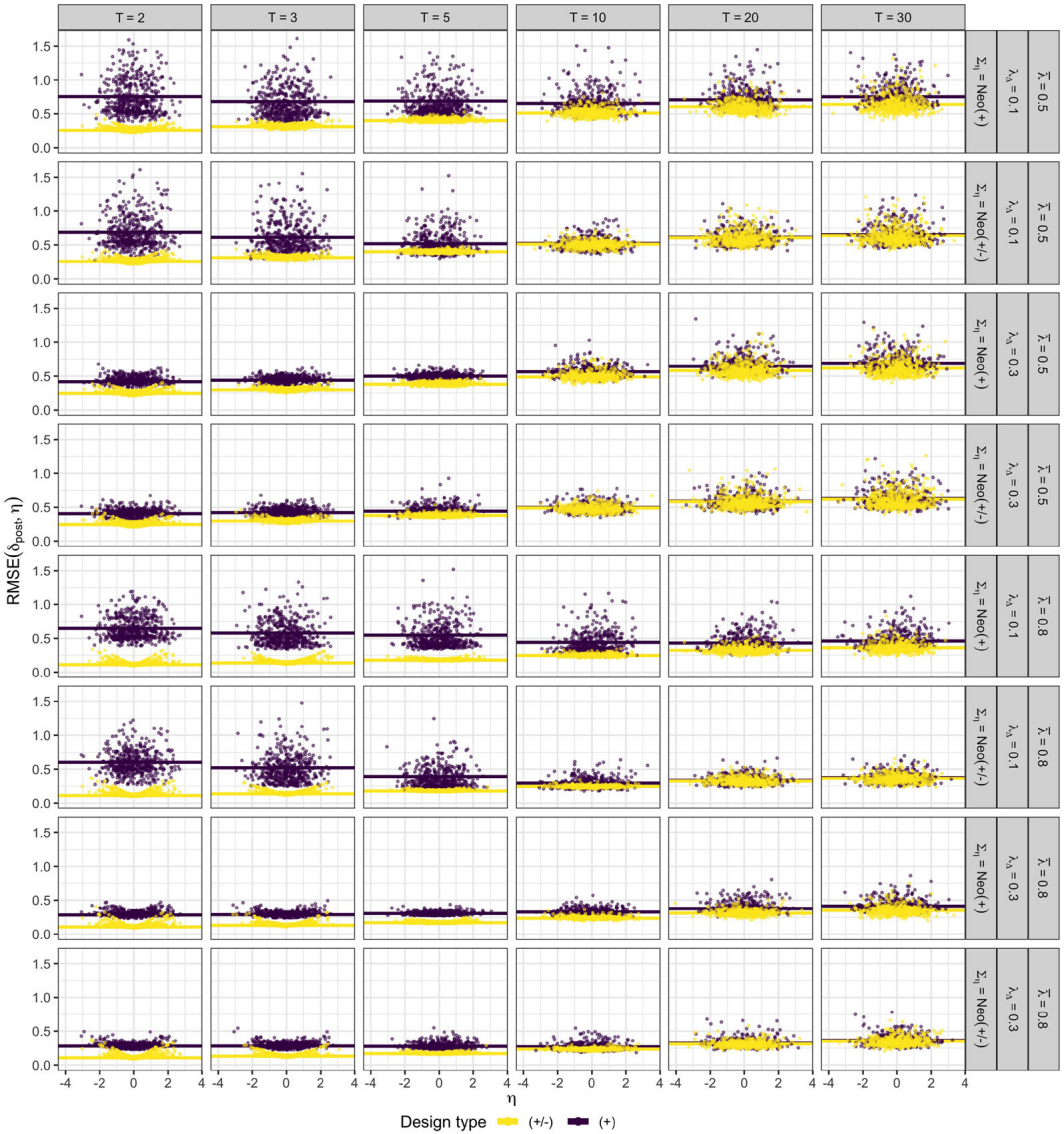
Person-trait-specific RMSEs (dots) for $B = 90$ number of item pairs as a function of the true trait scores $\eta$. Expected RMSEs are shown as horizontal lines. Abbreviations: $T$ = number of traits; $\bar{\lambda }$ = mean factor loading; $\lambda _{\Delta }$ = factor loading difference; $\Sigma _{\eta }$ = true sampling correlation matrix. See Sect. 4.1 for more details on the simulation design and abbreviations.

For the same selected conditions, distributions of obtained RMSE values are illustrated in Figure 6. Qualitatively, results from reliabilities and RMSEs paint a similar picture only that the latter is more nuanced since the RMSE can be computed on a per-person basis, while the reliability is, by definition, an expectation over people. For mixed keyed designs and smaller number of traits, individual RMSE values are almost constant across the whole range of $\eta$ values within each condition. In contrast, for equally keyed designs, RMSE values vary considerably across $\eta$ values within each conditions and are noticeably bigger on average than for the corresponding mixed keyed design. For higher number of traits ($T = 20, 30$ in particular), differences in RMSE distributions between equally and mixed keyed designs become smaller or even diminish completely for some conditions. This extends the results of Schulte et al. (2020) who investigated RMSEs obtained from binary TIRT models for varying number of traits and found that, for a very high number of traits, average RMSEs obtained from equally and mixed keyed designs are highly similar. Similar to the results obtained for the reliabilities, a misspecified prior (diagonal $\Sigma _{\mathop {\mathrm {prior}}}$, while $\Sigma _\eta$ is one of the NEO correlation matrices) increases the expected RMSE primarily for higher number of traits ($T \ge 10$; see Online Supplement C). However, at the same time, there is a striking variation across trait scores with respect to how much a misspecified prior changes the RMSE of the trait score estimates.

**Fig. 7:**
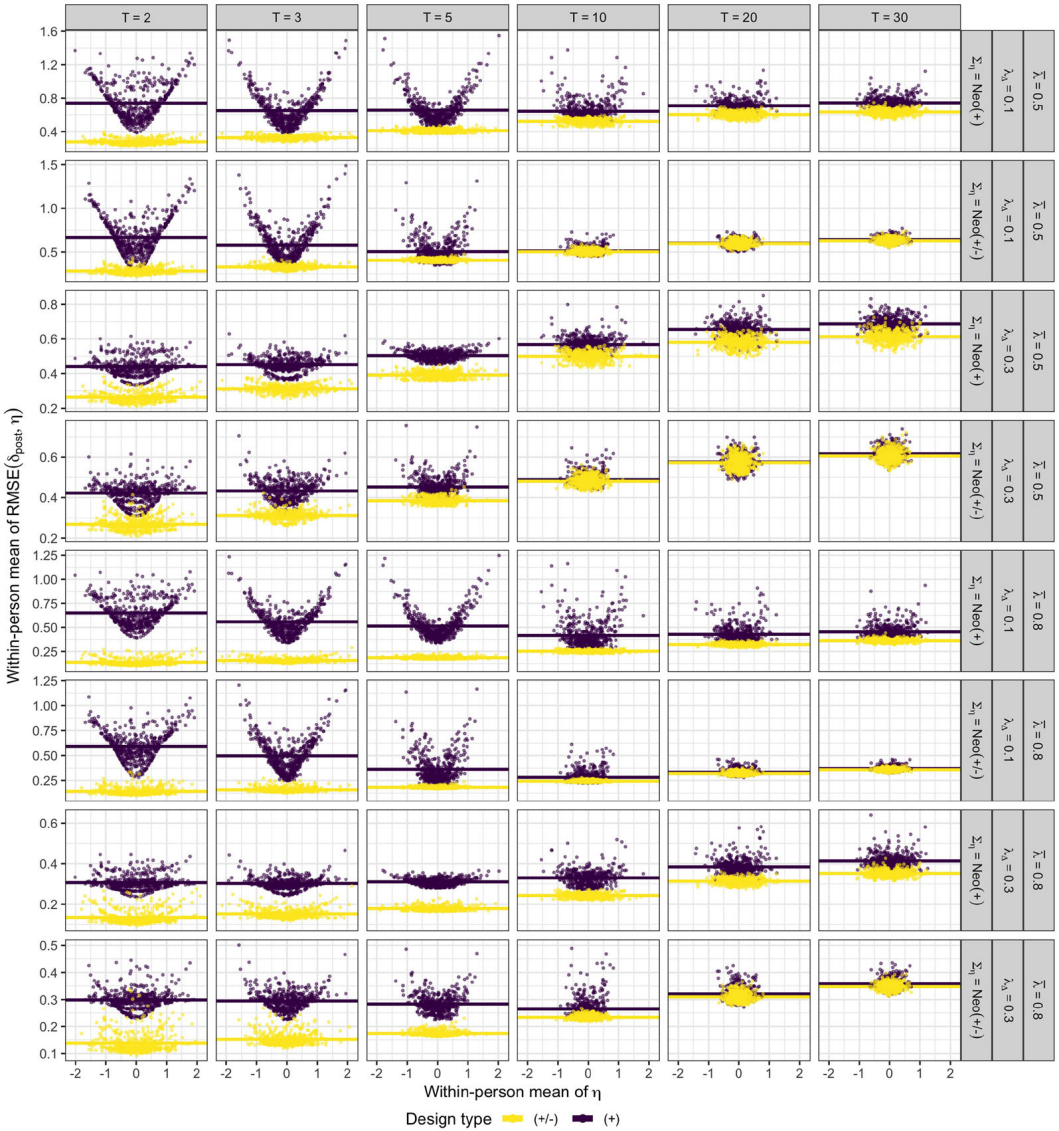
Person-specific RMSEs (dots; averaged over traits) for $B = 90$ number of item pairs as a function of the true within-person trait score mean $\bar{\eta }$. Expected RMSEs are shown as horizontal lines. Abbreviations: $T$ = number of traits; $\bar{\lambda }$ = mean factor loading; $\lambda _{\Delta }$ = factor loading difference; $\Sigma _{\eta }$ = true sampling correlation matrix. See Sect. 4.1 for more details on the simulation design and abbreviations.

As illustrated in Figure 7, the within-person mean $\bar{\eta }$ over trait scores can explain some of the within-condition variation of RMSE scores in the equally keyed design conditions for small number of traits ($T \le 5$) and small factor loading differences $(\lambda _{\Delta } = 0.1)$. In those cases, RMSE scores are particularly high if a person has a very negative or very positive mean, corresponding to overall low and high trait scores. This is the result of partial ipsativity of trait estimates induced by equally keyed designs which, as demonstrated here, is still present even in the limiting case of the latent linear model and thus cannot be eliminated through the application of ordinal TIRT models. Explained in more detail, trait scores of people with low variation between traits are estimated closer to zero, thus inducing strong biases and thus high RMSE for people with low or high average trait scores. This pattern is much less visible for higher number of traits ($T \ge 10$), a finding which I investigate and explain further in Sect. 4.3. Figure 7 also illustrates that the within-person RMSE mean may vary strongly across people within the same condition, even when their $\bar{\eta }$ values are similar. The variation in mean RMSE scores may vary by a factor of $2$ or even $3$ between people with the lowest and highest mean RMSE within condition. Notably, this variation can neither be explained by variation across simulation trials within each condition nor by shrinkage of parameter estimates induced by the prior (see Online Supplement C). Together, the results demonstrate that differences in between traits of the same person can be estimated well as long as equally keyed item pairs are present confirming theoretical results (see also Brown & Maydeu-Olivares, 2011).

### Increasing Test Information by Measuring More Traits

In the above numerical experiments, I systematically varied the total number of item pairs $B$ rather than the number of item pairs per trait $B_T$. Comparing different number of traits for an arbitrary, but fixed $B$ (my approach above) implies fixing the overall test length. In contrast, comparing different number of traits for an arbitrary, but fixed $B_T$ implies the test to become longer for a higher number of traits, up to a point where the test length becomes impractically high for most applications. In a simulation study for binary TIRT models, Schulte et al. (2020) used the approach of systematically varying $B_T$ and found that higher number of traits imply strong increases in estimation accuracy, especially for equally keyed designs. Results of additional numerical experiments mirroring the simulation design from Sect. 4.1, apart from systematically varying $B_T$ rather than $B$, confirm that this behavior can be found in latent linear TIRT models as well (see Online Supplement D).

A comprehensive explanation for the apparent benefit of a higher number of traits when using equally keyed designs has been lacking so far. Baron (1996) identified one mechanism related to $\Sigma _\eta$ in that highly skewed true trait patterns, where most traits of an individual are either very high or low, become less likely as the number of traits increases (see Online Supplement E for a more detailed discussion). Below, I discuss two additional mechanisms, related to the Fisher information matrix $M$ and the prior correlation matrix $\Sigma _{\mathop {\mathrm {prior}}}$, respectively. Together, each of the three essential matrices ($M, \Sigma _\eta , \Sigma _{\mathop {\mathrm {prior}}}$) has a corresponding mechanism by which higher number of traits benefits estimation accuracy.

**Fig. 8:**
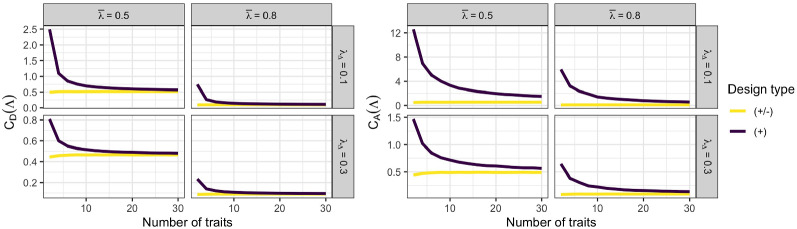
D-optimality criterion (left-hand side) and A-optimality criterion (right-hand side) for fixed number of item pairs per trait $B_T$ as a function of the number of traits $T$. Lower values are better. Abbreviations: $\bar{\lambda }$ = mean factor loading; $\lambda _{\Delta }$ = factor loading difference; $(+/-)$ = mixed keyed design; $(+)$ = equally keyed design.

**Fig. 9:**
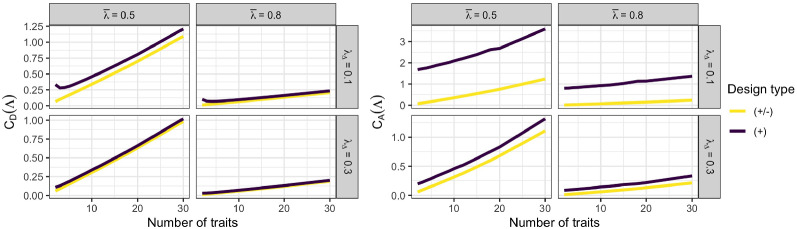
D-optimality criterion (left-hand side) and A-optimality criterion (right-hand side) for fixed total number of item pairs $B$ as a function of the number of traits $T$. Lower values are better. Abbreviations: $\bar{\lambda }$ = mean factor loading; $\lambda _{\Delta }$ = factor loading difference; $(+/-)$ = mixed keyed design; $(+)$ = equally keyed design.

The mechanism related to $M$ is the increase in per-trait information in equally keyed designs through measuring more traits while holding $B_T$ constant. Figure 8 shows the scaled D- and A-optimality criteria (Eqs. (16) and (19)) for varying number of traits $T$. Due to the scaling by $T$, these criteria are comparable across varying number of traits. For equally keyed designs, it is clearly visible that the per-trait information improves consistently in all investigated conditions as more traits are measured. The information improvement is particularly strong for smaller $T$ and then gradually flattens out toward a lower asymptote that depends on the mean factor loading $\bar{\lambda }$ and on $B_T$. In contrast to equally keyed designs, the per-trait information for mixed keyed designs is constant across traits and equals the asymptote that equally keyed designs are only approaching for higher number of traits. When holding the total number of pairs $B$ constant, instead of the number of pairs per trait $B_T$, a higher number of traits no longer implies an increase in per-trait information (smaller criterion values) but rather an almost linear *decrease* in information (see Figure 9), with a slope depending on $\bar{\lambda }$, $\lambda _{\Delta }$, and $B$.

The mechanism related to $\Sigma _{\mathop {\mathrm {prior}}}$ is the increase in variance of each trait that can be explained by means of all other traits. If prior correlations are nonzero, different trait estimates will inform each other through the prior and thus be pushed along the axes implied by the correlation structure. Clearly, the influence of the prior is only helpful if $\Sigma _{\mathop {\mathrm {prior}}}$ contains some *true* prior information, that is, relates closely enough to the sampling correlation matrix $\Sigma _{\eta }$. If that is the case, as one trait becomes estimated more accurately, so will be all other traits correlated with the former.

Of course, if all traits are mutually uncorrelated, the variance explained would still be zero no matter the number of traits. However, in practice, the more traits are measured, the higher their (absolute) correlations will be in expectation. This is simply due to the fact that the amount of mutually independent personality traits, or other constructs one aims to measure via comparative judgments, is naturally limited. Not even the Big-Five personality scores are completely uncorrelated with each other. For example, the main Big-Five scores measured by NEO-PI-R have an average absolute correlation of $|r| =$ 0.19, while the corresponding 30 Big-Five sub-scores (six per main Big-Five dimension) have an average absolute correlation of $|r| =$ 0.26. Even if the average absolute correlation remained constant for increasing number of traits, the number of traits themselves will lead to an increase in variance explained per trait as more other traits can be used for prediction. For the main Big-Five NEO-PI-R scores, the average percentage of variance explained per trait by means of all other traits is $R^2 =$ 0.23, while for the corresponding 30 sub-scores, one finds $R^2 =$ 0.81. This large percentage of variance explained in the latter case is mostly but not exclusively driven by other sub-scores belonging to the same main dimension.

These implications of the between-parameter shrinkage property of joint normal priors not only holds when $\Sigma _{\mathop {\mathrm {prior}}}$ is known, as assumed in this paper, but also when it is estimated from the data (Gelman & Hill, 2006). Of course, $\Sigma _{\mathop {\mathrm {prior}}}$ has to be estimated without substantial bias in order to be informative for the trait scores themselves. As shown by previous simulation studies, estimates of $\Sigma _{\mathop {\mathrm {prior}}}$ may be substantially biased for smaller number of traits ($T \le 5$) but become much less biased or even unbiased as the number of traits increases further up to $T = 30$ (Bürkner et al., 2019b; Schulte et al., 2020). This provides some sort of additional sub-mechanism by which the prior improves estimation accuracy when measuring a higher number of traits.

## Discussion

The presented research is driven by the main goal of obtaining accurate and efficient estimates of people’s latent traits using models of comparative judgments, while keeping an eye specifically on the applicability under high-stakes situations. Any procedure applied in a high-stakes context needs to be faking resistant—or at least have a realistic potential to be faking resistant—while at the same time yielding trait score estimates that are accurate enough for individual-level diagnostic decisions. Toward this goal, two paths have been identified. First, develop unequally keyed item pairs where both items have roughly the same social desirability and can thus be reasonably applied in high-stakes situations. Second, carefully design tests consisting only of equally keyed item pairs so that they alone are sufficient to ensure satisfactory estimation accuracy. This paper focuses on the second path approaching it mainly from a statistical point of view.

Progress was made from several perspectives all related to answering the main question. Below, I will connect these perspectives into a single, coherent picture based on which practical recommendations can be given. Walking along the second path is full of obstacles that we have to get around one by one. As a reminder, unless explicitly stated otherwise, I consider higher trait scores representing the more desirable (better) end of the scale. Because the choice of trait direction is essentially arbitrary, it does not pose any actual restriction, but makes thinking about the discussed problems easier.

### Obtaining Accurate Trait Estimates from Equally Keyed Item Pairs

For the purpose of individual-level diagnostic decisions, using highly accurate trait score estimates is not only sensible but ethically required. One way to increase the information from comparative judgments is to employ ordinal rather than binary response scales (see Section 2 as well as Brown & Maydeu-Olivares, 2018; Schulte et al., 2021). In this context, I have established that the information factor (defined in Eq. (8)) can be used as an intuitive metric to quantify the Fisher information gained in comparison with binary measurement models, or to quantify the Fisher information lost in comparison with latent linear models. When using two response categories per item pair (i.e., binary decisions), the average expected (Fisher) information is only about 13% of the maximal achievable information under conditions not unrealistic for TIRT models with item pairs matched for social desirability. In comparison, when using as few as five response categories, the average expected information is already about 65% of the maximum and even increases to about 85% when using ten response categories. Even if we consider the cognitive complexity of the response process and treat the ranking of a triplet (two decisions leading to three binary responses) as equivalently complex to a single ordinal response, the latter still contains substantially more information (39% vs. 65% or 85% of the maximum, on average).

Upon studying the information factor in detail, we had seen that its distribution across individual responses becomes narrower and is substantially higher than zero in a much wider range of the latent scale. This leads me to conjecture that, as we increase the number of response categories, the ordinal model may also become more robust to moderate amounts of variation in social desirability. However, this needs to be verified in empirical studies with faking instructions. In any case, such robustness will have its limits: If one of the two items compared in an item pair is much more socially desirable than the other, and if people react to the social desirability, this will imply a strong shift in the ordinal thresholds toward the end of response scale belonging to the less desirable item (see also Schulte et al., 2021). This in turn pushes the latent responses more into the tails, beyond the range where the convergence of the information factor to its maximum is still reasonable fast. Thus, social desirability matching continuous to be mandatory also in ordinal models of comparative judgments, but we may get away with a little less perfect matching compared to the binary case.

Although ordinal models certainly help to increase information on individuals’ trait scores, more measures need to be taken to obtain trait estimates applicable for individual-level diagnostic decisions in high-stakes situations. Again also for ethical reasons, trait estimates of all individuals should have roughly the same accuracy, for example, as quantified via their RMSE. Thus, not only large *average* RMSEs (or small reliabilities) are problematic in practice, but also large RMSE *variations* across traits and individuals. The numerical experiments performed in this paper have demonstrated that, when only using equally keyed item pairs, trait score estimates of individuals with particularly low or high (within-person) *average* scores tend to be comparably less accurate. In personnel selection, or other high-stakes situations, individuals with low or high average scores are of primary interest. Accordingly, it is particularly problematic if trait scores of specifically those individuals cannot be estimated with sufficient accuracy.

From a statistical perspective, the reason for the above problem is that Fisher information about within-person average trait scores is only provided through unequally keyed item pairs or factor loading differences. If we restrict ourselves to equally keyed item pairs only, all we are left with are the factor loading differences. Of course, those differences cannot become arbitrarily large because standardized factor loadings very close to one are hard to achieve in practice (e.g., Costa & McCrae, 1992). Also, the closer factor loadings get to zero, the less information the corresponding items provide. This induces a natural trade-off between high mean factor loadings and high factor loading differences of item pairs, a trade-off I have approached in this paper in a principled manner by means of optimal design theory.

Different design criteria find different trade-offs between high factor loading means and differences to be optimal. Under several of the considered (frequentist and Bayesian) optimality criteria, very high of even maximal factor loading differences are optimal. That is, one of the two items in a pair should have a maximal factor loading—whatever we consider to be maximally achievable in a given practical setting—while the other should have a very small, or even zero, factor loading. Using items with all zero factor loadings may be statistically optimal but comes with two practical problems. First, one has to design items that do not load on any of the desirable traits of interest but still have comparable social desirability with items that do load strongly on such a trait. A weaker version of this problem is likely to hold also for positive but comparably small factor loadings. I speculate that the more factor loadings differ from each other, the harder it becomes to match the social desirability of the corresponding items, but this needs to be investigated empirically in future research. Second, individuals will almost surely vary in whatever non-modeled traits determine the responses to the items that have only zero (or very small) factor loadings on the modeled traits. As a result, the corresponding variation of the non-modeled traits will be ignored. This is essentially a misspecification of the latent model structure with potentially detrimental effect on the validity of the estimated model (e.g., Hu & Bentler, 1998). As a result, very high factor loading differences pose a risk to the practical validity of the trait score estimates, and we should thus be careful to trust their statistical optimality too much.

In the numerical experiments, factor loadings differences were varied in a comparably smaller range (0.1–0.3). As expected, the bigger the factor loading differences (within the considered range), the lower RMSEs become across the board, that is, also for individuals with more extreme average trait scores. However, it is only with higher number of traits (starting between 5 and 20 traits depending on condition) that the U-shape of individual RMSE scores as a function of within-person average trait scores starts to vanish. So how do higher number of traits help with estimation accuracy in the tails even if the number of administered item pairs is held constant? Multiple mechanisms have been identified. One of these mechanisms is that that more extreme average trait scores simply become less likely the more traits are considered (Baron, 1996, also discussed in Online Supplement E). To put it another way, by measuring a large-enough number of traits, we increase the within-person variation in trait scores relative to the between-person variation; and the former kind of variation can be estimated well with only equally keyed items. Unfortunately, measuring more traits also has the drawback that, in a fixed-length test, the number of items measuring a single trait decreases, thus reducing the average by-trait information. So, again, we find a trade-off between different mechanisms, this time in the number measured traits. For small factor loading differences, this trade-off reached its optimum at around 10 traits, whereas for higher factor loading differences, the optimum is a little smaller somewhere between 5 and 10 traits. This optimum also depends on other factors influencing estimation accuracy (see below) and may very well be higher than 10 depending on these factors. Accordingly, these optimal values should be considered with care and rather taken as a rough rule of thumb.

The structure of the inter-trait correlation matrix is another factor that can have strong influence on the achievable estimation accuracy. Measuring traits with a mix of negative, positive, and zero correlations implies higher estimation accuracy than measuring traits that are mostly zero or positively correlated. The mechanism behind this finding turns out to the same one that is behind the beneficial properties of measuring more traits, that is, reducing the probability of individuals having more extreme average trait scores. Accordingly, we can get away with measuring fewer traits if some of those traits turn out to be negatively correlated. Two notes of caution: First, the above statements apply only when, for all traits, higher values represent the desirable end of the scale. Of course, we can invert some traits to artificially create negative inter-trait correlation, but this does not, in fact, change anything. We simply switch labels so that unequally keyed item pairs, between a higher-means-better trait and a lower-means-better trait, become the ones that can well be matched for social desirability, whereas equally keyed item pairs suddenly become practically problematic. Second, the correlations between traits are usually not something that it under the control of the test developer. Instead, it is determined by the traits being considered as relevant for the given high-stakes situation and the population of individuals taking the test. That is, in some situations, we may be lucky and find these negative correlations between the measured desirable traits, while in some situations, we may not. So we cannot rely on negative inter-trait correlations in general, but it will help if they occur.

### Practical Recommendations

In summary, the results of the present study suggest that achieving practically and ethically acceptable estimation accuracy for inter-individual decision making using only equally keyed item pairs is indeed possible but requires the careful consideration of several factors. For example, acceptable reliabilities ($\text {Rel > 0.8}$) can be achieved if all of the following conditions are met or exceeded: An ordinal measurement model with five response categories is applied to 90 item pairs, ten traits are measured with some of them being negatively correlated, mean factor loadings are high (0.8), and factor loading differences are medium (0.2). If we make some conditions more restrictive, for example, increase the number of response categories or the number of item pairs, other conditions may be relaxed while still retaining acceptably accurate estimates. As different application contexts of comparative judgments may each come with their own idiosyncrasies, one should understand these conditions more like rough guidelines not as definitive recommendations. If in doubt, the analyses performed in this paper can be replicated with the materials provided online (https://osf.io/2g76w/) and adjusted to the given application context.

### Limitations and Future Research

There are several limitations of the present study which should be taken into account and expanded upon in future research. First, most of the presented analytical proofs and numerical experiments focus on latent linear TIRT models that provide an upper bound of the Fisher information obtainable from ordinal (including binary) TIRT models. Thus, the obtained results only indicate the maximal achievable accuracy by means of TIRT models in a given situation, which will not be fully achieved in practice (although one may come quite close; see above). This choice was made to allow for an extensive mathematical analysis that is much harder for the ordinal models themselves, due to the nonlinearity of the ordinal response categorization. Still, important results such as the optimal designs for factor loadings turn out to also hold in the same manner for ordinal models. This underlines the practical relevance of the obtained analytical and numerical findings. What is more, extreme average trait scores that turned out to be difficult to estimate for any kind of TIRT model, can be estimated by the ordinal models almost as well as by the latent linear models. This is because the corresponding latent responses are located in the center of the latent response distribution, where information of the ordinal models approaches optimality very quickly.

Apart from the present study, existing research on ordinal TIRT models (Brown & Maydeu-Olivares, 2018; Schulte et al., 2021) has only applied them in empirical settings, whereas comprehensive simulation studies on these models are still missing. They may also help to validate the approximate results obtained in this paper. For example, we may use the average information factor to adjust the Fisher information matrix of the latent linear model in order to approximate the expected reliabilities and RMSEs obtained from ordinal models. However, until further validation, the goodness of this approximation remains unclear. Due to its already extensive scope, bespoken simulations were not performed in this paper, but provide an interesting area for future research.

Another limitation of the present study is that the item parameters were considered known instead of being estimated from the data. Again, this choice was made to enable a deeper analytical treatment of the TIRT models. Of course, in practice, item parameters will almost always be estimated, which makes a big difference with regard to the required estimation algorithms and their stability (e.g., Bürkner et al., 2019b; van der Linden & Hambleton, 2013). However, with respect to the estimation accuracy of trait scores, this choice may actually not be that relevant: In the TIRT simulation study of Schulte et al. (2020), estimation accuracy of trait scores saturated already with 300 (or more) individuals. This indicates that uncertainty in item parameters becomes irrelevant to trait score accuracy quite quickly. When applying IRT models in practice, it is common to measure several hundreds or perhaps even thousands of people. Accordingly, in practice, the assumption of known item parameters is unlikely to affect the information obtained on individuals’ trait scores to a relevant degree.

A related limitation, again motivated by the requirements of mathematical analysis, was the fixation (rather than estimation) of the inter-trait correlation matrix. What is more, a lot of the presented numerical results were obtained by fixing the correlation matrix to its true value. To investigate the robustness of these results to prior-misspecification, the same experiments were run using a diagonal prior correlation matrix, essentially assuming traits to be uncorrelated a-priori. This kind of misspecification turned out to only affect estimation accuracy noticeably for higher number of traits and even then only in situation where the test design provided comparably little information (e.g., small average factor loadings or small factor loading differences). To better put this into perspective, two things should be considered: First, the assumption of a-priori zero correlations constitutes a strong prior misspecification given that chosen true correlation matrices based on the NEO-PI-R contain a lot of highly nonzero values (Costa & McCrae, 1992). Second, estimation accuracy was found to be sufficient for individual diagnostics only under conditions where there is quite a lot of test design information; conditions were sensitivity to the prior was low anyway. In practice, the correlation matrix usually represents a hyper-parameter estimated jointly from the data of all individuals. Previous simulation studies have shown the trait correlation matrix tends to be estimated inaccurately and with substantial bias precisely in those situations where individual trait scores are estimated inaccurately as well (Brown & Maydeu-Olivares, 2011; Bürkner et al., 2019b). Conversely, if trait scores were estimated accurately, so was their correlation matrix. In summary, with regard to results of the present paper, it appears unlikely that the conditions identified as yielding sufficiently accurate trait score estimation would yield much different results if the correlation matrix was estimated from the data as part of the model fitting procedure. A more systematic investigation of this topic is desirable and could be an interesting area for future research.

Lastly, a highly important area for future research is to better understand faking behavior of individuals and faking resistance of personality tests. Comparative judgments are commonly understood as being able to reducing faking because they prevent all items from being endorsed maximally at the same time (Cao & Drasgow, 2019; Wetzel et al., 2016). Honest-faking studies have shown that TIRT models of comparative judgments can indeed decrease score inflation compared to standard rating scale models (Schulte et al., 2021; Wetzel et al., 2020; see also Cao & Drasgow, 2019). However, one of these studies (Schulte et al., 2021) not only considered score-inflation but also the correlation between honest and faking scores as a metric of faking resistance. Unexpectedly, they found that rating scales lead to higher (better) honest-faking correlations. These inconsistent findings also call for more theoretical research to properly *define* faking resistance in the first place, on which basis a better understanding of faking and faking resistance can then be obtained by further empirical studies.

## Conclusion

In this paper, I have investigated the information obtainable from comparative judgments by means of TIRT models using a combination of analytical and numerical approaches. The obtained results suggest that it is indeed possible to design personality tests that yield trait score estimates sufficiently accurate for individual-level diagnostic decisions, while having a realistic potential to prevent faking in high-stakes situations. However, reaching this goal requires the careful joint consideration of several aspects of test design, including number of response categories, number of item pairs, number of measured traits, correlations between traits, average factor loadings, and factor loading differences. While these results are encouraging and ground-breaking, they remain to be validated empirically to demonstrate that tests meeting the given requirements can indeed be constructed and successfully applied in practice in high-stakes situations. If that practical validation succeeds, this would be a major breakthrough for the fields of psychological diagnostics, differential psychology, and their areas of application.

### Supplementary Information

Below is the link to the electronic supplementary material.

Supplementary file 1 (pdf 15171 KB)

## Appendix

### Proof of Corollary 2.1

Consider the Fisher information $\mathbb {I}_\mathrm{TIRT}(\eta )$ of an ordinal TIRT model from Eq. (9) and the Fisher information $\mathbb {I}(\eta )$ of the corresponding linear normal model from Eq. (10). Choose observation *n* arbitrarily and choose the *K* inner thresholds $\tau _k$ such that $s_k = \tau _k - X_n \eta$ for some arbitrary choice of design matrix *X* with nonzero $n\mathrm{th}$ row. Then, comparing the Fisher information matrices reveals

48 $$\begin{aligned} 0 \le \sum _{k=1}^{K+1} \frac{(\phi (s_k) - \phi (s_{k-1}))^2}{\Phi (s_k) - \Phi (s_{k-1})} < 1, \end{aligned}$$

where the first inequality is because of the positive (semi-)definiteness of the Fisher information and the second inequality is due to Theorem 1.4 in Online Supplement A. The equality to 1 in the limit of infinite thresholds

49 $$\begin{aligned} \sum _{k=1}^{\infty } \frac{(\phi (s_k) - \phi (s_{k-1}))^2}{\Phi (s_k) - \Phi (s_{k-1})} = 1 \end{aligned}$$

is now an immediate consequence of Theorem 1.5 in Online Supplement A.

### Proof of Theorem 3.1

In case of standardized item parameters (which we can assume without loss of generality), we have

50 $$\begin{aligned} \varphi ^2_n = \psi _{n1}^2 + \psi _{n2}^2 = 2 - \lambda _{n1}^2 - \lambda _{n2}^2 \end{aligned}$$

as the square of the denominator occurring in the $n\mathrm{th}$ row $X_n$ of the design matrix. Let us start with the minimal non-trivial design of $T = 2$ traits and $R_{12} = 2$ comparisons between the $1\mathrm{st}$ and the $2\mathrm{nd}$ trait, so that we have a total of $N = 2$ comparisons. Then, we obtain the Fisher information as

51 $$\begin{aligned} M = X_1 X_1^{\mathop {\mathrm {T}}} + X_2 X_2^{\mathop {\mathrm {T}}} = \frac{1}{\varphi ^2_1} \left( \begin{array}{rr} \lambda _{11}^2 &{} - \lambda _{11} \lambda _{12} \\ - \lambda _{11} \lambda _{12} &{} \lambda _{21}^2 \\ \end{array}\right) + \frac{1}{\varphi ^2_2} \left( \begin{array}{rr} \lambda _{21}^2 &{} - \lambda _{21} \lambda _{22} \\ - \lambda _{21} \lambda _{22} &{} \lambda _{22}^2 \\ \end{array}\right) \end{aligned}$$

and the determinant evaluates to

52 $$\begin{aligned} \mathrm{det}(M) = \left( \frac{1}{\varphi ^2_1} \lambda _{11}^2 + \frac{1}{\varphi ^2_2} \lambda _{21}^2 \right) \left( \frac{1}{\varphi ^2_1} \lambda _{12}^2 + \frac{1}{\varphi ^2_2} \lambda _{22}^2 \right) - \left( - \frac{1}{\varphi ^2_1} \lambda _{11} \lambda _{12} - \frac{1}{\varphi ^2_2} \lambda _{21} \lambda _{22} \right) ^2. \end{aligned}$$

Clearly, the first of the two additive terms is maximized if $\lambda _{in} \in \{-\lambda _\mathrm{max}, \lambda _\mathrm{max}\}$ for all $1 \le i,n \le 2$ (note that $\phi _1^2$ and $\phi _2^2$ are also minimized by this choice), while the second additive term is minimized if

53 $$\begin{aligned} - \frac{1}{\varphi ^2_1} \lambda _{11} \lambda _{12} - \frac{1}{\varphi ^2_2} \lambda _{21} \lambda _{22} = 0 \end{aligned}$$

This equation has many solutions, one of which is given by $\lambda _{11} = \lambda _{21} = \lambda _{22} = \lambda _\mathrm{max}$ and $\lambda _{12} = -\lambda _\mathrm{max}$. But this solution also maximizes the first term which proves its optimality in case of $T = 2$ and $R_{12} = 2$.

For arbitrary $T \ge 2$ number of traits and even $R_{ij} \ge 0$ number of comparisons between traits $i \ne j$, we note that every comparison contributes information only to two traits and corresponding four elements of *M*, while all other elements remain unchanged due to additivity of the Fisher information. That is, having already included an even number of $\tilde{N} < N$ comparisons to the design, adding two more comparisons $\tilde{N}+1$ and $\tilde{N}+2$ of traits $i \ne j$, we get

54 $$\begin{aligned} (M_{\tilde{N}+2})_{ii} =&(M_{\tilde{N}})_{ii} + \frac{1}{\varphi ^2_{(\tilde{N}+1)}} \lambda _{(\tilde{N}+1)1}^2 + \frac{1}{\varphi ^2_{(\tilde{N}+2)}} \lambda _{(\tilde{N}+2)1}^2 \nonumber \\ (M_{\tilde{N}+2})_{jj} =&(M_{\tilde{N}})_{jj} + \frac{1}{\varphi ^2_{(\tilde{N}+1)}} \lambda _{(\tilde{N}+1)2}^2 + \frac{1}{\varphi ^2_{(\tilde{N}+2)}} \lambda _{(\tilde{N}+2)2}^2 \nonumber \\ (M_{\tilde{N}+2})_{ij} =&(M_{\tilde{N}})_{ij} - \frac{1}{\varphi ^2_{(\tilde{N}+1)}} \lambda _{(\tilde{N}+1)1} \lambda _{(\tilde{N}+1)2} - \frac{1}{\varphi ^2_{(\tilde{N}+2)}} \lambda _{(\tilde{N}+2)1} \lambda _{(\tilde{N}+2)2} \nonumber \\ (M_{\tilde{N}+2})_{ji} =&(M_{\tilde{N}})_{ji} - \frac{1}{\varphi ^2_{(\tilde{N}+1)}} \lambda _{(\tilde{N}+1)1} \lambda _{(\tilde{N}+1)2} - \frac{1}{\varphi ^2_{(\tilde{N}+2)}} \lambda _{(\tilde{N}+2)1} \lambda _{(\tilde{N}+2)2} \end{aligned}$$

and $(M_{\tilde{N}+2})_{kl} = (M_{\tilde{N}})_{kl}$ for all other elements, where $M_{\tilde{N}}$ and $M_{\tilde{N}+2}$ denote the Fisher information after a total of $\tilde{N}$ and $\tilde{N}+2$ comparisons, respectively.

If we plug in our solution from the $T = R_{12} = 2$ case above, that is, choose $\lambda _{(\tilde{N}+1)1} = \lambda _{(\tilde{N}+2)1} = \lambda _{(\tilde{N}+2)2} = \lambda _\mathrm{max}$ and $\lambda _{(\tilde{N}+1)2} = -\lambda _\mathrm{max}$, we see that the *ij* and *ji* increments are 0 such that $(M_{\tilde{N}+2})_{ij} = (M_{\tilde{N}})_{ij}$ and $(M_{\tilde{N}+2})_{ji} = (M_{\tilde{N}})_{ji}$, while the diagonal elements *ii* and *jj* increase maximally in the space of all admissible factor loadings. We apply this solution to *all* such sets of two item pairs, while ensuring that all traits appear in the same number of pairs in total. We denote the resulting Fisher information of this design (suggestively) as $M_\mathrm{max}$. By induction, we see that all off-diagonal elements $(M_\mathrm{max})_{ij} = 0$, and thus the determinant then simply equals

55 $$\begin{aligned} \det (M_\mathrm{max}) = \prod _{i=1}^T (M_\mathrm{max})_{ii}. \end{aligned}$$

The diagonal elements of any *M* are of the form

56 $$\begin{aligned} M_{ii} = \sum _{j \in J_i} \frac{1}{\varphi ^2_{n_j}} \lambda ^2_j, \end{aligned}$$

where $J_i$ is the index set of all items belonging to the $i\mathrm{th}$ trait and $n_j$ is the comparison with which the $j\mathrm{th}$ item belongs. Since the total number of factor loadings sums to $\sum _{i=1}^T |J_i| = 2N$, the product $\prod _{i=1}^T M_{ii}$ is maximal if each $\frac{1}{\varphi ^2_{n_j}} \lambda ^2_j$ is maximal and all $M_{ii}$ are equal (square maximization property), both of which are satisfied by $M_\mathrm{max}$.

It remains to be shown that $\det (M_\mathrm{max})$ is indeed maximal within the space of all admissible designs not just within the space of all design implying a diagonal Fisher information. For this purpose, we apply Cholesky decomposition to *M* such that $M = L L^T$ of lower triangular matrix *L*. This is always possible if *M* is positive definite; and if *M* was only positive semi-definite, we had $\det (M) = 0$ and thus a degenerate design, which is certainly not optimal. We obtain $\det (M) = \det (L) \det (L^T) = \det (L)^2$ and, since *L* is lower triangular, $\det (L) = \prod _{i=1}^T L_{ii}$. Further, as $M_\mathrm{max}$ is diagonal, we see that $L_\mathrm{max}$ is also diagonal and $(L_\mathrm{max})_{ii} = \sqrt{(M_\mathrm{max})_{ii}}$. We now only need to show that $L_{ii} \le (L_\mathrm{max})_{ii}$ for every admissible design. Applying the Cholesky-Banachiewicz algorithm to determine *L*, we see that

57 $$\begin{aligned} L_{ii} = \sqrt{M_{ii} - \sum _{j=1}^{i-1} L_{ij}^2}. \end{aligned}$$

From $\prod _{i=1}^T M_{ii} \le \prod _{i=1}^T (M_\mathrm{max})_{ii}$ and $L_{ij}^2 \ge (L_\mathrm{max})^2_{ij} = 0$ for $i \ne j$, we obtain maximality of $(L_\mathrm{max})_{ii}$ and hence the maximality of $\det (M_\mathrm{max})$.

### Proof of Theorem 3.2

Under the assumptions of Theorem 3.1, we need to show that the design $\lambda _{n1} = \lambda _\mathrm{max}$ and $\lambda _{n2} = (-1)^{n}\lambda _\mathrm{max}$ for all comparisons $n \in \{n, \ldots , N\}$, which leads to the design matrix $M_\mathrm{max}$, minimizes $\sum _{i=1}^T (M^{-1})_{ii}$. This is equivalent to minimizing the sum of the (univariate) variances of any efficient estimator.

Let us first consider all designs such that *M* is diagonal. In that case, $M^{-1}$ is also diagonal with diagonal entries $(M^{-1})_{ii} = 1/ M_{ii}$. From the proof of Theorem 3.1, we see that $\sum _{i=1}^T M_{ii}$ is bounded and maximized by any design with $\lambda _{n1} = \pm \lambda _\mathrm{max}$ and $\lambda _{n2} = \pm \lambda _\mathrm{max}$, a class of designs to which the $M_\mathrm{max}$ design belongs. Because the function

58 $$\begin{aligned} f(x, y) = \frac{1}{x + y} + \frac{1}{x - y} \end{aligned}$$

with support in $x > y \in \mathbb {R}^{+}$ is minimized at $y = 0$ for any fixed $x \in \mathbb {R}^{+}$, it follows that $\sum _{i=1}^T (M^{-1})_{ii}$ is minimized if both $\sum _{i=1}^T M_{ii}$ is maximized and $M_{ii} = M_{jj}$ for all traits $i,j \in \{1, \ldots , T\}$. These requirements are fulfilled by $M_\mathrm{max}$, proving its A-optimality within the space all designs that imply a diagonal Fisher information.

It remains to be shown that the $M_\mathrm{max}$ design is indeed A-optimal within the space of all admissible designs. For this purpose, we will again use Cholesky decomposition such that $M = L L^T$ with a lower triangular matrix *L*. We first note that $M^{-1} = (L^{-1})^{\mathop {\mathrm {T}}} L^{-1}$, by standard rules of matrix inversion. Since *L* is lower triangular, $L^{-1}$ is also lower triangular and $(L^{-1})_{ii} = 1 / L_{ii}$. Together, this implies

59 $$\begin{aligned} (M^{-1})_{ii}&= ((L^{-1})^{\mathop {\mathrm {T}}} L^{-1})_{ii} \nonumber \\&= (L^{-1})_{ii} + \sum _{i+1}^T (L^{-1})^{\mathop {\mathrm {T}}}_{ij} (L^{-1})_{ji} \nonumber \\&= \frac{1}{L_{ii}^2} + \sum _{i+1}^T (L^{-1})_{ji}^2. \end{aligned}$$

For any non-diagonal *M*, we have $\sum _{i+1}^T (L^{-1})_{ji}^2 \ge 0$, and thus, together the results from the diagonal case, we conclude

60 $$\begin{aligned} \sum _{i=1}^T (M^{-1})_{ii} \ge \sum _{i=1}^T (M_\mathrm{max}^{-1})_{ii}, \end{aligned}$$

which completes the proof.

### Proof of Theorem 3.3

Consider an ordinal model with latent linear normal structure and arbitrary but fixed number of thresholds *K*. Let $X^+$ be the optimal design matrix and $\tau ^+$ be the optimal threshold vector of that ordinal model. Let $\tau ^\star$ be the optimal threshold vector given the choice of $X^\star$ as design matrix that is optimal under the latent linear model. We need to show that

61 $$\begin{aligned} C(\mathbb {I}_\mathrm{TIRT}(X^\star , \tau ^\star )) = C(\mathbb {I}_\mathrm{TIRT}(X^+, \tau ^+)). \end{aligned}$$

Let $\mathbb {O}^K$ be the set of all real ordered vectors of length *K* and let $s_\mathrm{max} := \text {arg}\max _{s \in \mathbb {O}^K} I_{K}(s)$ be the ordered vector maximizing the Information factor $I_{K}$ in the parameterization of Eq. (11). It follows that $\tau ^+_{nk} = s_{\mathrm{max},k} + X^+_n \eta$ and $\tau ^\star _{nk} = s_{\mathrm{max},k} + X^\star _n \eta$ for each $k \in \{1, \ldots , K\}$ are the optimal thresholds belonging to $X^+$ and $X^\star$, respectively. These solutions are always valid as $s_\mathrm{max}$ is ordered and both $X^+_n \eta$ and $X^\star _n \eta$ are independent of *k*, thus $\tau ^+_{n} = (\tau ^+_{n1}, \ldots , \tau ^+_{nK})$ and $\tau ^\star _{n} = (\tau ^\star _{n1}, \ldots , \tau ^\star _{nK})$ are ordered as well. Accordingly, we get ${I}^\star _{nK} = I^+_{nK} = I_{K}(s_\mathrm{max}) =: c_\mathrm{max}$, where ${I}^\star _{nK}$ and $I^+_{nK}$ are the information factors based on $\tau ^\star _{n}$ and $\tau ^+_{n}$, respectively. Notice that $c_\mathrm{max}$ is independent of *n*.

Define $M^+_n := X^{+ \mathop {\mathrm {T}}}_n X^+_n$. If $(X^\star , \tau ^\star )$ was not optimal for the ordinal model, we would have

62 $$\begin{aligned} C(\mathbb {I}_\mathrm{TIRT}(X^\star , \tau ^\star )) = C\left( \sum _{n=1}^N {I}^\star _{nK} M_n^\star \right) > C\left( \sum _{n=1}^N {I}^+_{nK} M_n^+\right) = C(\mathbb {I}_\mathrm{TIRT}(X^+, \tau ^+)), \end{aligned}$$

which implies

63 $$\begin{aligned} C\left( c_\mathrm{max} \sum _{n=1}^N M_n^\star \right) > C\left( c_\mathrm{max} \sum _{n=1}^N M_n^+\right) \end{aligned}$$

because ${I}^\star _{nK} = I^+_{nK} = c_\mathrm{max}$ for all *n*. However, due to optimality of $X^\star$ for the linear model, we have

64 $$\begin{aligned} C\left( \sum _{n=1}^N M_n^\star \right) = C(\mathbb {I}_{LM}(X^\star )) \le C(\mathbb {I}_{LM}(X^+)) = C\left( \sum _{n=1}^N M_n^+\right) . \end{aligned}$$

Thus, by assumption on *C*, it follows that

65 $$\begin{aligned} C\left( c_\mathrm{max} \sum _{n=1}^N \; M^\star _n\right) \le C\left( c_\mathrm{max} \sum _{n=1}^N\; M^+_n\right) , \end{aligned}$$

which contradicts (63). Accordingly, $X^\star$ must also be an optimal design matrix for the ordinal model. As the ordinal model was arbitrary chosen, this conclusions holds for all such ordinal models.

### Proof of Corollary 3.4

Using basic properties of the determinant, we see that the D-optimal criterion $C_D$ defined in (16) satisfies $C_D(c \, M) = C_D(M) / c$ for any $c \in \mathbb {R}^+$. Similarly, using basic properties of matrix inversion, we see that the A-optimal criterion $C_A$ defined in (19) satisfies $C_A(c \, M) = C_A(M) / \sqrt{c}$. Accordingly, the assumptions of Theorem 3.3 are satisfied for both criteria as

66 $$\begin{aligned} C_D(c \, M^\star ) = C_D(M^\star ) / c \le C_D(M) / c = C_D(c \, M) \end{aligned}$$

and

67 $$\begin{aligned} C_A(c \, M^\star ) = C_A(M^\star ) / \sqrt{c} \le C_A(M) / \sqrt{c} = C_A(c \, M) \end{aligned}$$

for any permissible Fisher information matrix *M*. This completes the proof as the design matrix *X* in ordinal TIRT models only depends on the factor loadings $\lambda$.

## Footnotes

1 If $v_i$ was identified such that we could vary $\lambda _i$ and $\phi _i$ independently of each other, then minimal uniqueness $\phi _i \rightarrow 0$ would always be the optimal choice independently of $\lambda _i$ as can be seen, for example, from the proof of Theorem 3.1.
