# Supplementary material for: On the Information Obtainable from Comparative Judgments
Source: Psychometrika. 2022 Feb 8;87(4):1439–72. doi: 10.1007/s11336-022-09843-z (PMC9636126; doi:10.1007/s11336-022-09843-z)
Supplement: Supplementary file 1 — (pdf 15171 KB) [file 11336_2022_9843_MOESM1_ESM.pdf]

On the information obtainable from comparative judgments: Online Supplement

On the information obtainable from comparative judgments: Online Supplement

This online supplement contains additional analytical and numerical results that could not be part of the main text due to space limitations.

1 Supplement A: Information properties of ordinal cumulative models

In this section, I will study some Fisher information properties of ordinal cumulative models (Bürkner & Vuorre, 2019; OCMs, Tutz, 2000). OCMs can be understood as a special case of graded response models (Samejima, 1997) with item parameters assumed to be known and with person parameters modeled as fixed effects. As they share the same underlying structure, OCMs are closely related to binary ordinal and binary comparative judgments models (Brown & Maydeu-Olivares, 2018), and thus also the binary TIRT models (Brown & Maydeu-Olivares, 2011). Thus, understanding OCMs also improves our understanding of the related comparative judgments models. I will establish mathematically that OCMs bridge the gap between binary and continuous (uncategorized) models, in the sense that the information obtainable from OCMs is greater than or equal to binary models (Theorem 1.4) but lower than or equal to continuous models (Theorem 1.5). This may be intuitively clear but is still worthwhile to put into mathematical terms so that the investigation of ordinal TIRT models performed later in the paper rests on solid theoretical grounds.

1.1 The ordinal cumulative models

The OCM assumes that the observed response y is the categorization of a latent continuous variable $\tilde{y}$ with a vector $\tau = (\tau_1, \ldots, \tau_K)$ of ordered inner thresholds that partition the values of $\tilde{y}$ into the $K + 1$ observed categories of y:

$$y = k \Leftrightarrow \tau_{k-1} < \tilde{y} \leq \tau_k \quad \text{for} \quad 1 \leq k \leq K + 1. \tag{1}$$

For notational convenience, the outer thresholds are set to $\tau_0 = -\infty$ and $\tau_{K+1} = \infty$. A regression model is imposed on $\tilde{y}$ via

$$\tilde{y} = h(\eta) + \varepsilon, \tag{2}$$

where $h : \mathbb{R}^T \to \mathbb{R}, \eta \to h(\eta)$ is a once-differentiable function of the (person) parameters η (i.e., the predictor term in a regression model), and ε is a random error following a continuous distribution with cumulative distribution function F. For the purposes of this paper, only the person parameter η are of interest, while the item parameters, including the thresholds τ_k, are considered to be known. The category probabilities $p_k(\eta) := p(Y = k \mid \eta)$ evaluate to

$$p_k(\eta) = F(\tau_k - h(\eta)) - F(\tau_{k-1} - h(\eta)). \tag{3}$$

The log-likelihood $l(y \mid \eta)$ of an OCM for a single observation is equal to the log-likelihood of a categorical model with category probabilities $p_k(\eta)$ chosen as per Equation (3):

$$l(y \mid \eta) = \sum_{k=1}^{K+1} y_k \log(p_k(\eta)), \tag{4}$$

where $y_k = 1$ if $y = k$ and $y_k = 0$ otherwise (one-hot encoding). Assuming conditional independence of observations, the log-likelihood of multiple observations is simply the sum of the log-likelihood values of the individual observations.

Evidently, an OCM with $K = 1$ inner thresholds reduces to binary regression with probabilities $p_1(\eta)$ and $p_2(\eta) = 1 - p_1(\eta)$. For example, logistic regression is a special case of the OCM with one threshold if F is the standard logistic distribution, as is probit regression if F is the standard normal distribution (Bürkner & Vuorre, 2019).

1.2 Fisher Information of ordinal cumulative models

For the purpose of studying the information obtained from OCMs, we need to assume that (a) F is fully specified (i.e., has no unknown parameters); (b) F is twice continuously differentiable such that the corresponding density function f exists and is itself continuously differentiable; (c) the density f has unbounded support (i.e., $f(x) > 0$ for all $x \in \mathbb{R}$). Assumption (a) ensures that the model is identified (Tutz, 2000), (b) ensures that the Fisher information can be computed in all relevant cases (see below), and (c) ensures that the predictor term $h(\eta)$ can take on all real values. In the following, I will assume that F

satisfies the conditions (a) to (c) above. For practical purposes, these assumptions are not limiting because all common distributions applied in OCMs, most notably standard normal, logistic, and extreme value distributions satisfy the assumptions.

The information contained in data y about model parameters η is captured by the Fisher information matrix, which is generally defined as

$$\mathbb{I}(\eta) = \mathbb{E}_y \left[\frac{d\, l(\eta)}{d\, \eta} \frac{d\, l(\eta)}{d\, \eta^{\mathrm{T}}} \right]. \tag{5}$$

In words, the Fisher information is the square of the log-likelihood's gradient with respect to the parameters in expectation over possible data (Lehmann & Casella, 2006). The Fisher information plays a crucial role in both frequentist and Bayesian statistics and constitutes an important tool to study theoretical properties of models. For example, in frequentist statistics, the Fisher information is the inverse of the covariance matrix of an (asymptotically) efficient estimator (Lehmann & Casella, 2006). Thus, understanding the Fisher information of a model provides insights about how accurately parameters can be estimated from a given study design.

Proposition 1.1. *Define* $s_{nk} := \tau_{nk} - h_n(\eta)$ *for every observation* $n \in \{1, \dots, N\}$ *and every threshold* $k \in \{1, \dots, K\}$*, where* τ_{nk} *denotes the* k*th threshold of the* n*th observation and* $h_n(\eta)$ *denotes the predictor term of the* n*th observation. Then, for* N *conditionally independent observations, an OCM with distribution* F *and number of inner thresholds* K *has the Fisher information*

$$\mathbb{I}_{NK}(\eta) = \sum_{n=1}^{N} \left(\sum_{k=1}^{K+1} \frac{(f(s_k) - f(s_{k-1}))^2}{F(s_k) - F(s_{k-1})} \right) \frac{d\, h(\eta)}{d\, \eta} \frac{d\, h(\eta)}{d\, \eta^{\mathrm{T}}}. \tag{6}$$

Proof. Due to additivity of the Fisher information for conditionally independent observations, it is sufficient to show the proposition for a single observation and so we drop the index n below for readability. Set $s_k := \tau_k - h(\eta)$ so that $p_k(\eta) = F(s_k) - F(s_{k-1})$, then we have

$$\frac{d \log(p_k(\eta))}{d\eta} = -\frac{f(s_k) - f(s_{k-1})}{F(s_k) - F(s_{k-1})} \frac{dh(\eta)}{d\eta} \tag{7}$$

from which we obtain

$$\mathbb{I}_{1K}(\eta) = \mathbb{E}_y \left[\left(-\sum_{k=1}^{K+1} y_k \frac{f(s_k) - f(s_{k-1})}{F(s_k) - F(s_{k-1})} \frac{dh(\eta)}{d\eta} \right) \left(-\sum_{k=1}^{K+1} y_k \frac{f(s_k) - f(s_{k-1})}{F(s_k) - F(s_{k-1})} \frac{dh(\eta)}{d\eta^{\mathrm{T}}} \right) \right] \tag{8}$$

$$= \mathbb{E}_y \left[\sum_{k=1}^{K+1} y_k \frac{(f(s_k) - f(s_{k-1}))^2}{(F(s_k) - F(s_{k-1}))^2} \frac{dh(\eta)}{d\eta} \frac{dh(\eta)}{d\eta^{\mathrm{T}}} \right] \tag{9}$$

$$= \mathbb{E}_y \left[\sum_{k=1}^{K+1} y_k \frac{(f(s_k) - f(s_{k-1}))^2}{(F(s_k) - F(s_{k-1}))^2} \right] \frac{dh(\eta)}{d\eta} \frac{dh(\eta)}{d\eta^{\mathrm{T}}}, \tag{10}$$

with the second equality following from the one-hot encoding of $y = (y_1, \ldots, y_{K+1})$. Taking the expectation with respect to y and again using $p_k(\eta) = F(s_k) - F(s_{k-1})$, we conclude

$$\mathbb{I}_{1K}(\eta) = \left(\sum_{k=1}^{K+1} \frac{(f(s_k) - f(s_{k-1}))^2}{F(s_k) - F(s_{k-1})} \right) \frac{dh(\eta)}{d\eta} \frac{dh(\eta)}{d\eta^{\mathrm{T}}}. \tag{11}$$

The conclusion for $N > 1$ observation follows immediately from the additivity of the Fisher information. □

In the following, we will drop the observation index n and only work with a single observation to improve readability. Due to additivity of the Fisher information, all the statement and proofs remain correct with arbitrary number of (conditionally independent) observations. We can now study what happens to the information on η as we increase the number of thresholds K. Intuitively, the information should increase as well, and this is indeed what happens if we restrict ourselves to OCM refinements in the following sense:

Definition 1.2. (Refinement) Let OCM_K be an OCM with K inner thresholds $\tau = (\tau_1, \ldots, \tau_K)$ and let OCM_{K+M} be another OCM with $K + M$ inner thresholds $\tau' = (\tau'_1, \ldots, \tau'_{K+M})$. OCM_{K+M} is called a *refinement* of OCM_K if $\tau \subset \tau'$. Further, if $\tau'_{m-1} < \tau'_m < \tau'_{m+1}$ for any $\tau'_m \in \tau' \setminus \tau$, OCM_{K+M} is called a *strict refinement* of OCM_K.

Refining an OCM simply means adding new thresholds while leaving the existing thresholds untouched. In theory, the new thresholds may be the same as some of the existing thresholds. Such a case may arise in practice if one response category is never chosen at all so that the two corresponding thresholds 'surrounding' that category cannot be distinguished

without prior information. To rule out this pathological case, strictly refining means adding new thresholds so that at least one new threshold is *not* part of the set of existing thresholds. In order to prove Theorem 1.4 below, I make use of a small, somewhat technical lemma.

Lemma 1.3. *Let F be a continuous distribution function with density function f and let $s_0, s_1, s_2 \in \text{support}(F)$ with $s_0 < s_2$ and $s_1 \in [s_0, s_2]$. Then, the following inequality holds:*

$$\frac{(f(s_1) - f(s_0))^2}{F(s_1) - F(s_0)} + \frac{(f(s_2) - f(s_1))^2}{F(s_2) - F(s_1)} \geq \frac{(f(s_2) - f(s_0))^2}{F(s_2) - F(s_0)}. \tag{12}$$

Moreover, the inequality holds strictly if and only if s_1 is in the interior of $[s_0, s_2]$.

Proof. Consider an ordinal model with latent distribution function G being equal to the distribution F truncated at s_0 from below and at s_{K+1} from above, such that

$$G(s) = \frac{F(s) - F(s_0)}{F(s_{K+1}) - F(s_0)} \tag{13}$$

with corresponding density function $g(s) = f(s)(F(s_{K+1}) - F(s_0))^{-1}$ for $s \in [s_0, s_{K+1}]$. Let $s_k := \tau_k - h(\eta)$ for $0 \leq k \leq K+1$ with an ordered threshold vector $(\tau_0, \dots, \tau_{K+1})$ of which τ_0 and τ_{K+1} constitute the outer thresholds. This construction of s_k comes without loss of generality, as for every ordered vector $(s_0, \dots, s_{K+1})$, there is an ordered vector $(\tau_0, \dots, \tau_{K+1})$ such that the imposed equality holds. Using the same approach as in the proof of Proposition 1.1, a lengthy but elementary calculation of the Fisher information leads to

$$\mathbb{I}_{G,K}(\eta) = \mathrm{I}_{G,K}(\eta) \frac{dh(\eta)}{d\eta} \frac{dh(\eta)}{d\eta^{\mathrm{T}}}, \tag{14}$$

with scalar factor

$$\mathrm{I}_{G,K}(\eta) := \sum_{k=1}^{K+1} \left(\frac{f(s_k) - f(s_{k-1})}{F(s_k) - F(s_{k-1})} - \frac{f(s_{K+1}) - f(s_0)}{F(s_{K+1}) - F(s_0)} \right)^2 \left(\frac{F(s_k) - F(s_{k-1})}{F(s_{K+1}) - F(s_0)} \right). \tag{15}$$

Assuming a single parameter $\eta \in \mathbb{R}$ with identity predictor term $h(\eta) = \eta$, the positive semi-definiteness of the Fisher information $\mathbb{I}_{G,K}(\eta)$ implies $\mathrm{I}_{G,K}(\eta) \geq 0$. In the special case of $K = 1$, again a lengthy but elementary calculation reveals

$$\mathrm{I}_{G,1}(\eta) = \frac{1}{F(s_2) - F(s_0)} \left(\frac{(f(s_1) - f(s_0))^2}{F(s_1) - F(s_0)} + \frac{(f(s_2) - f(s_1))^2}{F(s_2) - F(s_1)} - \frac{(f(s_2) - f(s_0))^2}{F(s_2) - F(s_0)} \right). \tag{16}$$

Since $s_2 > s_0$, we have $\frac{1}{F(s_2)-F(s_0)} > 0$ and thus

$$\frac{(f(s_1)-f(s_0))^2}{F(s_1)-F(s_0)} + \frac{(f(s_2)-f(s_1))^2}{F(s_2)-F(s_1)} - \frac{(f(s_2)-f(s_0))^2}{F(s_2)-F(s_0)} \geq 0 \tag{17}$$

due to $\mathrm{I}_{G,K}(\eta) \geq 0$. Because we imposed no restrictions on $(s_0, \ldots, s_{K+1})$ other than it being ordered, this inequality holds for arbitrary real values $s_0 \leq s_1 \leq s_2$ such that $s_0 < s_2$. Moreover, as $\mathbb{I}_{G,1}(\eta)$ is positive definite if and only if s_1 is in the interior of $[s_0, s_2]$, Inequality (17) holds strictly under exactly this condition. □

On the basis of Lemma 1.3, proving that the Fisher information increases through refinement is comparably straightforward.

Theorem 1.4. *Refining an OCM increases its Fisher information. That is, if* OCM_{K+M} *is a refinement of* OCM_K*, we have* $\mathbb{I}_{K+M}(\eta) = c(\eta)\, \mathbb{I}_K(\eta)$ *with a constant* $c(\eta) \geq 1$*. Moreover, if* OCM_{K+M} *is a strict refinement of* OCM_K*, the inequality on* $c(\eta)$ *even holds strictly.*

Proof. of Theorem 1.4. I will prove the theorem for $M = 1$ from which the more general case of $M \geq 1$ directly follows by induction. Without loss of generality, assume that the refined model with $K + M = K + 1$ thresholds has its additional threshold $\tau^\star$ between the first and second threshold of the base model, that is,

$$(\tilde{\tau}_1, \ldots, \tilde{\tau}_{K+1}) = (\tau_1, \tau^\star, \tau_2, \ldots, \tau_K), \tag{18}$$

where $(\tau_1, \ldots, \tau_K)$ is the vector of K ordered thresholds of the base model. We set $s_k := \tau_k - h(\eta)$ and $s^\star := \tau^\star - h(\eta)$. The two Fisher information matrices $\mathbb{I}_{K+1}(\eta)$ and $\mathbb{I}_K(\eta)$ only differ in the their multiplicative scalar factors $\mathrm{I}_{K+1}(\eta)$ and $\mathrm{I}_K(\eta)$ whose difference evaluates to

$$\mathrm{I}_{K+1}(\eta) - \mathrm{I}_K(\eta) = \frac{(f(s^\star)-f(s_1))^2}{F(s^\star)-F(s_1)} + \frac{(f(s_2)-f(s^\star))^2}{F(s_2)-F(s^\star)} - \frac{(f(s_2)-f(s_1))^2}{F(s_2)-F(s_1)} \tag{19}$$

with all other terms canceling out. From Lemma 1.3, we know the above expression is non-negative in case of $\tau_1 \leq \tau^\star \leq \tau_2$ (refinement) or even strictly positive in case of

$\tau_1 < \tau^\star < \tau_2$ (strict-refinement). Accordingly, the constant $c(\eta)$ given by

$$c(\eta) = \left(\frac{(f(s^\star) - f(s_1))^2}{F(s^\star) - F(s_1)} + \frac{(f(s_2) - f(s^\star))^2}{F(s_2) - F(s^\star)} \right) \Big/ \left(\frac{(f(s_2) - f(s_1))^2}{F(s_2) - F(s_1)} \right) \tag{20}$$

satisfies $c(\eta) \geq 1$ (refinement) or even $c(\eta) > 1$ (strict refinement). □

In particular, Theorem 1.4 implies that binary models, that is, ordinal models with a single inner threshold are the worst case scenario information-wise provided that all other aspects of the test design remain the same. Conversely, when increasing the number of thresholds, one can show (Theorem 3.1 in Schmidt & Schwabe, 2015) that in the limit of infinite response categories, the OCM looses no information compared to modeling the latent variable $\tilde{y}$ directly via the corresponding continuous model. Formulating this result in here-used notation, it reads as follows:

Theorem 1.5. *Let* $\mathbb{I}_{\tilde{y}}(\eta)$ *denote the Fisher information of the continuous model on* $\tilde{y} = h(\eta) + \varepsilon$. *Then,* $\lim_{K \to \infty} \mathbb{I}_K(\eta) = \mathbb{I}_{\tilde{y}}(\eta)$ *for any series* $(\mathrm{OCM}_K)_{K \geq 1}$ *of refinements such that* $\lim_{K \to \infty} p_k(\eta) = 0$ *for all* $k \in \{1, \ldots, K\}$.

Proof. Under the OCM assumptions stated in Section 1.2, the proof proceeds as the proof of Theorem 3.1 in Schmidt and Schwabe (2015). □

Together with Theorem 1.4, this establishes that the OCM fully bridges the information gap between binary and continuous models. Of course, the truly continuous model is unachievable in practice, but we can at least aim to approximate it well enough using ordinal models with sufficient number of categories. Here, I have studied the information obtainable from OCMs for a general class of distribution functions F and arbitrary predictor terms $h(\eta)$. In the following sections, I will focus more narrowly on the important special case of comparative judgments expressed via Thurstonian IRT models.

2 Supplement B: Bayesian Optimal Test Designs

In Section 3.1 in the main text, I have investigated optimal test designs from a purely frequentist perspective by minimizing optimality criteria that only consider the Fisher information matrix M. Although the obtained results are highly useful already, a drawback of these criteria is that they do not take into account other central aspects of TIRT trait score estimation, most importantly the sampling correlation matrix Σ_η and prior correlation matrix Σ_{prior}. Incorporating these aspects into optimal design criteria is part of the field of *Bayesian optimal design*. There are several perspectives from which one can approach Bayesian optimality (for an overview see Chaloner & Verdinelli, 1995). Below, I will consider and discuss three Bayesian perspectives, two of which are of general nature while the third is specifically tailored to estimation accuracy in the here-considered Bayesian linear models for comparative judgments.

The first perspective on Bayesian optimal design is to take the sampling distribution $p(\eta)$ (and thus Σ_η) into account while still performing frequentist inference. This approach is most common in the classical optimal design literature and often referred to as *pseudo-Bayesian* optimal design, because no prior is used for inference (Bürkner, Schwabe, & Holling, 2019). Formally, pseudo-Bayesian optimal designs are derived by optimizing the integral of a frequentist criterion over the parameters' sampling distribution. For example, a pseudo-Bayesian D-optimality criterion can be defined as

$$C_D^{\text{pB}}(\lambda) := \int C_D(\lambda, \eta)\, p(\eta)\, d\eta, \tag{21}$$

where $C_D(\lambda, \eta)$ is the D-optimality criterion and $p(\eta)$ is the assumed person parameters' sampling distribution. A Pseudo-Bayesian A-optimality criterion $C_A^{\text{pB}}(\lambda)$ can be defined analogously. For linear TIRT models, pseudo-Bayesian optimal designs are straightforward to derive as $C_D(\lambda, \eta) = C_D(\lambda)$ does not depend on the parameters η. Accordingly an optimal design is globally optimal for all η and thus $C_D^{\text{pB}}(\lambda) = C_D(\lambda)$ independently of $p(\eta)$. When considering ordinal TIRT models, $M(\lambda, \tau, \eta)$ and hence the corresponding pseudo-Bayesian

optimality criteria depend on η only through the information factor (Equqation 8 in the main text). And we know from Theorem 3.1 that the (frequentist) optimal factor loadings λ are globally optimal also in the ordinal case. Hence, they will remain the same when considering pseudo-Bayesian optimality. Only the optimal thresholds τ a likely to change under a pseudo-Bayesian perspective (see Bürkner, Schwabe, & Holling, 2019 for related work) but a closer investigation is out of scope of this paper.

The second perspective is to optimize the Bayesian Fisher information, which is defined as the Fisher information plus the prior information (e.g., Gill & Levit, 1995). From Equation (23) in the main text, we see that the inverse of the posterior covariance matrix

$$M_{\text{post}} := \Sigma_{\text{post}}^{-1} = M + \Sigma_{\text{prior}}^{-1}, \tag{22}$$

of a Bayesian linear model is nothing else than the Bayesian Fisher information. I define

$$C_D^B(\lambda) := C_D^B(M_{\text{post}}(\lambda)) := \det(M_{\text{post}}^{-1})^{1/T} = \det(M_{\text{post}})^{-1/T}, \tag{23}$$

as the Bayesian D-optimality criterion and

$$C_A^B(\lambda) := C_A^B(M_{\text{post}}(\lambda)) := \sqrt{\frac{1}{T}\sum_{i=1}^{T}(M_{\text{post}}^{-1})_{ii}} \tag{24}$$

as the Bayesian A-optimality criterion, analogously to their frequentist counterparts. Both of these Bayesian criteria can be justified by Bayesian decision theoretical considerations (Chaloner & Verdinelli, 1995). Again, it is sensible to ask how item parameters should be chosen in order to optimize these criteria. If the prior Σ_{prior} is diagonal, its inverse $\Sigma_{\text{prior}}^{-1}$ is also diagonal. It follows that the optimal design is the same as if we did not add any prior information due to the properties of the frequentist optimal designs discussed in Section 3.1 in the main text. If Σ_{prior} (and correspondingly $\Sigma_{\text{prior}}^{-1}$) is not diagonal, the Bayesian D-optimal and A-optimal designs are not necessarily available in closed form and one needs to resort to numerical optimization methods for a given prior.

Below, I will illustrate Bayesian D- and A-optimal designs for $T = 5$ traits under

selected design and prior conditions, by varying the following factors in a fully crossed manner:

- The design type: Either a mixed keyed design (half equally and half unequally keyed pairs) denoted as (+/-) or a fully equally keyed design denoted as (+).
- The number of item pairs per trait combination $R = 2, 6, 10$. For $T = 5$ traits, this implies $N = 20, 60, 100$ number of item pairs in total.
- The prior correlation matrix Σ_{prior}: One of two choices taken from the NEO-PI-R (Costa & McCrae, 1992; Ostendorf & Angleitner, 2004). Either the correlation matrix of neuroticism, extraversion, conscientiousness, agreeableness, and openness to experiences as also used in other TIRT-related papers (Brown & Maydeu-Olivares, 2011; Bürkner, Schulte, & Holling, 2019), or the same correlation matrix but with neuroticism inverted so that higher values indicate more emotional stability. The former correlation matrix contains a mix of negative, positive, and zero correlations and we will be denoted as NEO(+/-). The latter correlation matrix contains only non-negative correlation and will be denoted as NEO(+).

For the resulting design and prior conditions, the Bayesian D- and A-optimality criteria are displayed in Figure 1 and 2, respectively, as a function of the mean factor loading $\bar{\lambda}$ and the factor loading difference λ_Δ (compare Section 3.1 in the main text). For mixed keyed designs, using maximal factor loadings $\bar{\lambda}$ and $\lambda_\Delta = 0$ is both Bayesian D- and A-optimal (see the right-hand sides of Figures 1 and 2). This is unsurprising given how informative these mixed keyed designs are as compared to alternatives. We would need to have an extreme prior and very few item pairs to induce another design to be optimal. However, maximal factor loadings $\bar{\lambda}$ and $\lambda_\Delta = 0$ are also Bayesian D-optimal for equally keyed designs (see the left-hand side of Figure 1). This result is counter-intuitive as such choices of factor loadings imply zero information on the within-person parameter mean and hence non-identified person parameters (Brown, 2016). Unfortunately, I do not have a

satisfactory explanation for this result at this point in time but in any case, the sensibility of such Bayesian D-optimal designs for comparative judgments should be called into question. In contrast, Bayesian A-optimal designs in the equally keyed case are more sensible as they imply a balance between high factor loadings and high factor loading differences, as one would expect. This balance depends on the number of item pairs and on the prior correlation matrix (see the left-hand side of Figure 2). Specifically, more positively correlated traits imply higher factor loading differences to be optimal, up to a point where $\lambda_\Delta = \bar{\lambda}/2$ such that one of two factor loadings per item pair is zero (see top row of Figure 2).

Using optimality criteria based on the Bayesian Fisher information has two main drawbacks. First, such criteria have no notion of a true sampling distribution but only of a prior. Hence, potential misspecification of the prior cannot be accounted for by these criteria. Second, in the reliability and expected RMSE measures, the Fisher information M does not only appear in the context of the Bayesian Fisher information M_{post} but also independently thereof (see Section 3.3 in the main text). Accordingly, optimizing M_{post} may leave M itself to be highly non-optimal thus reducing the actual person parameter accuracy.

This naturally leads to the third perspective on Bayesian optimal designs for comparative judgments, which is to consider designs that maximize reliability or minimize expected RMSE. Such designs are not only most directly related to what we are aiming to achieve in the end, but also incorporate both prior and sampling correlation matrix and hence potential prior misspecification. Below, I illustrate reliability and expected RMSE for the same conditions as for Bayesian D- and A-optimal designs but add another factor to account for prior misspecification: The prior correlation matrix Σ_{prior} may either be equal to Σ_η or diagonal, that is, with all correlations set to zero. The latter is the maximum entropy choice in the absence of any prior knowledge about the correlations between traits.

As displayed in Figure 3 optimal designs for the reliability closely resemble the Bayesian A-optimal designs: For mixed keyed designs maximal factor loadings are optimal

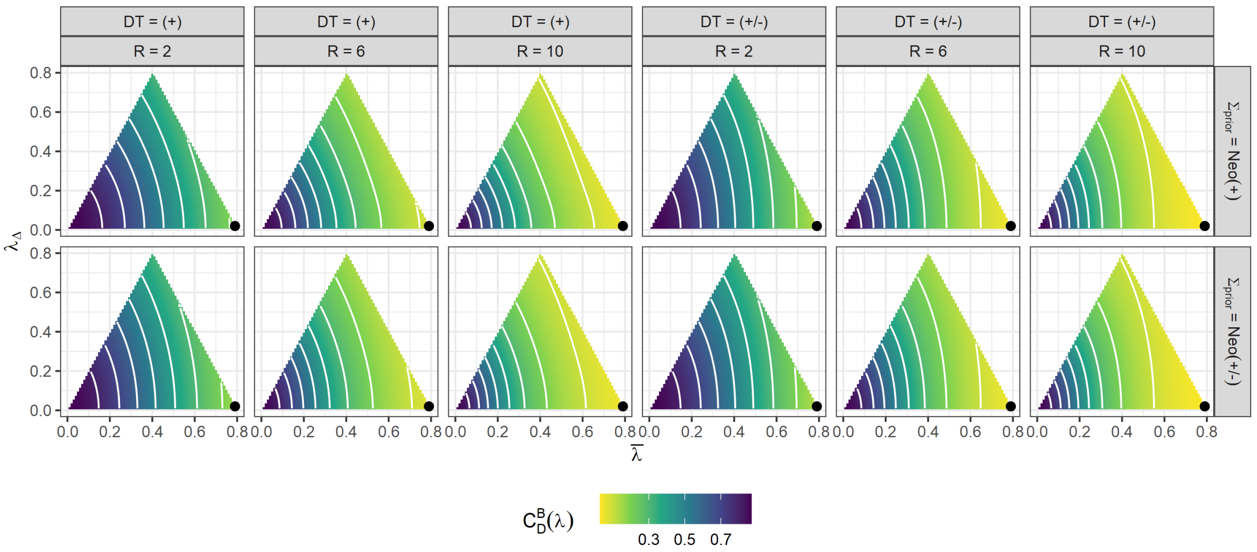

Figure 1. Bayesian D-optimality criterion for $T = 5$ traits as a function of the mean factor loading $\bar{\lambda}$ and factor loading difference λ_Δ. Brighter colors indicate better values. Black dots indicate the location of the optimal design. Abbreviations: DT = design type; R = number of comparisons per trait combination; Σ_{prior} = prior correlation matrix.

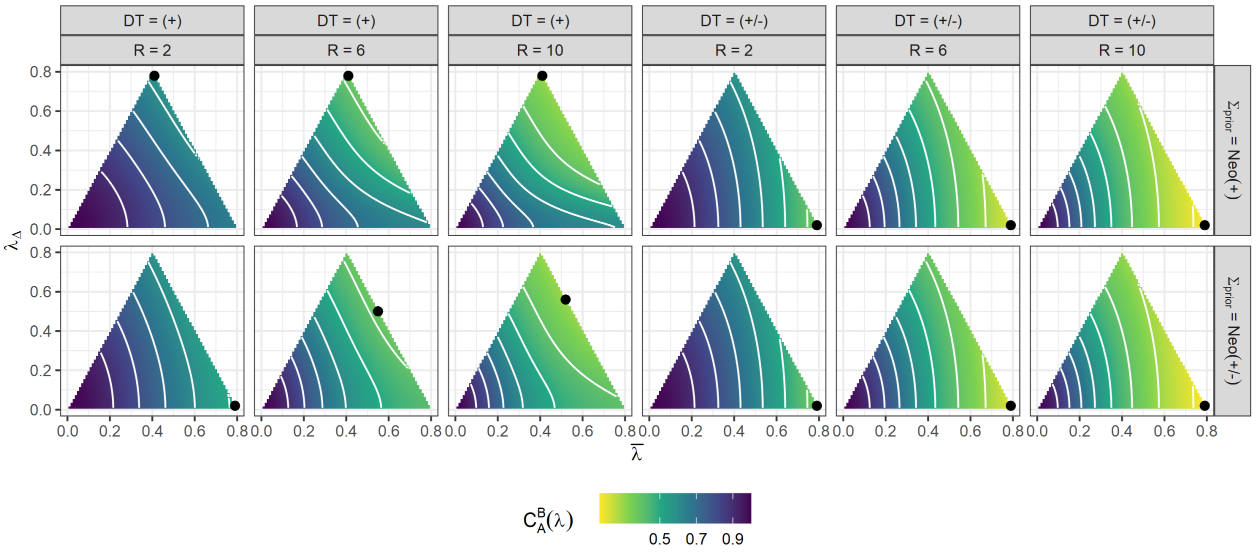

Figure 2. Bayesian A-optimality criterion for $T = 5$ traits as a function of the mean factor loading $\bar{\lambda}$ and factor loading difference λ_Δ. Brighter colors indicate better values. Black dots indicate the location of the optimal design. Abbreviations: DT = design type; R = number of pairs per trait combination; Σ_{prior} = prior correlation matrix.

throughout while for equally keyed designs, there is a trade-off between high mean factor loadings and high factor loading differences. In particular, for more positively correlated traits, a maximal factor loading difference is optimal which implies one of the two factor loadings per item pair to be zero. At least for $T = 5$ traits, using a misspecified diagonal prior does not change the optimal design noticeably. The optimal designs for the expected RMSE are highly similar to those of the reliability within a given condition (see Figure 4).

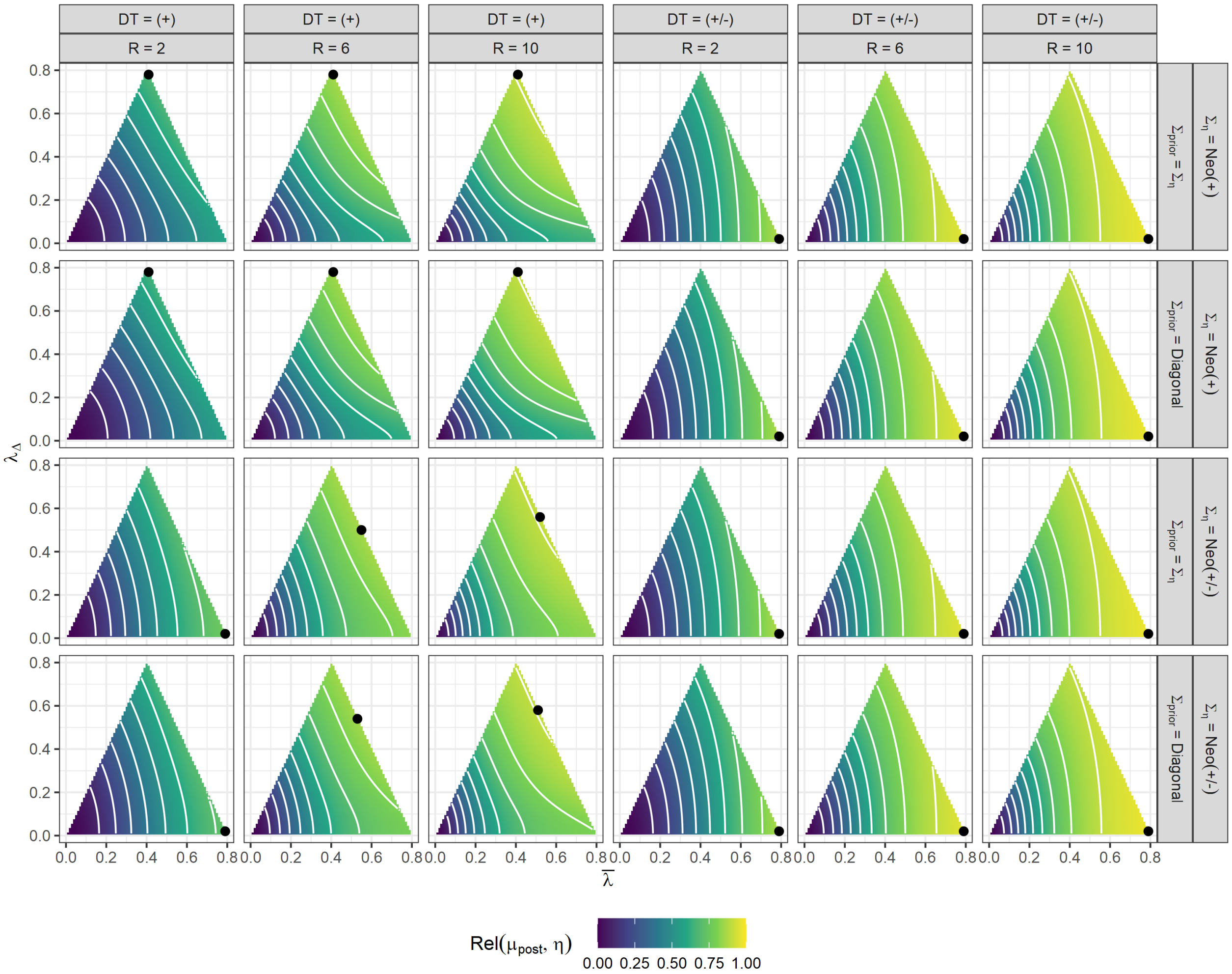

Figure 3. Reliability criterion for $T = 5$ traits as a function of the mean factor loading $\bar{\lambda}$ and factor loading difference λ_Δ. Brighter colors indicate better values. Black dots indicate the location of the optimal design. Abbreviations: DT = design type; R = number of pairs per trait combination; Σ_η = true sampling correlation matrix; Σ_{prior} = prior correlation matrix.

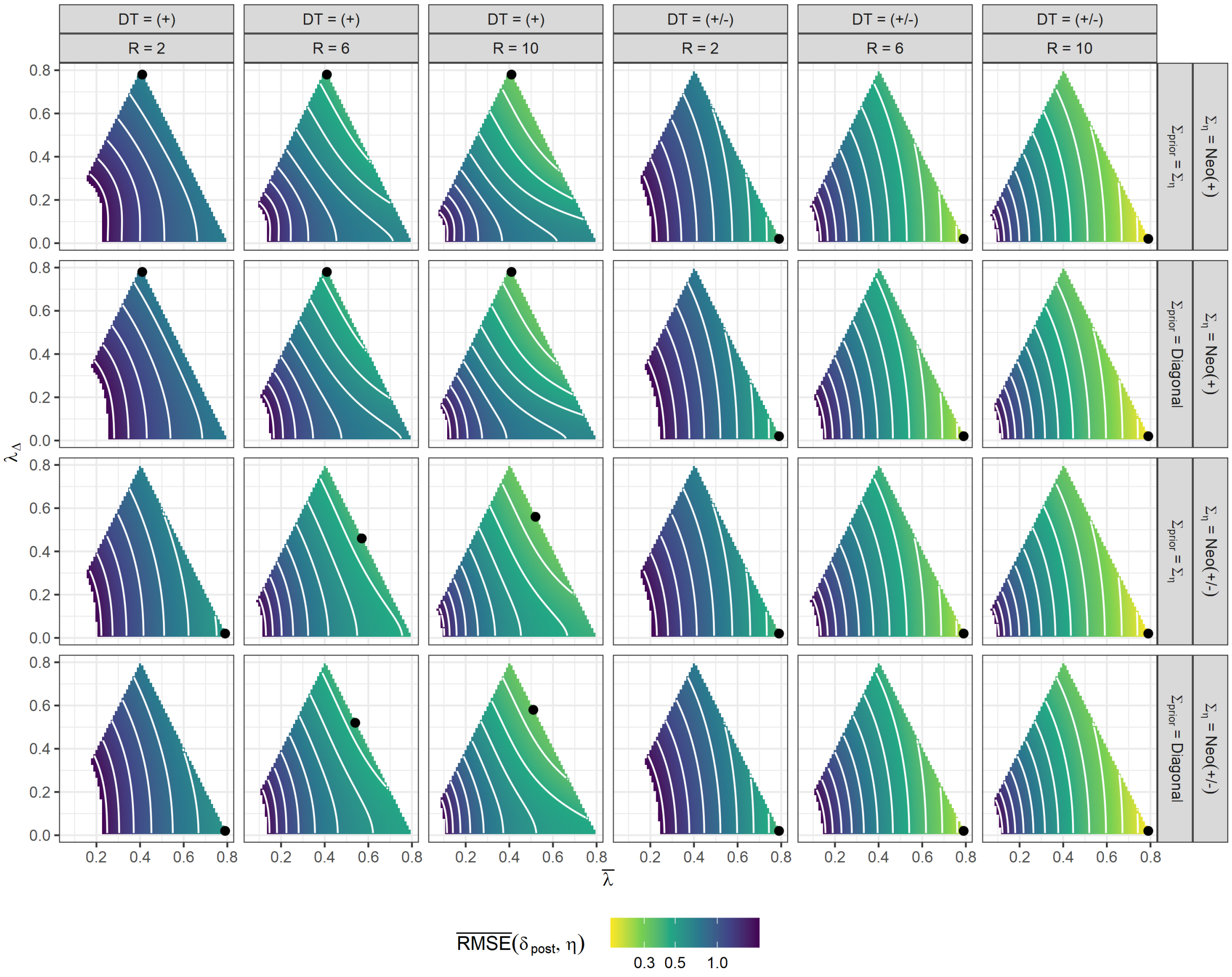

Figure 4. RMSE criterion for $T = 5$ traits as a function of the mean factor loading $\bar{\lambda}$ and factor loading difference λ_Δ. Brighter colors indicate better values. Black dots indicate the location of the optimal design. The left-most part of the grids are not shown to avoid obfuscating the color scale. Abbreviations: DT = design type; R = number of pairs per trait combination; Σ_η = true sampling correlation matrix; Σ_{prior} = prior correlation matrix.

3 Supplement C: Varying the total number of item pairs

Here, I present additional results obtained in the numerical experiments described in Section 4.2 in the main text, where I systematically varied the total number of item pairs B.

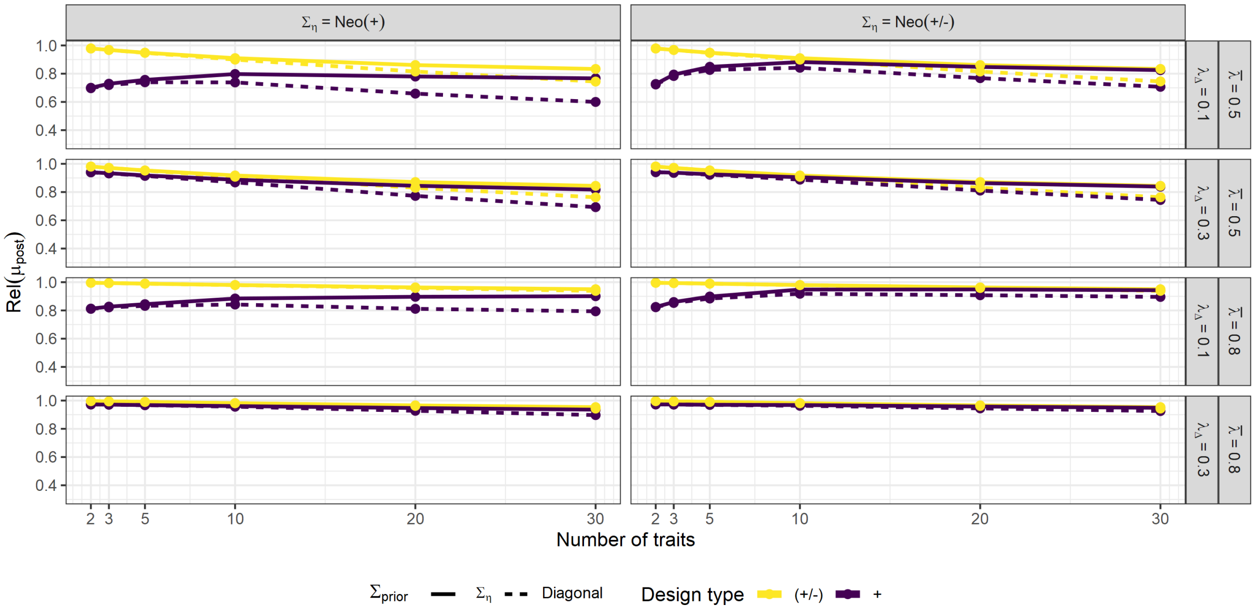

Figure 5. Person-trait-specific RMSEs (dots) for $B = 270$ number of comparisons as a function of the true trait scores η. Expected RMSEs are shown as horizonal lines. Abbreviations: T = number of traits; $\bar{\lambda}$ = mean factor loading; λ_Δ = factor loading difference; Σ_η = true sampling correlation matrix.

The shrinkage of parameter estimates induced by the prior is not the cause for any of the RMSE patterns identified in the main text, as all figures display the RMSE of δ_{post}, an estimate of η from which prior shrinkage was removed already. In comparison, the corresponding results for μ_{post}, which still contains prior shrinkage, show an even stronger dependency on $\bar{\eta}$ (see Figure 8). In contrast to the within-person mean $\bar{\eta}$, the within-person root mean squared difference (RMSD) of the η values cannot explain much of the RMSE variation in any of the conditions (see Figure 9). This clearly demonstrates that differences in between traits of the same person can be estimated well as long as equally keyed item pairs are present confirming theoretical results (see also Brown & Maydeu-Olivares, 2011).

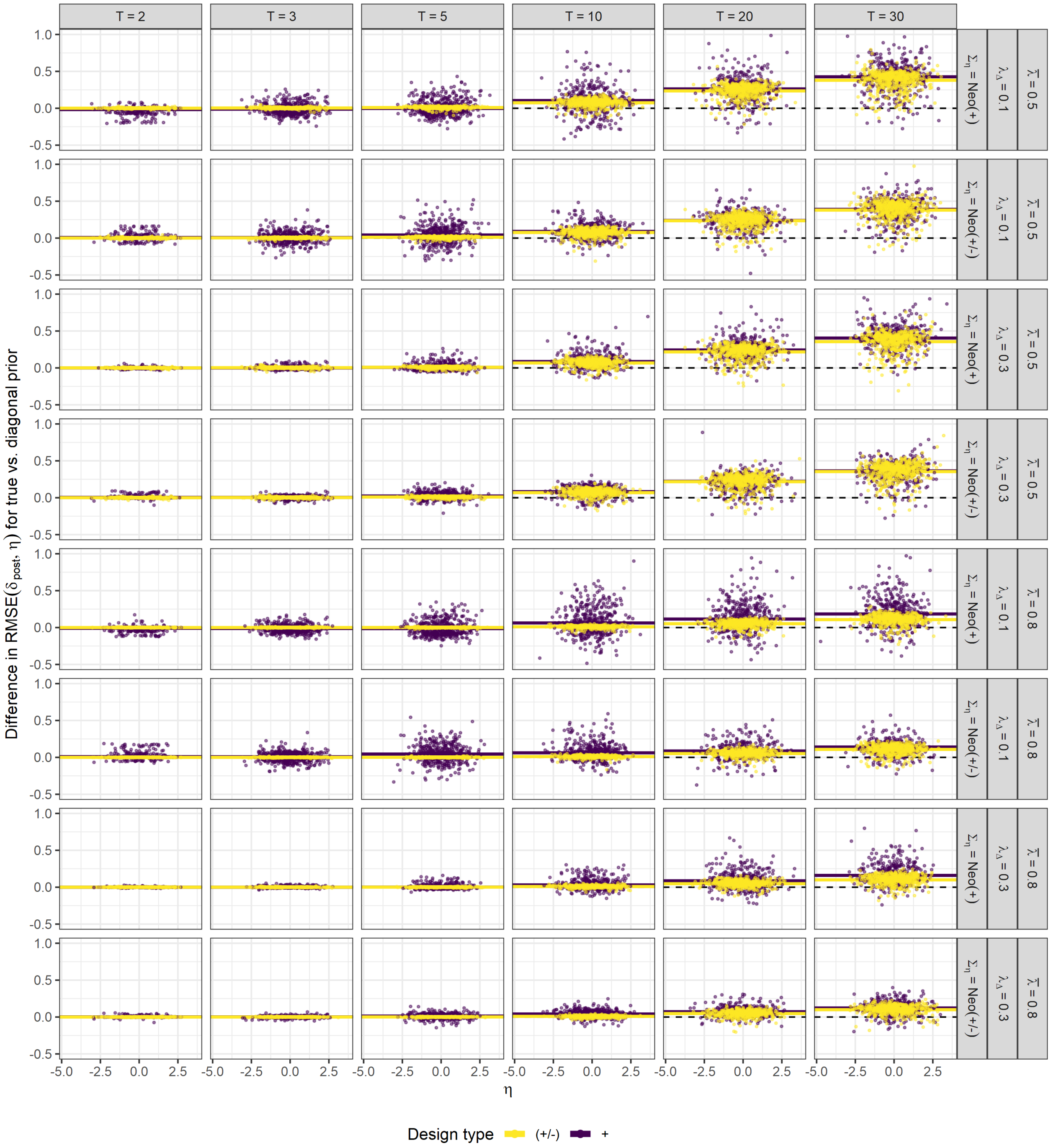

Figure 6. Person-trait-specific RMSE differences (dots) between using the true prior ($\Sigma_{\text{prior}} = \Sigma_\eta$) and a diagonal prior. Results are displayed for $B = 90$ number of comparisons as a function of the true trait scores η. Expected RMSEs are shown as horizonal lines. Abbreviations: T = number of traits; $\bar{\lambda}$ = mean factor loading; λ_Δ = factor loading difference; Σ_η = true sampling correlation matrix.

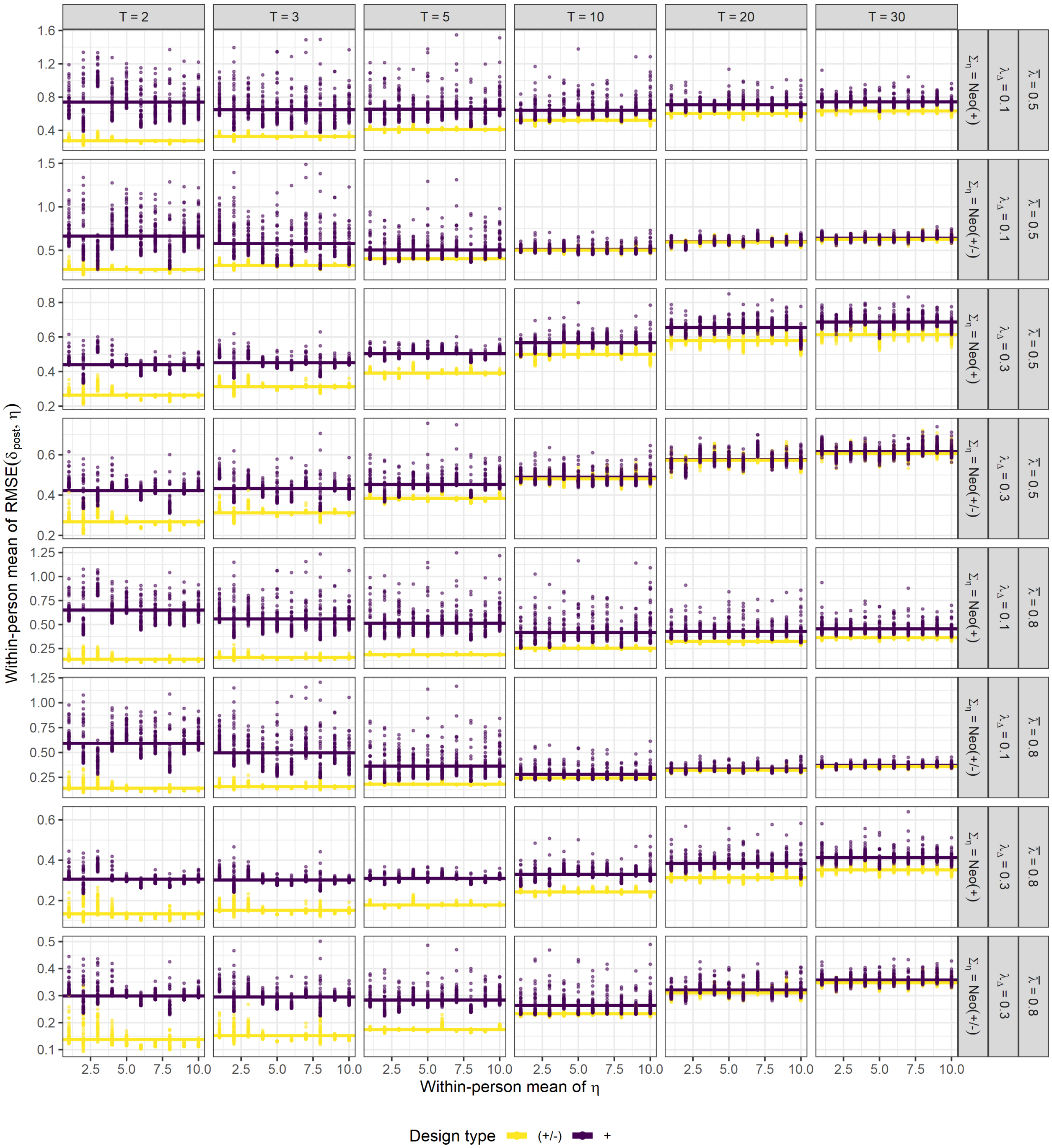

Figure 7. Person-trait-specific RMSEs (dots) for $B = 90$ number of comparisons as a function of the simulation trial. Expected RMSEs are shown as horizonal lines. Abbreviations: T = number of traits; $\bar{\lambda}$ = mean factor loading; λ_Δ = factor loading difference; Σ_η = true sampling correlation matrix.

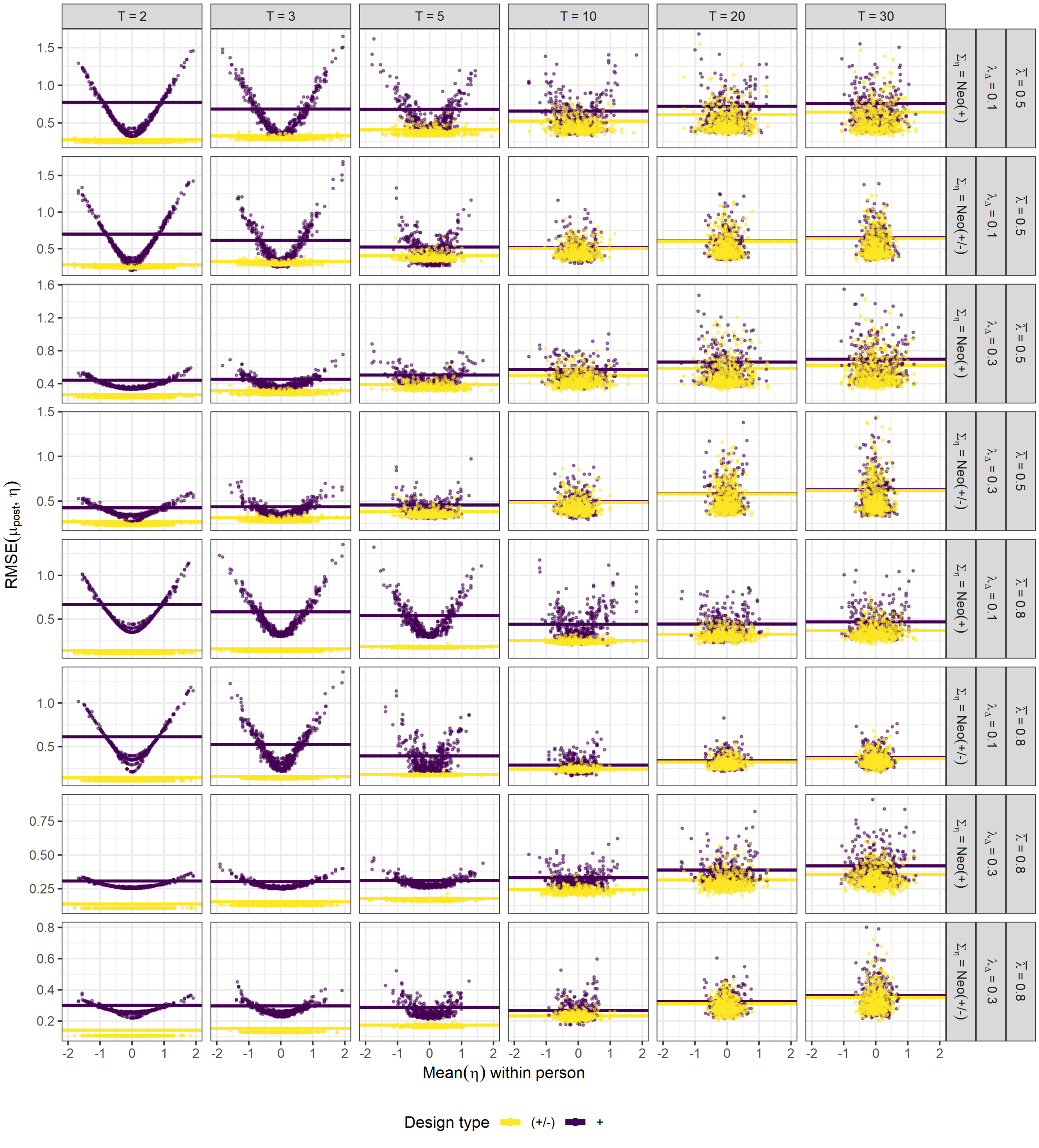

Figure 8. Person-trait-specific RMSEs (dots) for the original posterior mean estimate μ_{post} which is affected by prior shrinkage. Results are shown for $B = 90$ number of comparisons as a function of the true trait scores η. Expected RMSEs are shown as horizonal lines. Abbreviations: T = number of traits; $\bar{\lambda}$ = mean factor loading; λ_Δ = factor loading difference; Σ_η = true sampling correlation matrix.

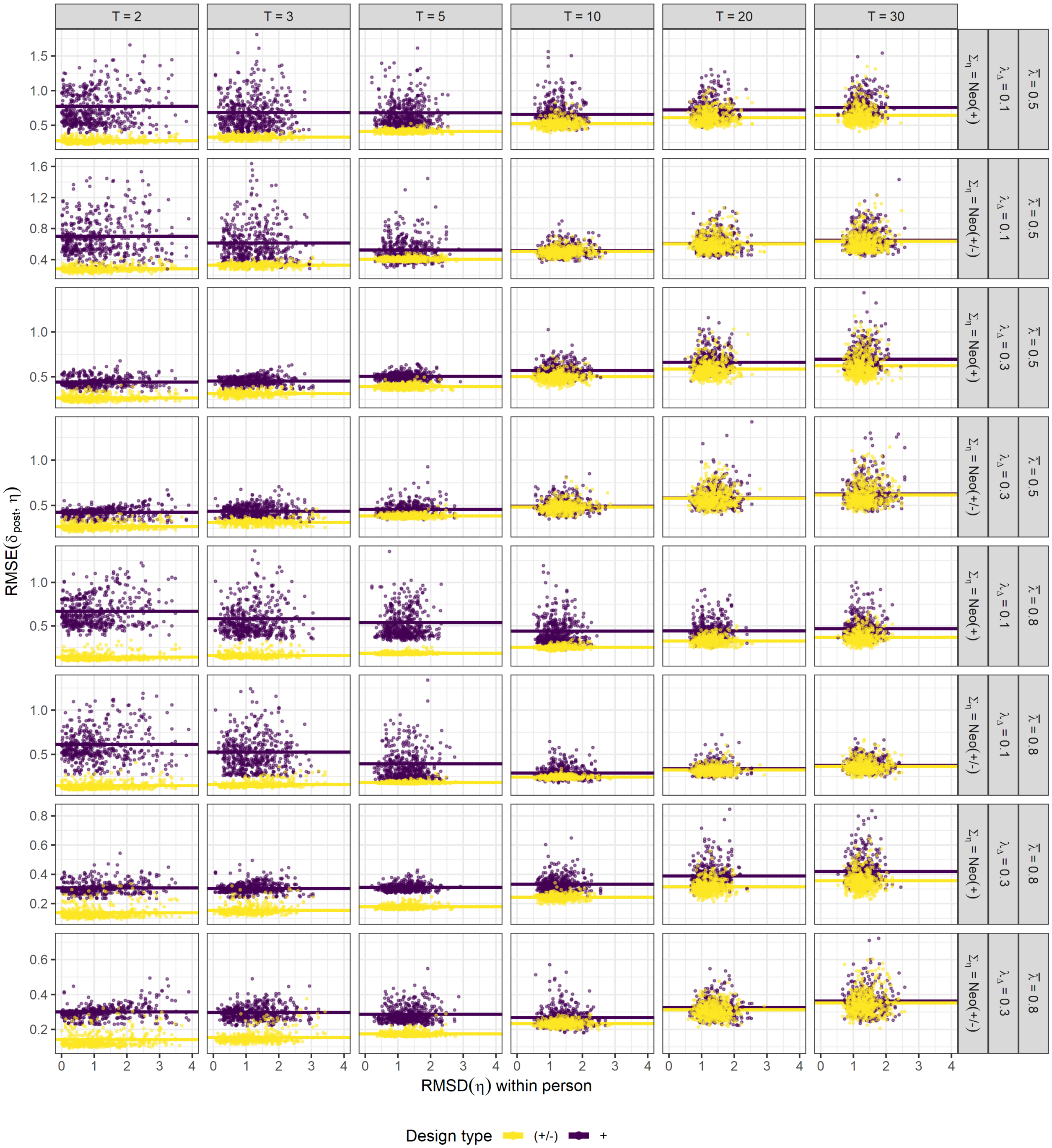

Figure 9. Person-specific RMSEs (dots; averaged over traits) for $B = 90$ number of comparisons as a function of the true within-person root mean-squared differences RMSDη. Expected RMSEs are shown as horizontal lines. Abbreviations: T = number of traits; $\bar{\lambda}$ = mean factor loading; λ_Δ = factor loading difference; Σ_η = true sampling correlation matrix.

As a third, somewhat indirect measure of estimation accuracy, let us consider the inter-trait correlation matrix, which is a scaled version of the covariance matrix $\mathrm{Var}_{\tilde{y},\eta}(\mu_{\mathrm{post}})$:

$$\mathrm{Cor}_{\tilde{y},\eta}(\mu_{\mathrm{post}}) = \tilde{S}\mathrm{Var}_{\tilde{y},\eta}(\mu_{\mathrm{post}})\tilde{S}, \tag{25}$$

where $\tilde{S}$ is a diagonal scaling matrix with diagonal elements $\tilde{S}_{ii} = (\mathrm{Var}_{\tilde{y},\eta}(\mu_{\mathrm{post}})_{ii})^{-1/2}$. This correlation matrix is interesting to study as the factors influencing $\mathrm{Cor}_{\tilde{y},\eta}(\mu_{\mathrm{post}})$ will also naturally influence estimates of Σ_{prior}, if the latter is estimated. Remember that, in this paper, I consider Σ_{prior} known for the purpose of the mathematical analysis; but in practice, the prior correlation matrix will represent a hyperparameter to be estimated from the data (Brown & Maydeu-Olivares, 2011). From Equation 41 in the main text, we see that $\mathrm{Cor}_{\tilde{y},\eta}(\mu_{\mathrm{post}}) \Rightarrow \Sigma_\eta$ as the test information approaches infinity. However, as is clear from Equation 43 in the main text, the finite sample behavior of $\mathrm{Cor}_{\tilde{y},\eta}(\mu_{\mathrm{post}})$ may be very different depending on the test design.

For the same selected conditions shown for reliability and RMSE in the main text, Figure 10 illustrates the expected absolute bias of the trait estimates' correlation matrix $\mathrm{Cor}_{\tilde{y},\eta}(\mu_{\mathrm{post}})$ with respect to true trait score correlation matrix Σ_η. This correlation bias is particularly strong for equally keyed designs with smaller number of traits ($T \leq 5$) and small factor loading differences ($\lambda_\Delta = 0.1$). Under these conditions, trait score estimates show the strongest partial ipsativity as within-person trait scores means cannot be estimated well from such designs. In most other conditions, biases are relatively small (bias < 0.1) except in cases with a misspecified (diagonal) prior Σ_{prior} where biases may be much larger, in particular for equally keyed designs.

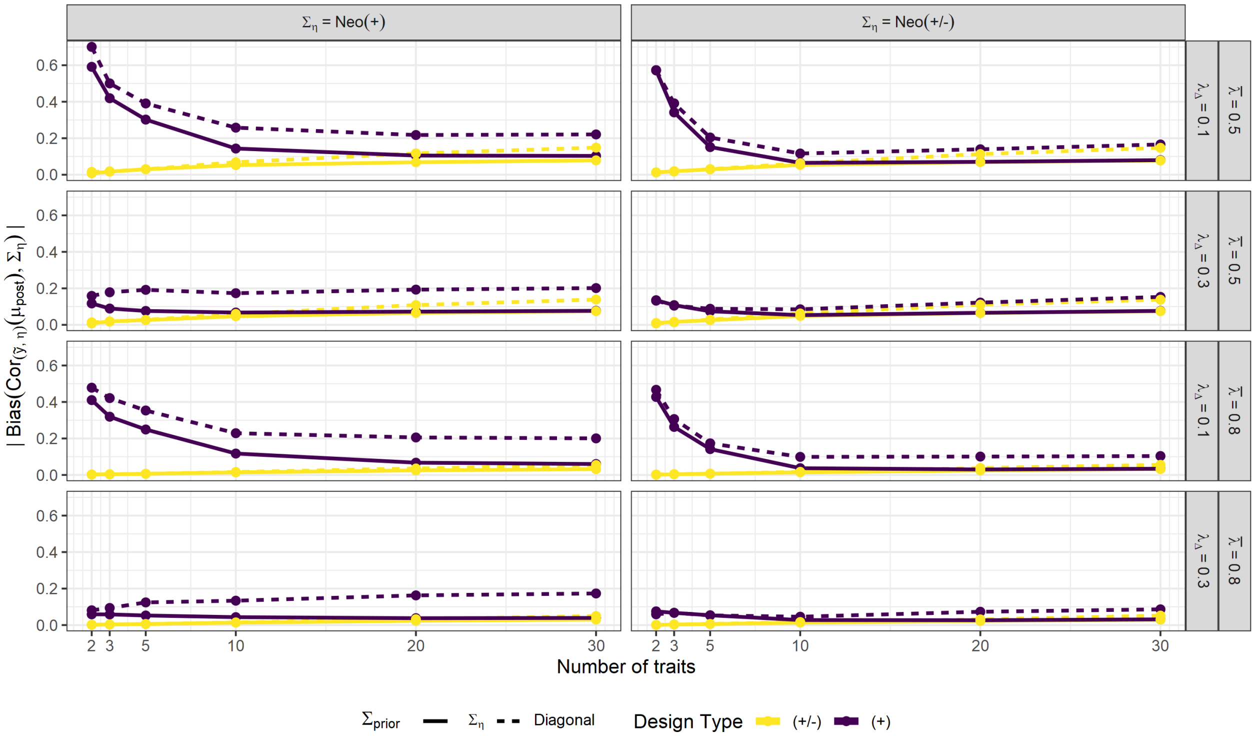

Figure 10. Expected absolute bias of the estimated trait scores' inter-correlations for $B = 90$ number of item pairs as a function of the number of traits T. Abbreviations: $\bar{\lambda}$ = mean factor loading; λ_Δ = factor loading difference; Σ_η = true sampling correlation matrix; Σ_{prior} = prior correlation matrix.

4 Supplement D: Varying the number of item pairs per trait

Here, I present results obtained from additional numerical experiments comparable to those described Section 4.2 in the main text. However, instead of systematically varying the total number of item pairs B, I systematically varied the number of item pairs per trait B_T, which took on values of $B_T = 12, 24$. Figures 11, 12, and 13 display the same conditions as Figures 8, 9, and 10 in the main text, except that $B_T = 12$ instead of $B = 90$ was used.

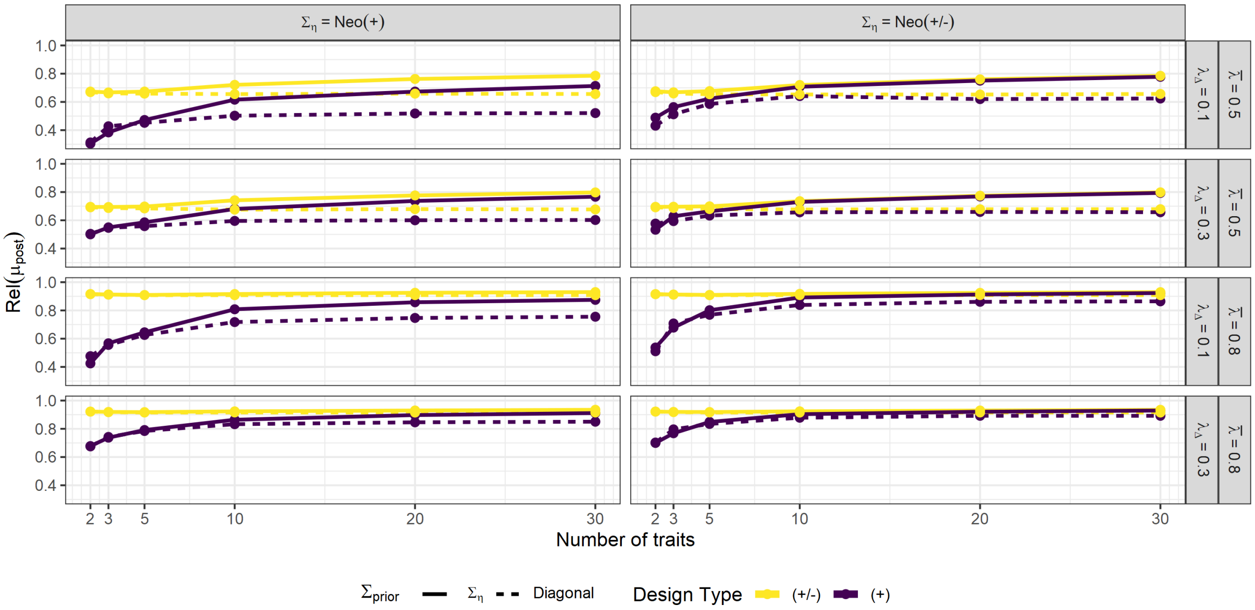

Figure 11. Expected reliability for $B_T = 12$ number of comparisons per trait combination as a function of the number of traits T. Abbreviations: $\bar{\lambda}$ = mean factor loading; λ_Δ = factor loading difference; Σ_η = true sampling correlation matrix; Σ_{prior} = prior correlation matrix.

5 Supplement E: Increasing test information by measuring more traits

In addition to the two mechanisms identified in the main text of the paper, there is a third mechanism related to the true sampling correlation matrix Σ_η. This is was first identified by Baron (1996) and is discussed in some more detail below for reasons of completeness. As the number of traits increases, the variance of the within-person mean $\bar{\eta}$ across individuals decreases. In Figure 10 in the main text, we had seen that the strikingly high RMSEs for individuals with overall low or high $\bar{\eta}$ appear much less frequently when increasing the number of traits to $T \geq 10$. This can be explained as follows: When increasing the number of traits measured within a test, more extreme within-person means $\bar{\eta}$ become less likely as the variance of $\bar{\eta}$ reduces with increasing T. In the most simple case, for T uncorrelated traits each with $\text{Var}_\eta(\eta_i) = 1$, we have $\text{Var}_\eta(\bar{\eta}) = \frac{1}{T}$. This variance further decreases through negative correlations between traits, while it increases through positive correlations, essentially explaining the differences between the conditions $\Sigma_\eta = \text{Neo}(+/-)$

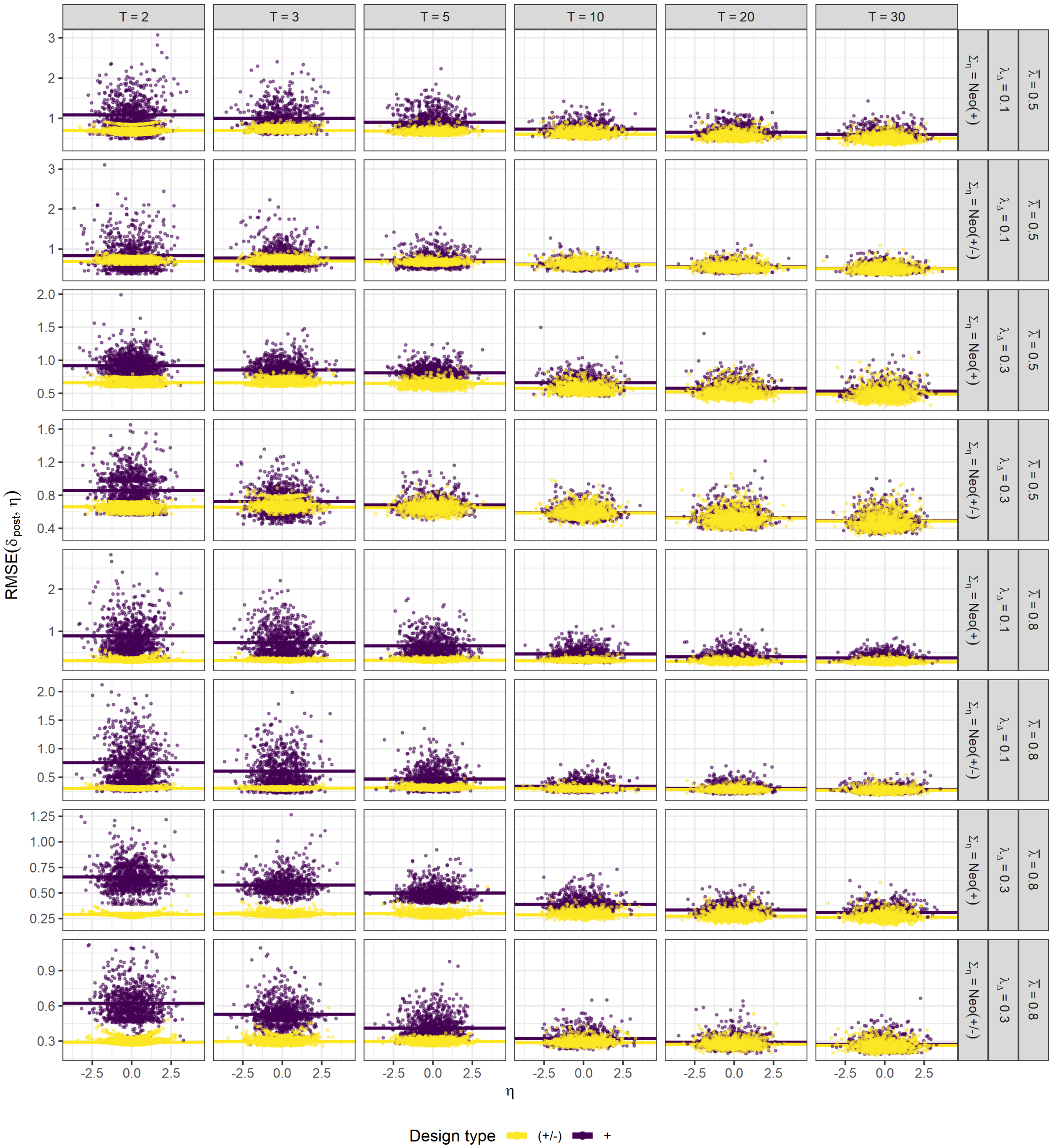

Figure 12. Person-trait-specific RMSEs (dots) for $B_T = 12$ number of comparisons per trait combination as a function of the true trait scores η. Expected RMSEs are shown as horizonal lines. Abbreviations: T = number of traits; $\bar{\lambda}$ = mean factor loading; λ_Δ = factor loading difference; Σ_η = true sampling correlation matrix.

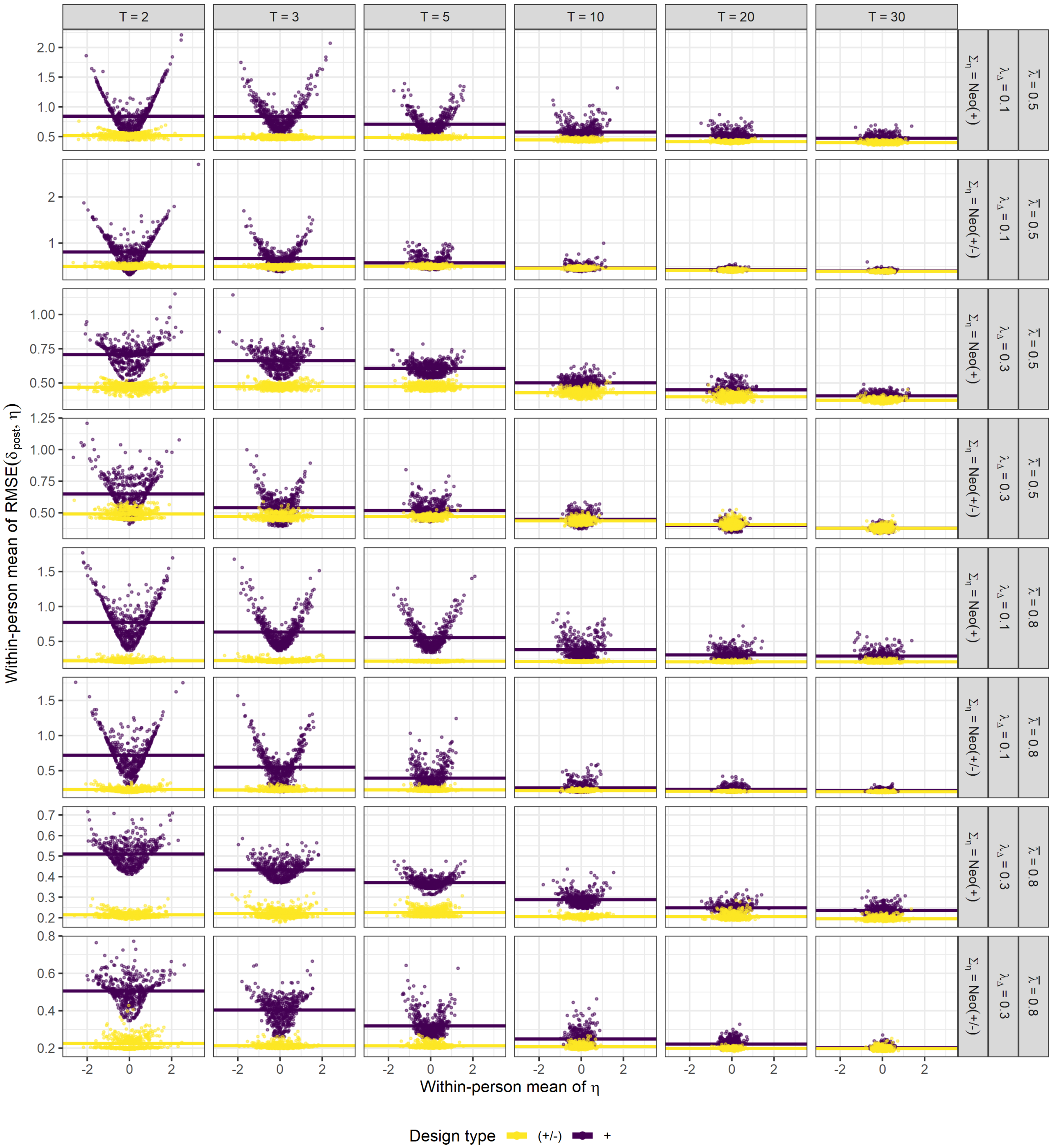

Figure 13. Person-specific RMSEs (dots; averaged over traits) for $B_T = 12$ number of comparisons per trait combination as a function of the true within-person trait score mean $\bar{\eta}$. Expected RMSEs are shown as horizonal lines. Abbreviations: T = number of traits; $\bar{\lambda}$ = mean factor loading; λ_Δ = factor loading difference; Σ_η = true sampling correlation matrix.

and $\Sigma_\eta = \text{Neo}(+)$, as shown in the top panel of Figure 14. However, *if* there was a person with an extreme average trait score, their estimates would still have show comparably high RMSE even for a large number of traits, as shown in the bottom panel of Figure 14.

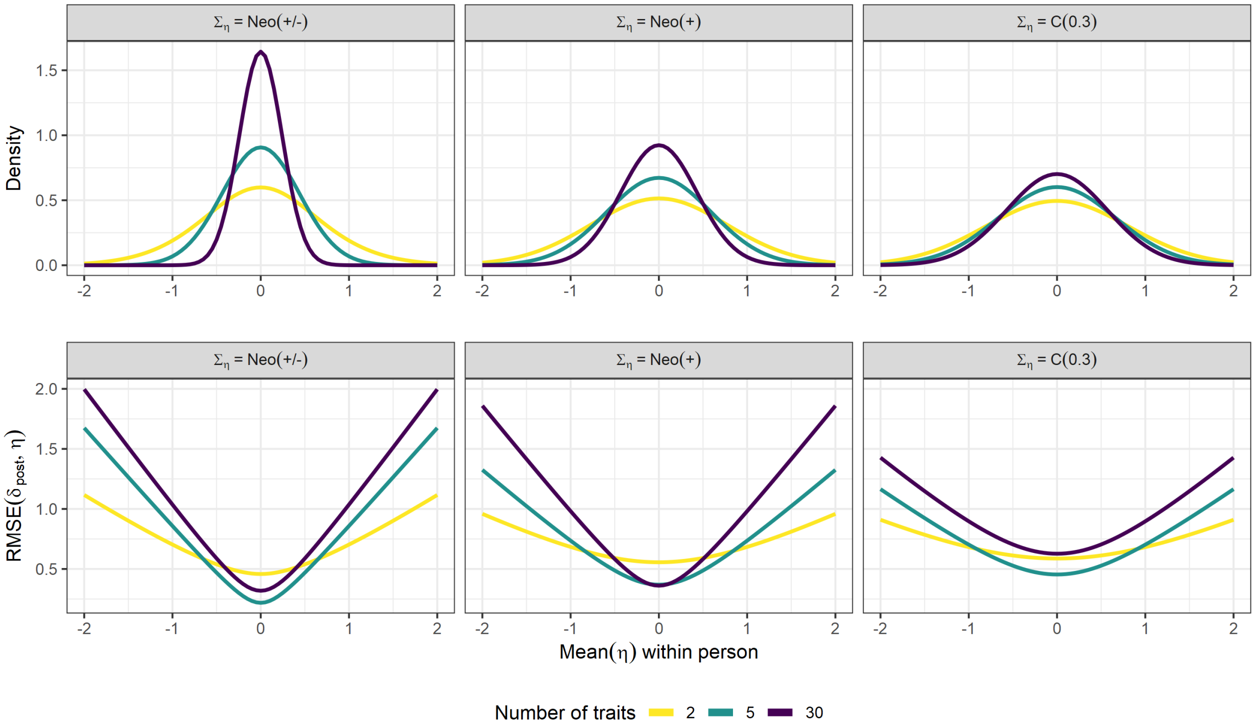

Figure 14. Density (top) and expected RMSE (bottom) as a function of the true within-person trait score mean $\bar{\eta}$. Abbreviations: Σ_η = true sampling correlation matrix; $C(0.3)$ = correlation matrix with all off-diagonal elements set to 0.3.

References

Baron, H. (1996). Strengths and limitations of ipsative measurement. *Journal of Occupational and Organizational Psychology*, *69*, 49–56. https://doi.org/10.1111/j.2044-8325.1996.tb00599.x

Brown, A. (2016). Item response models for forced-choice questionnaires: A common framework. *Psychometrika*, *81*, 135–160. https://doi.org/10.1007/s11336-014-9434-9

Brown, A., & Maydeu-Olivares, A. (2011). Item response modeling of forced-choice questionnaires. *Educational and Psychological Measurement*, *71*, 460–502. https://doi.org/10.1177/0013164410375112

Brown, A., & Maydeu-Olivares, A. (2018). Ordinal factor analysis of graded-preference questionnaire data. *Structural Equation Modeling*, *25*(4), 516–529. https://doi.org/10.1080/10705511.2017.1392247

Bürkner, P.-C., Schulte, N., & Holling, H. (2019). On the statistical and practical limitations of thurstonian IRT models. *Educational and Psychological Measurement*, *79*, 827–854. https://doi.org/10.1177/0013164419832063

Bürkner, P.-C., Schwabe, R., & Holling, H. (2019). Optimal designs for the generalized partial credit model. *British Journal of Mathematical and Statistical Psychology*, *72*(2), 271–293.

Bürkner, P.-C., & Vuorre, M. (2019). Ordinal regression models in psychology: A tutorial. *Advances in Methods and Practices in Psychological Science*, *2*(1), 77–101.

Chaloner, K., & Verdinelli, I. (1995). Bayesian experimental design: A review. *Statistical Science*, 273–304.

Costa, P. T., & McCrae, R. R. (1992). *NEO-PI-r professional manual.* Odessa, FL: Psychological Assessment Resources.

Gill, R. D., & Levit, B. Y. (1995). Applications of the van trees inequality: A Bayesian Cramér-Rao bound. *Bernoulli*, *1*(1-2), 59–79.

Lehmann, E. L., & Casella, G. (2006). *Theory of point estimation.* New-York: Springer.

Ostendorf, F., & Angleitner, A. (2004). *Neo-PI-R: Neo-persönlichkeitsinventar nach costa und McCrae.* Göttingen: Hogrefe.

Samejima, F. (1997). Graded response model. In *Handbook of modern item response theory* (pp. 85–100). New-York: Springer.

Schmidt, D., & Schwabe, R. (2015). On optimal designs for censored data. *Metrika*, *78*(3), 237–257.

Tutz, G. (2000). *Die Analyse Kategorialer Daten: Anwendungsorientierte Einführung in Logit-Modellierung und Kategoriale Regression.* Oldenbourg: Oldenbourg Verlag.
